# Worldwide unintentional acute pesticide poisonings: reassessing the occupational and non-occupational burden

**DOI:** 10.3389/fpubh.2026.1825899

**Published:** 2026-09-02

**Authors:** Wolfgang Boedeker, Meriel Watts, Peter Clausing, Emily Marquez

**Affiliations:** 1 PAN Germany, Hamburg, Germany; 2 PAN Asia Pacific, Penang, Malaysia; 3 PAN North America, Berkeley, CA, United States

**Keywords:** agriculture, farmer, morbidity, mortality, occupational, pesticides, poisoning, unintentional

## Abstract

**Background:**

Human poisoning by pesticides has long been seen as a severe public health problem. As early as 1990, a task force of the World Health Organization (WHO) estimated that about one million unintentional pesticide poisonings occur annually, leading to approximately 20,000 deaths. In 2020, a systematic review found that about 385 million cases of unintentional, acute pesticide poisoning (UAPP) were occurring every year. Our aim is to update the annual global estimate of UAPP, based on a systematic review of available data.

**Methods:**

We updated the existing systematic review of the scientific literature to include the period from 2006 to 2023 supplemented by mortality data from WHO. We extracted data from publications and the WHO Mortality Database, then performed country-wise synopses, and arrived at annual numbers of national UAPP. World-wide UAPP was estimated based on national figures and population data for worldwide regions. We conducted a sensitivity analysis to illustrate the effects of different study designs on the global estimate.

**Results:**

In total 139 countries were covered, including 62 countries covered by 214 articles from 2006 to 2023, and an additional 77 countries covered by data from the WHO Mortality Database. Approximately 369,632 annual cases of UAPP were reported by the extracted publications, resulting from 9,182 fatalities and 360,450 non-fatal cases. On this basis, we estimate that about 402 to 433 million cases of UAPP occur annually worldwide, including around 11,000 fatalities. Based on a worldwide farming population of approximately 934 million, this means that an estimated 46% of farmers are being poisoned by pesticides every year. The greatest estimated number of non-fatal UAPP cases is in southern Asia, followed by south-eastern Asia and east Africa.

**Conclusion:**

Along with other estimates, robust evidence is presented that acute pesticide poisoning is an ongoing major global public health challenge. There is an urgent need to recognize the high burden of non-fatal UAPP, particularly on farmers and farmworkers. The implementation of the recommendation by the Council of the Food and Agriculture Organization of the United Nations (FAO), to phase out highly hazardous pesticides (HHPs) could significantly reduce the burden of UAPP.

## 1 Background

An estimated 385 million cases of unintentional acute pesticide poisoning including 11,000 fatalities occur annually world-wide. This was the finding of our previously published study based on a systematic review of the literature and an analysis of mortality data (1). Four years later, the publication was retracted by the journal under protest from us, the authors. The retraction was based on unpublished concerns raised by an anonymous reader and a letter to the editor by Croplife, a lobbying organisation of the pesticide industry. The concerns were focused on “the use of ‘ever’ prevalence of pesticide poisoning to represent annual frequency in the extrapolations” and that “the assumption of annual exposure for countries where the time frame is not reported is unreliable.” ‘Ever’ prevalence in this context was the idea that some survey questions could be interpreted as asking whether the interviewee had ever been poisoned in their lifetime, rather than recently. We, the authors, provided evidence from the estimate indicating that even if the critique was right, it would affect the results only negligibly.^1^ The process raised important ethical questions with regards to what issues merit a retraction, as well as a journal’s duty in supporting and promoting scientific dialogue and debate.

We take this debate as an opportunity to update our review. Besides extending the review period through 2023 and updating the data, we perform a sensitivity analysis to display the effects of different at-risk periods and study designs on the estimate.

## 2 Introduction

Human poisoning by pesticides has long been seen as a severe public health problem. As early as 1990, a task force of the World Health Organization (WHO) estimated that about one million unintentional pesticide poisonings with severe manifestations occur annually, leading to approximately 20,000 deaths (2). Additionally, two million cases were expected to result from intentional self-harm. It was recognized that people in the developing world were particularly affected by the impact of pesticide poisoning and the number of cases was likely higher, as many cases are unreported. Jeyaratnam further estimated 25 million cases of occupational acute pesticide poisonings per year, the bulk of which were not recorded, as most of the affected did not seek medical attention (3). During the last two decades, international bodies have taken up the issue and adopted a number of resolutions and programs to address the detrimental effects of pesticide use, primarily with a focus on self-harm (4, 5). Despite these efforts, global pesticide use has continued to grow steadily to 3.8 million tonnes per year in 2023, a doubling since 1990 (6).

Many peer reviewed authoritative studies have relied on the pervasive but outdated WHO estimates, which were derived using data from the 1980s (7). With respect to self-harm, a systematic review of data from 2006 to 2015 concluded that pesticides account for 14–20% of global suicides leading to 110,000–168,000 fatalities yearly over the period 2010–2014 (8), a marked reduction from the 258,234 estimated for 2002 (9), with the decline being attributed to regulation of some toxic pesticides and a rural–urban population shift. A paper published in 2020 estimated that 14 million people had died from suicide using pesticides since the advent of the Green Revolution in the 1960s (10).

None of the recent policy instruments relating to pesticides have had a focus on acute non-fatal pesticide poisoning. Regrettably the Sustainable Development Goals (SDGs) focus only on deaths when it comes to poisonings, and it would appear that the majority of those deaths are from intentional self-harm (11). One recent review, in summarizing the effects of pesticides on human health, completely omitted mentioning acute effects at all (12). Additionally, publications often fail to differentiate between intentional and unintentional poisonings (13, 14), or between pesticide and other chemical poisonings (15), or are silent on unintentional pesticide poisonings and instead refer exclusively to suicides (16). This lack of attention to acute pesticide poisoning, and especially to acute non-fatal occupational poisoning, may have hampered the development of measures to prevent such poisoning at both national and international levels. Additionally, it ignores the role such poisonings may play in understanding long-term health effects. Acute pesticide poisonings can be indicative of exposures that may lead to chronic outcomes and are deserving of attention for this reason alone (17). As well, other losses are incurred as a result of acute pesticide poisoning—the loss of quality of life, loss of financial security, loss of well-being, and loss of ability to work.

For these reasons, we aimed to carry out a systematic review of the global distribution of unintentional acute pesticide poisoning (UAPP) and to develop a current estimate of annual worldwide UAPP. We focused on occupational exposure, as this issue seems to be the least well understood but is likely to be the most common source of exposure that results in unintentional acute intoxication.

## 3 Methods

To achieve our goal of a current estimate of annual worldwide UAPP, we carried out a systematic review of the scientific literature and additionally used publicly available mortality data from the WHO. This systematic review is based on a protocol (Supplementary material S1) according to the Preferred Reporting Items for Systematic Review and Meta-Analysis Protocols (PRISMA-P) (18) and was conducted following the Preferred Reporting Items for Systematic Reviews and Meta-Analysis (PRISMA) statement (19) (Supplementary material S2). The protocol was set up for the first review period 2000–2018 and carried forward to the second period 2019–2023. The review was not registered. Data on fatalities were from the WHO Mortality Database (20). Data were first extracted from included publications and sources, then we made synopses for each country (Supplementary material S7) and estimated the annual numbers of national UAPP. Finally, the total of annual world-wide UAPP was estimated based on national figures and population data for FAO-defined regions and sub-regions.

### 3.1 Literature review

#### 3.1.1 Search procedure for publications

The automatic search was carried out for the period from 2000 to 2018, and the search was updated for the second review period from 2019 to 2023. The primary sources for this review were the electronic databases PUBMED, EMBASE, and Web of Science. We aimed for broad search categories while also aiming for a manageable number of hits. An orientating PUBMED search was refined by varying the search terms, term truncation and limiting to specific fields. The results were compared and checked against articles known to be relevant for the review. Search terms from missed articles were added, but skipped when results were shifted to more clinical, treatment, or general toxicological issues.

We searched publications using the term “pesticide” or its synonyms or subgroups (e.g., insecticide). We allowed for any reference to human poisoning or health effects by surveys or taken from registers in terms of incidence, prevalence or specified by morbidity and mortality. We did not impose any restrictions on study designs or case identification. The final full search strategy including keywords is detailed in Supplementary material S3. Search results, including abstracts, were stored using the literature data management software Zotero (21) which allows handling of references, abstracts, and full-texts including checking for duplicates.

All authors checked their own collections for eligible papers to supplement the automatic search. Further, articles were identified in the course of the selection and extraction step of the review by inspecting bibliographic reference lists of included papers and citation tracking.

#### 3.1.2 Eligibility criteria and study selection

We included publications reporting on UAPP covering accidental, homicidal and malicious poisoning. We aimed at the number of UAPP per well-defined populations and timespans. Studies dealing exclusively with suicidal pesticide poisonings and studies where UAPP and suicides were not reported separately, were excluded. So were studies on long-term effects such as cancer, if they did not include UAPP. The profiles of pesticide use pattern, exposure factors, and agriculture differ globally and have changed over time. To best capture the current situation, on the basis that pesticide management is likely to have changed considerably since 1990, we chose to exclude data prior to 2006. A later cut-off date was likely to have resulted in too few studies to provide sufficient information for the analysis. The assessment of the eligibility of studies was based on the exclusion and inclusion criteria in Table 1.

**Table 1:** Exclusion and inclusion criteria for assessment of eligibility.

Criteria for	Items
Inclusion	Papers giving the number of UAPP per well-defined population and time span Published 2006–2018, review period 1 Published 2019–2023, review period 2
Exclusion	Papers not explicitly stating results on UAPP Papers exclusively reporting on suicidal poisoning/intentional self-harm Studies on long-term effects such as cancer that did not include UAPP Studies on poisoning treatments or clinical outcomes Modelling or simulation or biochemical studies Ill-defined survey populations or hospital data with unclear catchment area Language other than English, German, or Spanish Data prior to 2006

All references resulting from the automatic search were screened by title and abstract for eligibility of studies. Papers that appeared to meet the eligibility criteria by their abstract, or did not offer sufficient information to decide, were obtained in full text. Eligibility of the full-text articles was then assessed independently by two reviewers per paper. If there was disagreement, consent was sought by discussion with the entire team of authors.

#### 3.1.3 Data collection

Data from eligible publications were extracted according to the following principles:

- Across pesticides: if UAPP figures were given only for several specific pesticides (e.g., insecticides, fungicides) or for active ingredients we extracted the overall number of cases (e.g., insecticides + fungicides).
- Across types of poisoning: if UAPP figures were provided specifically for several types of poisoning (e.g., accidental, homicidal) we summed up and extracted the overall number of cases.
- Across years: if UAPP were provided for multiple years we calculated the average of cases over the latest years (maximum of 5); we annualized UAPP when reported for a shorter period.
- Across symptoms: if UAPP figures were given for specific symptoms but without ‘overall’ figures, we selected the symptom with the highest prevalence and used those case numbers, noting where UAPP is based only on highest prevalence, any symptom, or two or more symptoms.

We excluded studies that exclusively provided data prior to the year 2006. If an article provided data both prior to and after 2006, only the data from 2006 and later were extracted. If yearly data were not given, a group decision for eligibility was sought. If a publication reported on more than one study or gave data for several use-types (e.g., rural, urban) we extracted one record for each type. If a publication gave the number of UAPP of the survey sample but also provided a national estimation, we extracted the national UAPP figure.

A MS-Excel sheet was drafted for the extraction of data and subjected to a series of pilot test extractions by all four reviewers. Causes of disagreements between reviewers’ test extractions were discussed and led to revised versions of the extraction sheet (Supplementary material S4). Data extraction was done independently by two reviewers per paper. If there was disagreement, consent was sought by the entire team of authors.

We studied the distributions of the fatal and non-fatal UAPP as well as the study characteristics across populations using descriptive statistics. To highlight associations of the prevalence of UAPP and study characteristics, we calculated adjusted prevalence rate ratios. For this purpose, we carried out a multiple Poisson regression with random intercepts, as we assumed that the studies are heterogenous even beyond the extracted characteristics. This analysis was confined to surveys of the farming/occupational population. Data analysis was carried out with SAS statistical software, Version 9.4 (SAS Institute, Inc., NC, USA).

#### 3.1.4 Risk-of-bias assessment

Our review aimed to estimate the global distribution of UAPP. Our concerns about study bias therefore were focused on the prerequisites for a valid extrapolation from a study population to the national level and from the national level to the international region. A risk-of-bias assessment can help to select studies in the data synthesis step, especially when more than one study is available for the same categories of reporting. Our risk-of-bias assessment was directed to systematic differences of the study population (i.e., how participants were selected or recruited) and the target population, as well as to systematic differences in the determination of poisoning. These bias types were assessed by extracting information on the sampling procedure, the identification and evaluation of poisoning.

National estimates for nonfatal UAPP in the farming/occupational population are mainly based on publications reporting on surveys. To study the impact of study quality on the global estimations, we summarized study characteristics and built a sum score for each respective country. All appropriate studies for each country were combined and the highest values for each quality criterion are reported. The sum score then is built by the number of studies considered in the estimations and set values for the review period covered as well as for the single quality criteria (Table 2). Finally, we stratified the global estimations for countries based on certain characteristics:

- Sample 1: all countries with sum scores > = 50% percentile only,
- Sample 2: all countries with sum scores > = 75% percentile only,
- Sample 3: all countries not based on studies with an ‘ever’ at-risk-time,
- Sample 4: all countries not based on studies with an ‘ever’ or ‘unspecified’ at-risk-time.

**Table 2:** Quality scoring.

Criteria	Score
Studies used for calculation	(n)
Review periods covered	
First	1
Second	2
Both	3
Estimates based on studies with a	
Representative sample/register (hospital or other)	1
Simple sample or not stated	0
Diagnosis	
By study scientist	2
Self-reported	1
Latency period	
48 h	3
24 h	2
other	1
Unspecified	0
At-risk time	
Annual	3
< 1 year	2
Unspecified	1
Ever	0
No. of symptoms	
2 or more	2
At least 1	1
Unspecified	0

### 3.2 WHO mortality database

In addition to the data provided by the publications, we extracted mortality data for UAPP based on national statistics from the WHO Mortality Database using the latest available update, from 21 February 2024 (20). The data comprise deaths registered in national civil registration systems with underlying cause of death as coded by the relevant national authority. An underlying cause of death is defined as “the disease or injury which initiated the train of morbid events leading directly to death, or the circumstances of the accident or violence which produced the fatal injury” (20). The mortality database allows for extracting cause of death data by country, year, sex and age.

Most countries report cause of death data using the International Classification of Diseases revision 10 (ICD10). WHO allows reporting with either a 3-digit or 4-digit code. On a 3-digit level the ICD10 code “X48” refers to “Accidental poisoning by and exposure to pesticides,” with a fourth digit that indicates the site where the incident occurred. The ICD10 code “X487” stands for farms and ranches but includes only non-residential buildings and land under cultivation, whereas “farmhouses and home premises of farm” are excluded from this coding (22). The coding of the location of poisonings is dependent on contextual information of the specific poisoning incidents; however, these data are often not available to the coding institution. In this case, countries reporting at the 4-digit level make use of “x489 unspecified place.” Countries report only for ICD codes with incidents; a missing code was considered as zero incidents for our purposes. So, if a country reported by ICD10 3 or 4-digit codes (see Supplementary material S5 for details), but provided no ICD10 code x48, we recorded this as x48 = 0, meaning zero incidents. Additionally, for those reporting by 4-digit codes but providing no code x487, UAPP with farms as the place of occurrence were considered to be zero (x487 = 0).

Data for all countries reporting by ICD10 were extracted for the most recent 5 years. However, when no recent data after 2016 were available for countries that were included in the first review period, we carried forward the data. This was the case for Jamaica, Morocco, and Venezuela. We averaged the crude numbers of UAPP per country over the included years. In addition to all fatalities, children up to 15 years of age and poisonings at farms were considered separately.

Data coded by ICD revision 9 were excluded from analysis because there are no extensional codes to identify accidental pesticide poisoning. Countries reporting by a WHO prepared aggregated code-list also do not have codes that identify accidental pesticide poisoning, so these data were excluded as well.

### 3.3 Synopses and estimation of national UAPP

If no national figures on UAPP were given by the extracted data sources, we extrapolated from study populations by applying the ratio of UAPP (the number of cases per population size) to the respective national population. If these ratios were available from more than one study, we used the average. We guided the extrapolation as close as possible to the study population, so, for example, we did not extrapolate to the entire population when the study base was farmers.

When data from more than one source were available per country we prioritized:

1 National figures.
2 The more general approach.

- On pesticides (e.g., reporting on pesticides in general in contrast to insecticides only).
- On health outcomes (e.g., all UAPP symptoms in contrast to ocular effects of UAPP).
- On populations (e.g., all farmers, in contrast to only female farmers).

3 More recent data.
4 Studies with less risk-of-bias (e.g., with representative samples and verified diagnoses) given the same study characteristics.
5 Studies reporting annual figures in contrast to other at-risk-times.
6 Studies with an ‘ever’ at-risk time were not considered, when data on other at-risk-times were available.

We reported fatal and non-fatal cases of UAPP on three types of populations: general (the ‘all’ population category from Supplementary material S4), farming/occupational (includes ‘farmers & workers’, ‘farmers only’ and ‘workers only’) and children (<15 years). Data on the respective national populations were searched for via the internet if not provided by the extracted publications. We looked for population data most closely matching the studied population and study period. Alternatively, we used the World Bank database (23). To this end, we extracted population data for all available countries and the years matching our review periods. We used the figures on the overall population (indicator SP.POP.TOTL), children (SP.POP:0014.TO), the size of total labour force (SL.TLF.TOT.IN) and the percentage of employment in agriculture (SL.AGR.EMP.ZS). We calculated the size of the ‘farming/occupational’ population by multiplying the share of agriculture by the total labour force. When particular countries or years were missing from the World Bank data, we used data for the total and children population from the UN Population Division Data Portal, but no farming/occupational population data were available from this source (24).

All national synopses were drafted by a team member and checked by a second with respect to comprehensibility, used data sources, and calculations. When in doubt, agreement from all authors was sought.

### 3.4 Estimation of international UAPP

A list of countries and their allocations to regions and sub-regions is available from the UN Population Division Data Portal (24). As data were not available for all countries, we built groups by aggregating Australia and New Zealand together with Melanesia, Micronesia and Polynesia into one sub-region, Oceania. Furthermore, we combined southern and middle Africa, as both sub-regions were poorly covered by national estimates. A table of countries by regions along with the data sources used can be found in Supplementary material S5.

We based the estimation of annual worldwide UAPP on the national estimates. The country specific case numbers were summed per region and the respective sums multiplied by the share of the countries’ populations to the overall population in the regions for a reference year 2022. In detail:

For each sub-region (j = 1,…, m) and country (i = 1,…, n_j_) with national UAPP cases_i_ we

- Summed the country specific cases for region j

$$\text{case}s_{j}=\sum_{i=1}^{\mathrm{nj}}\text{case}s_{i}$$

- Calculated weights w_j_ of the population size of respective countries to the overall population in region j (POP_j_)

$$w_{j}=\frac{\sum_{i=1}^{n_{j}}\mathrm{po}p_{i}}{\text{POPj}}$$

- Estimated cases per region by division of weights j

$$\text{case}s_{\mathrm{estj}}=w_{j}^{-1}\;\text{case}s_{j}$$

We applied this procedure separately for fatal and non-fatal UAPP. For fatal UAPP, we restricted the extrapolation to the general population, since occupational fatal UAPP cannot be correctly assessed by ICD codes. For non-fatal UAPP, we based the extrapolations on the farming/occupational population because this population was well covered by studies.

## 4 Results

### 4.1 Selection procedure

**Figure 1:**
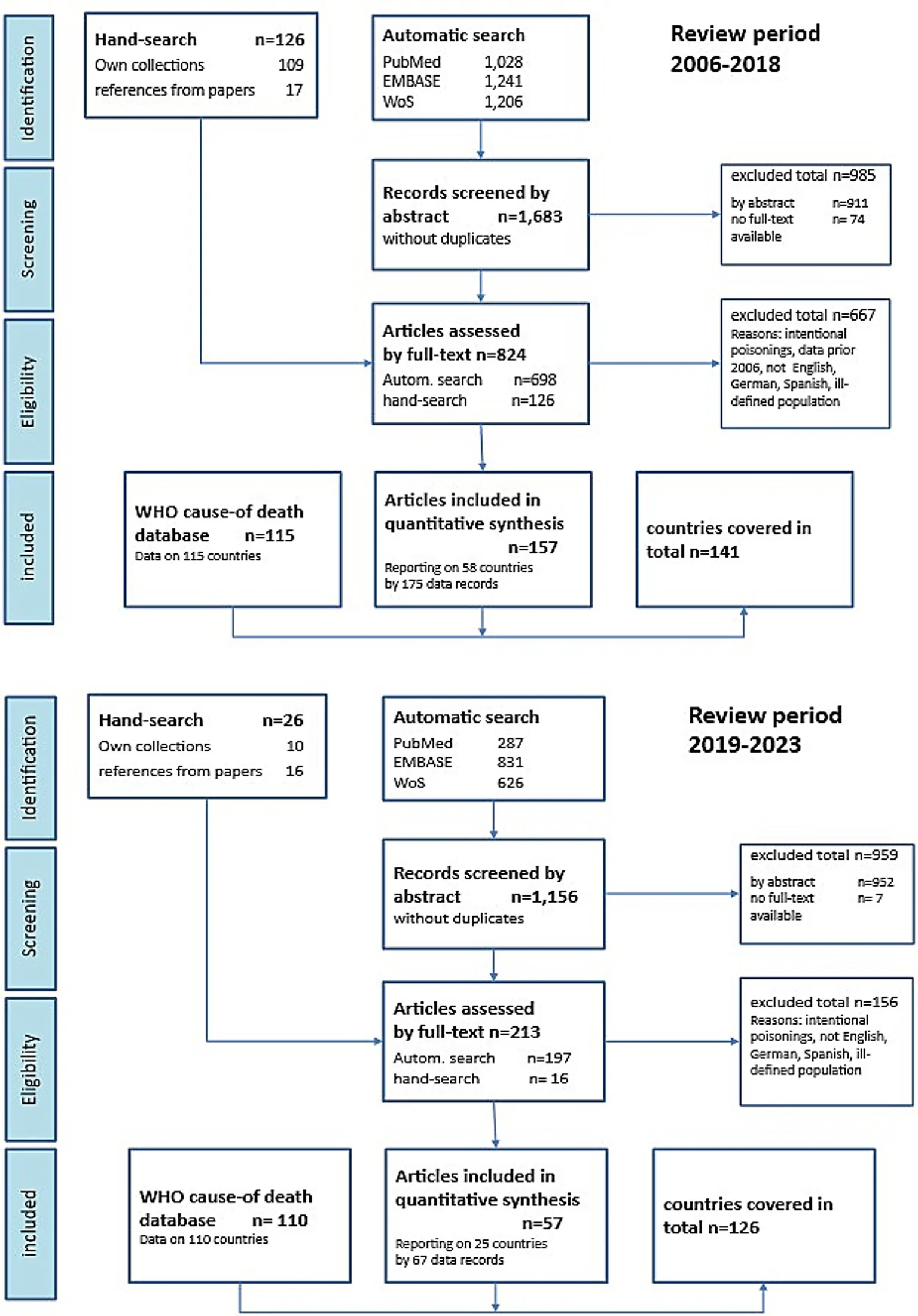
Flowchart for results of the selection procedures for studies and data.

Results of the selection procedure are given in Figure 1. In the review period from 2006 to 2018, we screened 1,683 references by abstract, of which 985 were excluded. Of these, 74 articles could not be obtained because they were published in journals not listed in PubMed, or in journals not accessible by German libraries, or were written in Chinese language (a list of non-accessible papers is available from the authors). Following the screening of abstracts and the hand-search, 824 articles were subjected to full-text assessments. Finally, 157 articles were included in our data synthesis from the 2006 to 2018 review period. As some articles reported on several countries or populations, 175 records were extracted from articles for national synopses.

In the review period 2019–2023, we screened 1,156 records and excluded 959 by abstract or because the papers were not available. There were 213 articles assessed by full-text and 57 of them were included in quantitative synthesis. This review period gained data on four more countries (Hungary, Japan, Lebanon, Turkey). For both review periods taken together, the synthesis is based on 214 articles (25–238) covering 62 countries. The paper of Lekei et al. (99) is a re-analysis of an earlier study (98) and therefore the data were not extracted, but were considered in the country synopsis.

Recent WHO mortality data were available for 110 countries, which is five less countries than originally extracted in the first review period. In total the mortality data covered 139 countries. Almost 50% of these countries were covered by WHO mortality data including the farming/occupational population, while 20% were covered by WHO data and data from articles, 15% had data from articles only, and 7% were covered by mortality data only.

### 4.2 Study characteristics of extracted publications

Extracted data from all included publications are given in the Annex, Supplementary Table S1. A description of study characteristics is shown in Table 3. Most studies focused on occupational poisoning, with farmers and agricultural workers addressed in 151 of all 242 records. There were 68 records referring to the general population (that is, without any subgroup stratification) and 23 records highlighting poisoning in children. UAPP figures on a national level were primarily for the general population, with the data mostly originating from mortality registers, poison-call-centres, or hospital discharge statistics. Data for the farming/occupational population, in contrast, were addressed predominantly in surveys conducted on a district level. Cases of child UAPP were primarily data from poison control centres.

**Table 3:** Study characteristics of included studies on UAPP.

Characteristic	Children population		Farming/occupational population		General population	
	N	%	N	%	N	%
Region						
District	12	52	144	95	24	35
National	11	48	7	5	44	65
Study type						
Poison control centres (PCC)	10	43	3	2	23	34
Hospital	3	13			14	21
Register	7	30	8	5	31	46
Survey	3	13	140	93		
Sampling						
No selection	19	83	11	7	65	96
Representative sample	2	9	94	62	1	1
Simple sample/not stated	2	9	46	30	2	3
Diagnoses						
ICD codes	5	26	1	1	18	26
By study scientist	11	48	18	12	40	59
Not stated	3	9	4	3	8	12
Self-reported	4	17	128	85	2	3
Number of symptoms						
At least one	1	5	22	15	2	3
>1			2	1		
2 or more			7	5		
Not specified	22	96	120	79	66	97
Latency period						
24 h			8	5		
48 h			14	9	1	1
Not specified	23	100	99	66	67	99
Other			30	20		
At-risk-time						
Annual	15	65	36	24	46	68
Ever	1	4	18	12		
Not specified	3	13	80	53	22	32
Other	4	17	17	11		

The studies used varied designs, most of them conducted surveys with random sampling to achieve some representativeness of the study population (see examples in Table 4). Case identification of UAPP was done in 39% of the studies by study scientists or by ICD codes from registers. However, in most surveys on the farming/occupational population, poisoning was self-reported to field researchers who provided a list of pesticide intoxication symptoms. Studies showed some variability in case definitions. Whereas many studies seemed to have made use of the WHO standard definition, e.g., (84, 130), the majority of surveys did not specify what number of symptoms is required to identify an acute poisoning. In some studies, a more restrictive approach to identifying UAPP was chosen, for example in (127), where the survey requested at least three symptoms and excluded pre-existing disease conditions.

**Table 4:** Examples of sampling strategies used in studies.

Quotes from studies
**Without random sampling**: “Present study was conducted in the southern Punjab, i.e., Multan and Bahawalpur Divisions, the major cotton growing areas of Pakistan. The field study was limited to a manageable geographical area where female cotton pickers are living and have a great potential to be exposed to pesticides. The villages selected on the willingness of the female workers that participate in the study … After preliminary survey two female groups (13–35 years of age) were selected as cotton pickers and non-pickers (30–37 females in each group) from the selected area” (159).
“Participants were recruited with the assistance of community leaders, churches, and local groups in the study area. Letters were sent to each of these entities which contained a clear explanation of reasons for the study, study objectives, inclusion criteria, consent to participate, and voluntary participation. These leaders and groups made announcements to the general public or community gatherings for a month. Those farmers who expressed interest in participation were invited to meet at the community leaders’ residence, group meeting locations, or church premises. At these meetings, the principal investigator reviewed the study and explained the content. If the farmer wished to participate, the consent form was signed, and the questionnaire was given to complete” (160).
**Without random sampling**: “From a universe of approximately 3,500 subjects, a random sample of about 1,100 workers directly exposed to pesticides was performed, considering as such those subjects who mix/load and/or apply pesticides.… As mentioned, applicators are professional workers authorized by the Agriculture, Livestock and Food Ministry to perform their tasks. They usually work in several extensive crops in the same area of the province, as independent professionals (the owners of the machinery) or as employees of an agrarian company” (43).
“The 2005 and 2006 surveys were conducted by a market research company and included 6,359 users in 24 countries … Approximately, 250 users were sampled from each country. In each country, a local market research team identified regions where the use of pesticides was moderate to intensive… The selection of respondents was on the basis of quota sampling and targeted users on smallholdings of below average size and contract spray operators in countries where there were significant numbers of such users. The local market research teams designed their target smallholder farmers in terms of farm size and typical crops grown. Screening questions were used to ensure that the sample satisfied the quota requirements” (166).
“The target population of this survey included male farmers residing in rural areas in South Korea. The sampling frame for this survey was constructed by use of 2010 Korean Agricultural Household Registry data. Primary sampling units were formed out of the local administrative districts. We stratified primary sampling units into three strata based on three variables, which were the number of farm households, the farm household population by age group (<15, 15–65, >65) and the proportion of households residing in apartments. The selection of a 3% limit of error in the estimate yielded a needed sample size of roughly 2,000. A total of 197 primary sampling units were selected by probability proportional to size sampling method. In the final sampling stage, the sample size in a primary sampling unit was 10. Trained interviewers visited each selected household and explained about the study” (94).

More than two-thirds of the studies do not specify the latency period from exposure to onset of symptoms under investigation. About 10% make use of the 48-h latency period proposed by the WHO. About 20% of the publications used other latency periods; most were focused on symptoms which appeared during or immediately after spraying. In contrast, a limited number of studies allowed for a delayed latency of up to 15 days (200), or even a month (146). Other studies left it to the respondents to link symptoms to exposure, e.g., (166) asking probands if they “had ever experienced incidents related to agrochemicals.” The at-risk time denotes the period when pesticide exposure could have put persons at risk for pesticide poisoning. The majority of surveys (53%) do not specify the at-risk time, whereas an annual prevalence of UAPP is studied in about 25% and a lifetime prevalence in 12% of the studies. About 9% of the publications used other at-risk time periods that ranged from months to years.

### 4.3 UAPP reported in extracted publications

In total, 1,261,928 cases of UAPP were reported by the included studies from the two review periods resulting from 15,671 fatalities and 1,246,257 non-fatal cases (Table 5). Only eight records provided data on fatal UAPP for children and five for fatal UAPP in the farming/occupational population. For some countries we found repetitive annual reports from the registers, which affects the above total cases of reported UAPP. Excluding the repetitive yearly register data (India, USA) gives a total of 9,182 fatal and 360,450 non-fatal UAPP cases reported by the included studies from the two review periods (Table 6).

**Table 5:** Reported cases of UAPP by population.

UAPP		Children	Farming/occupational	General ^a^	All
Fatal cases	N records	8	5	33	46
	Mean	23	32	465	341
	Median	0	4	4	1
	Sum	181	159	15,331	15,671
	Min	0	0	0	0
	Max	178	98	7,805	7,805
Nonfatal cases	N records	22	148	57	227
	Mean	7,916	1,575	14,721	5,490
	Median	50	92	420	101
	Sum	174,143	233,042	839,072	1,246,257
	Min	0	6	2	0
	Max	36,631	209,512	77,690	209,512

a According to types given in publications, when studies did not differentiate between the sub-populations, “general” includes both “children” and “Farming/occupational”.

**Table 6:** Fatal and non-fatal UAPP according to different estimation steps.

Sum of cases	Fatal	Non-fatal
Extracted publications^a^	9,182	360,450
National estimates^b^	8,674	358,622,020
Worldwide estimates^b^	11,413	433,682,867

a Only the latest year was counted when repetitive yearly register data were available.

b Based on general population for fatalities and on the farming/occupational population for non-fatal UAPP.

The distribution of UAPP is strongly affected by maximum numbers. High numbers are reported from some registers. The maximums of non-fatal UAPP in the general population of 77,690 cases and 36,631 cases in children were reported by the United States National Poison Data System (US-NPDS) for 2012 (115). These figures are derived from calls to US poison control centres and include exposures for which the outcome is unknown. The US-NPDS report gives data for certain outcome categories but does not differentiate the unintentional cases. Also, the follow-up of the medical outcomes was done in less than 50% of the calls, according to the US-NPDS report. In South Korea, the maximum number of the farming/occupational population’s non-fatal cases was 209,512, derived as the nationwide estimation of a representative survey of male farmers and reported by Lee et al. (94).

For fatal UAPP, a maximum figure of 7,805 cases was reported for India in 2021, according to the government’s National Accidental Deaths and Suicides Report (219). Fatal UAPP data were available from the extracted records, mainly for the general population. A smaller number of studies provided data on fatalities for sub-populations, with eight studies reporting a total of 181 deaths in children and five studies reporting a total of 159 deaths in the farming/occupational population. However, the India government report mentioned above reported almost all the extracted fatalities but did not differentiate between sub-populations. Therefore, it seems highly likely that the figures for fatalities in the 13 studies with data on sub-populations are underreported.

Our review has a good coverage of non-fatal UAPP for the farming/occupational population, mostly reported by surveys on specific study populations in specific agricultural regions. Table 7 provides details for the statistical distribution of the reported prevalence of UAPP and shows an average prevalence of non-fatal UAPP of 49%, with prevalence ranging from 2 to 100%. However, the prevalences vary according to study characteristics. There seems to be a strong influence of the sample size, e.g., sample populations that were less than *n* = 132 (1st tertile) had a higher prevalence than studies in the 3rd tertile with sample sizes greater than *n* = 332 (60% vs. 36% prevalence, respectively). Adjusted for all characteristics in Table 7 the prevalence rate ratio was greatest in the 2nd tertile (1.74), indicating multiple correlations between the characteristics. When the case definition was rather strict, e.g., requiring 2 or more symptoms present, then the mean prevalence was only 20%, compared to 50% when only one symptom was required. The mean lifetime prevalence was 34%, compared to 41% for an annual at-risk-time. Only two study characteristics showed a statistically significant effect: the ‘ever’ at-risk time and the smaller sample size. For these, the 95% confidence intervals of the risk ratios did not include one.

**Table 7:** Reported prevalences for the farming/occupational population by study characteristics.

Characteristic	N	Mean	Median	Min	Max	RR^a^	RR LCL^b^	RR UCL^b^
All	140	0.49	0.44	0.02	1.00			
Sample size								
1st tertile:<=132	48	0.60	0.59	0.07	1.00	1.37	1.18	1.60
2nd tertile: 132–332	46	0.50	0.43	0.14	1.00	1.74	1.38	2.21
3rd tertile: >332	46	0.36	0.34	0.02	0.88	1.00		
Sampling								
Representative sample	94	0.45	0.39	0.02	1.00	0.93	0.73	1.18
Simple sample or not stated	46	0.56	0.57	0.14	1.00	1.00		
Diagnoses								
By study scientist	12	0.43	0.39	0.02	1.00	0.81	0.55	1.19
Self-reported	128	0.49	0.44	0.06	1.00	1.00		
Number of symptoms								
2 or more	7	0.20	0.16	0.10	0.35	0.63	0.37	1.08
At least one	21	0.50	0.52	0.06	1.00	0.96	0.71	1.30
Not specified	112	0.50	0.47	0.02	1.00	1.00		
Latency period								
24 h	8	0.38	0.23	0.14	1.00	0.78	0.48	1.27
48 h	14	0.32	0.23	0.10	0.91	0.91	0.62	1.34
Other	28	0.55	0.55	0.14	1.00	1.17	0.89	1.54
Not specified	90	0.50	0.46	0.02	1.00	1.00		
At-risk-time								
Annual	27	0.41	0.35	0.08	0.85	0.74	0.52	1.04
Ever	18	0.34	0.29	0.02	0.93	0.54	0.39	0.75
Other	17	0.46	0.43	0.10	0.96	0.82	0.58	1.16
Not specified	78	0.55	0.52	0.14	1.00	1.00		

a RR: rate ratio by fully adjusted random effect Poisson regression.

b RR LCL, RR UCL: lower, upper 95% confidence limits.

### 4.4 UAPP reported in WHO mortality database

Mortality data could be extracted for 110 countries reporting by ICD10 using three or four-digit codes. Of these, 68 provide data on UAPP (Supplementary material S6). There were 21 countries that reported UAPP on a three-digit level only (x48) so no information on the place of incidents was available, meaning no farmer/farmworker populations could be identified. No entries for x48 were given for 42 countries, so zero UAPP was assumed. Almost all countries contributed data for several years before 2022. However, for some countries data for only 1 year was available (Supplementary material S6).

Overall, 722 yearly fatalities due to UAPP were notified, 70 of these occurring in children and 15 in the farming environment (Table 8). The distribution is rather uneven, with a country maximum seen in Guatemala resulting from 585 fatal cases occurring from 2017 to 2021. The highest number of yearly fatalities in children was reported by Mexico, with 78 fatalities over the same period. For farms, data on fatalities were available from 95 countries, with 82 countries reporting zero UAPP. However, this may be due to missing information on the place of occurrence of UAPP, with farm accidents not being coded. Among the ten countries with the highest number of annual fatal UAPP, six were from South and Central America.

**Table 8:** Annualized fatalities from UAPP reported by countries according to WHO mortality database.

Population	Countries	Annualized fatalities			
	N	Mean	Median	Sum	Max
General	110	6.6	0.5	722	117
Children	110	0.6	0.0	70	16
Farming	95	0.2	0	15	5

### 4.5 National estimates of UAPP

To derive national estimates of UAPP, all extracted papers and WHO mortality data were revisited for each country. For the 62 countries covered by publications, the country synopses specify which data were used for national estimates and highlight specific limitations of the data used and estimations (Supplementary material S7).

UAPP data were rarely reported by more than one paper per country for the general and child populations. An exception is the USA, with yearly reports of poison control centres and other institutions. The data sources were different for non-fatal UAPP in the farming/occupational population, with most of the countries covered by more than one publication and a maximum of 15 for India. In general, the studies in our review vary widely with respect to the study populations, assessment of poisoning, years, and between countries. For the national estimates we therefore refrained from any weighting of outcomes, as this could enhance the many differences of the study designs and bias the overall results. For Albania and Zambia, no national estimates were derived although publications were extracted (158, 176), because the studies reported data with no clear catchment area stated and therefore were not considered to be reliable for a national estimate. For four countries (France, Hungary, UK, Zimbabwe), we relied on publications reporting only a lifetime prevalence of UAPP, as no other studies were available. However, we addressed the countries that only had lifetime prevalence data in our sensitivity analysis (see Table 9), detailed in Section 3.1.4.

**Table 9:** Results of sensitivity analysis—non-fatal UAPP for the farming/occupational population according to different sample data sets.

Sample	Population in Region	Population in review	Countries in review	Summed non-fatal cases	Estimated non-fatal cases
0: All countries	934,246,829	750,551,397	49	358,622,020	433,682,867
1: Summed quality score >p50	934,246,829	699,155,262	27	328,486,359	420,312,136
2: Summed quality score >p75	934,246,829	444,635,053	13	265,162,984	402,255,273
3: Without ‘ever’ prevalence	934,246,829	745,818,709	45	356,485,601	431,750,327
4: Without ‘ever’ prevalence and ‘unspecified’ at-risk times	934,246,829	666,406,695	31	308,356,811	423,471,884

All country specific synopses were collated, including those countries for which no data were available from extracted publications, but for which there were WHO mortality data available (Supplementary Table S2). Supplementary Table S2 reports for each country on the above-mentioned population categories (general, children and farming). The numbers of fatal and/or non-fatal UAPP are given, along with the years and size of the respective populations. Across all countries, a population of approximately 5.5 billion is covered, with approximately 900 million children and approximately 750 million in the farming/occupational population. For all countries for which we had data, we arrived at approximately 358 million cases of UAPP annually (Table 6). This overall figure resulted from very different national estimates. For example, for the farming/occupational population the highest estimated number of non-fatal UAPP was 169 million for India, resulting from a prevalence ratio of 73% and a reference farming population of 230 million. This prevalence was based on the mean of 11 publications. In contrast, the minimum number of 270 non-fatal cases was for Australia, with an estimated farming population of app. 350,000. This national estimation was derived from one paper of the Poison Centre for the State of Victoria.

### 4.6 Worldwide estimates of UAPP

The World Bank provides population data on 218 countries with a total population of approximately 8 billion covered by this study in the reference year 2022. Of this population, 69% is represented by national estimates of UAPP. However, the coverage differs with respect to fatal and non-fatal UAPP.

For fatal UAPP, 116 countries with national estimates in our review represented a population of around 5.5 billion (Table 10). The national estimates summed to an overall fatal UAPP of 8,674, which by our extrapolation procedure resulted in 11,413 fatalities worldwide annually. The vast amount of these fatalities is expected to occur in southern Asia, which is covered in this review by four countries with 76% of the population in this region. Western Africa is not covered in this estimation; for eastern Africa the national estimation, although based on four countries, represents only 14% of the regional population.

**Table 10:** Estimated worldwide annual fatal UAPP by region.

Region	Population in region	Population in review^a^	Weight^b^	No. of countries^a^	Sum fatalities in review	Estimated fatalities in region
Africa, Eastern	472,857,319	67,937,080	0.14	4	8	55
Africa, Middle-Southern	264,676,586	59,893,885	0.22	1	12	51
Africa, Northern	259,393,961	167,616,532	0.64	4	76	117
Africa, Western	429,079,551	0	0.00	0	.	.
America, Caribbean	44,256,232	30,070,868	0.67	17	8	11
America, Central	178,574,337	167,736,205	0.93	6	241	256
America, Northern	372,280,991	372,280,991	1.00	3	11	11
America, South	437,531,089	424,688,939	0.97	11	191	197
Asia, Central	78,628,529	72,197,759	0.91	4	7	8
Asia, Eastern	1,641,815,999	1,592,326,472	0.96	4	292	301
Asia, South-eastern	680,786,398	227,280,284	0.33	5	52	156
Asia, Southern	2,007,898,570	1,528,428,530	0.76	4	7,715	10,135
Asia, Western	293,423,308	181,491,334	0.61	13	21	33
Europe, Eastern	283,846,656	99,847,738	0.35	8	21	60
Europe, Northern	105,943,641	105,943,641	1.00	10	5	5
Europe, Southern	149,518,127	146,626,954	0.98	11	12	12
Europe, Western	199,702,602	199,626,806	0.99	7	2	2
Oceania: AUS, NZ, Micro -Mela -Polynesia	44,752,375	36,986,596	0.82	4	1	2
All	7,944,966,271	5,480,980,614		116	8,674	11,413

a Countries with data on fatal UAPP.

b w_j_ = population in review divided by region’s population, for details see Section 3.4.

**Figure 2:**
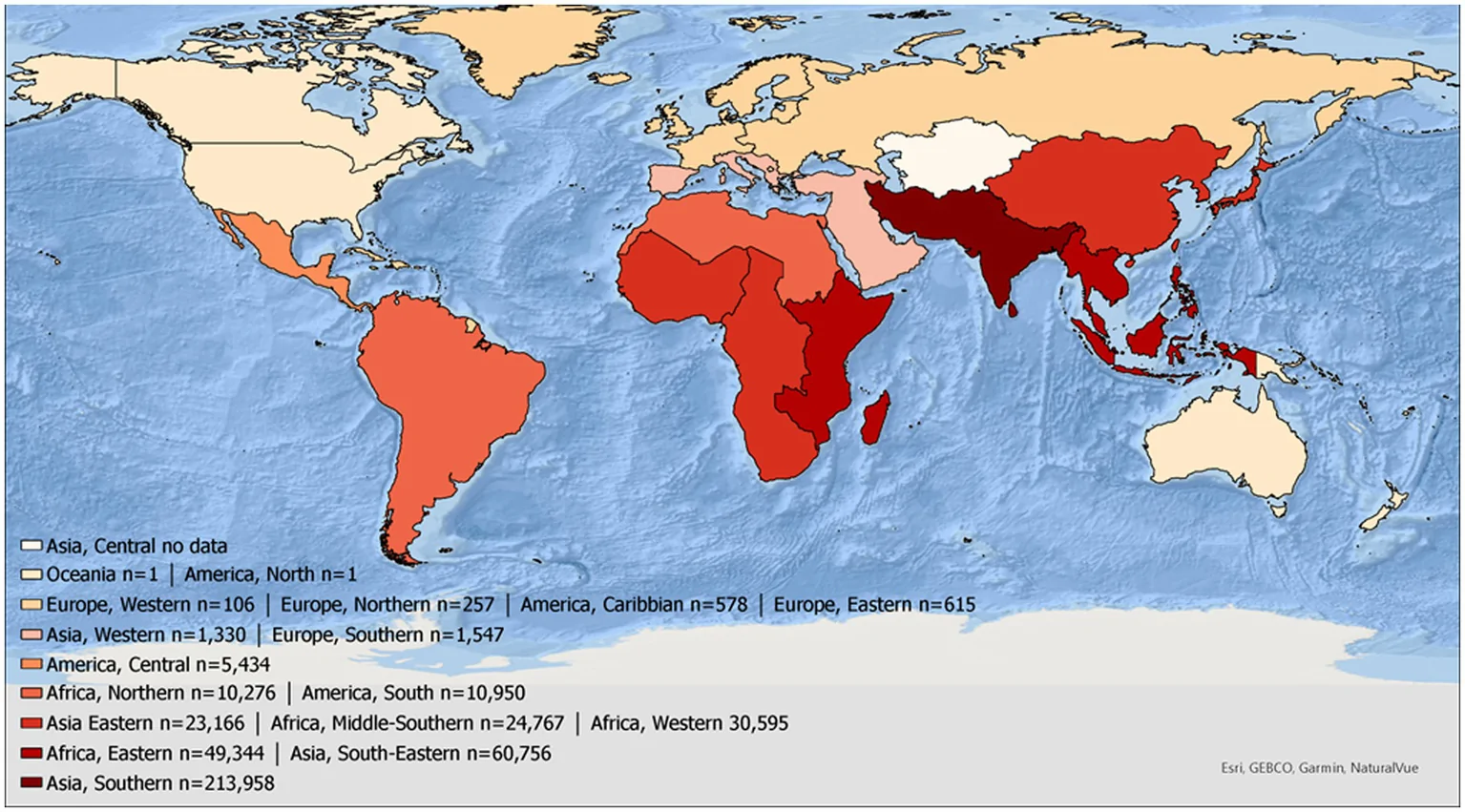
Number (x 1,000) of non-fatal untentional pesticide poisonings in UN Regions (total = 433 million).

With respect to non-fatal UAPP (Table 11), national estimates are available for 49 countries, reporting on 81% of the respective worldwide farming/occupational population. The sum of national estimates of approximately 358 million is extrapolated by our procedure to an estimate of 433 million non-fatal UAPP worldwide annually in the farming/occupational population (Figure 2). The lowest share of countries in this review to the overall population of the region is seen for eastern Europe (2%), which is represented by Hungary only. For some regions, our extrapolation was based on only one country. For middle and southern Africa, figures were based on Cameroon, but its national estimate of non-fatal UAPP was derived from four surveys. No national estimates on non-fatal UAPP were available for central Asia.

**Table 11:** Estimated worldwide annual non-fatal UAPP among the farming/occupational population by region.

Region	Population in region	Population in review^a^	Weight^b^	No. of countries^a^	Sum non-fatal cases in review	Estimated non-fatal cases in region
Africa, Eastern	122,945,489	85,766,297	0.70	6	34,422,194	49,344,016
Africa, Middle-Southern	44,799,935	4,891,694	0.11	1	2,704,345	24,767,386
Africa, Northern	17,360,976	3,746,263	0.22	1	2,217,435	10,276,062
Africa, Western	66,418,224	46,223,581	0.70	6	21,292,849	30,595,492
America, Caribbean	3,943,952	232,019	0.06	1	34,001	577,962
America, Central	12,438,682	7,761,837	0.62	2	3,391,135	5,434,441
America, Northern	3,000,481	2,725,594	0.91	1	1,125	1,238
America, South	26,554,915	18,288,624	0.69	6	7,541,710	10,950,495
Asia, Central	7,192,222	0	0.00	0	.	.
Asia, Eastern	187,604,712	178,055,169	0.95	2	21,986,444	23,165,632
Asia, South-eastern	100,642,772	90,148,998	0.90	7	54,420,819	60,755,662
Asia, Southern	309,778,165	303,152,616	0.98	5	209,381,532	213,957,668
Asia, Western	14,042,686	6,701,088	0.48	5	634,716	1,330,100
Europe, Eastern	9,162,581	217,540	0.02	1	14,608	615,275
Europe, Northern	973,631	345,465	0.35	1	91,350	257,454
Europe, Southern	4,086,587	1,166,937	0.29	2	441,750	1,546,998
Europe, Western	1,889,332	818,096	0.43	1	45,839	105,862
Oceania: AUS, NZ, Micro-Mela- Polynesia	1,411,487	309,578	0.22	1	270	1,231
All	934,246,829	750,551,397		49	358,622,020	433,682,867

a Countries with data on non-fatal UAPP.

b wj = population in review divided by region’s population, for details see text 3.4.

To analyse the influence of survey characteristics on the worldwide estimations, we repeated the analysis for the different samples detailed in section 3.1.4. The estimates of UAPP turned out to be rather stable (Table 9) ranging from a maximum of 433 million cases when all countries were considered (sample 0) to a minimum of 402 million cases, where only those countries which had the highest quality scores were considered (above 75% of all countries, sample 2). The sample 2 estimate comes with a reduction in the number of countries from 49 to 13, which we believe makes the estimates for some regions very limited in scope. A comparison between the sample data sets indicates that there is almost no effect on the estimated UAPP cases if we exclude country estimates that had an ‘ever’ prevalence (sample 3), and only a small effect from further excluding those with unspecified at-risk times (sample 4).

## 5 Discussion

The aim of this paper was to systematically review the literature on the prevalence of UAPP and to estimate the annual global distribution. In total, we estimate that between 402 and 433 million cases of UAPP occur annually world-wide including about 11,000 fatalities. This estimation depends on the quality and validity of data as well as the estimation procedure.

### 5.1 Effects of single estimation steps

Our extrapolations follow a stepwise approach. The effects of the different estimation steps are highlighted in Table 6. For fatal UAPP, almost no difference was seen between the reported numbers from eligible publications and the national estimations, as the data were already on a national level. The world-wide extrapolation added approximately 3,000 cases across all regions.

In contrast, for non-fatal UAPP, a steep increase occurs by extrapolating from numbers in extracted publications to the national level. That is because non-fatal UAPP was mostly recorded by surveys on study populations, and the national estimates resulted from applying the poisoning ratios to larger national farming/occupational populations. So, the estimation of non-fatal country-wise UAPP is a crucial step in our review and depends upon the reliability of assessed incidence of UAPP. We found a mean ratio of 49% of respondents suffering from UAPP based on all of the included surveys (Table 7), with a span of prevalences between two and 100 percent showing high variability across studies, countries, and populations studied. The variability was lower when countries were compared using studies with the same study design. This variability parallels the results of an international survey in 11 countries and across different populations (166). In this survey, the ratio of UAPP was lowest for Spain (30%) and highest for Morocco (85%), pointing to a possible influence of the study designs.

### 5.2 Relation to other estimates of UAPP

Our estimation considerably exceeds the pervasive 1990 WHO figure of about 1 million annual cases of UAPP. The 1990 figure, however, was understood to refer to poisonings with severe manifestations only and relied mostly on hospital data. WHO concluded that the numbers of “poisonings may be matched by a greater number of unreported, but mild, intoxications and acute conditions such as dermatitis” (2). In revisiting the WHO assessments, Jeyaratnam provided an estimate for those unreported, mild intoxications as 25 million cases in developing countries (3). His estimate was an extrapolation from surveys of self-reported symptoms undertaken in just two countries in Asia, in which 6.7% of agricultural workers in Malaysia were poisoned per year and 2.7% in Sri Lanka. In contrast, recent publications for Malaysia report an average prevalence of UAPP among farmers and workers of 43% (184, 231). We were unable to arrive at an occupational estimate for Sri Lanka in the current study. However, our estimate for a yearly UAPP prevalence in the farming/occupational population averaged across countries is 42% (Table 12). Our estimates range from a low of 0.04% in the USA to a high of 84% in Burkina Faso.

**Table 12:** Prevalence of yearly non-fatal UAPP among the farming/occupational population by regions and countries.

Region	Country	UAPP (%)
Africa, Eastern	Ethiopia	26.0
	Kenya	35.1
	Malawi	78.0
	Tanzania	70.8
	Uganda	66.0
	Zimbabwe	45.1
	*Mean*	*53.5*
Africa, Middle-Southern	Cameroon	49.0
Africa, Northern	Morocco	52.9
Africa, Western	Burkina Faso	83.8
	Cote d’Ivoire	20.0
	Gambia, The	51.5
	Ghana	39.0
	Nigeria	69.0
	Senegal	30.5
	*Mean*	*48.9*
America, Caribbean	Jamaica	16.0
America, Central	Costa Rica	32.0
	Mexico	48.0
	*Mean*	*40.0*
America, Northern	United States	0.04
America, South	Argentina	47.4
	Bolivia	45.7
	Brazil	26.0
	Chile	17.6
	Colombia	68.5
	Venezuela, RB	62.0
	*Mean*	*44.5*
Asia, Eastern	China	10.8
	Korea, Rep.	24.1
	*Mean*	*17.5*
Asia, South-eastern	Cambodia	62.0
	Indonesia	62.3
	Lao PDR	66.0
	Malaysia	43.7
	Philippines	57.9
	Thailand	36.0
	Viet Nam	56.3
	*Mean*	*54.9*
Asia, Southern	Bangladesh	46.0
	India	73.6
	Iran, Islamic Rep.	65.0
	Nepal	51.0
	Pakistan	53.0
	*Mean*	*57.7*
Asia, Western	Georgia	20.0
	Kuwait	82.0
	Lebanon	38.1
	Turkiye	11.3
	West Bank and Gaza	34.5
	*Mean*	*37.1*
Europe, Eastern	Hungary	6.5
Europe, Northern	United Kingdom	23.0
Europe, Southern	Portugal	34.0
	Spain	30.0
	*Mean*	*32.0*
Europe, Western	France	6.1
Oceania, AUS NZ Mic-Mel-Polynesia	Australia	0.08
*All*		*42.3*

Apart from the USA, the only other country to register nonfatal UAPP below 1% was Australia. The Australia data came from a register for one state and did not include farmer/worker surveys. Unfortunately, the register-based data from Australia did not allow for differentiation between subpopulations, and the share of the farming/occupational population in the UAPP total is therefore not known. For North America, the respective underestimation of non-fatal UAPP is highly likely, based on the low number of cases in the USA. Only 1,125 cases were reported for the occupational population in North America (Table 11) and yet in the USA alone, about 67,000 cases of non-fatal UAPP occurred annually among the general population (Supplementary Table S2). For the USA, we relied on four studies to extrapolate to the farming/occupational population. We suspect that the low reported percentage for the USA farming/occupational population may be due to underreporting, in spite of having some coverage of agricultural areas by the USA studies that were used for the extrapolation. Factors contributing to underreporting have been discussed in the US EPA’s Recognition and Management of Pesticide Poisonings (17).

In conclusion, our world-wide estimates of UAPP follow from a better coverage of countries and data sources compared to earlier studies. An increase of pesticide poisoning could have resulted from a twofold increase in global pesticide consumption between 1990 (1.8 m tons) and 2023 (3.8 m tons). This includes increases of 210% in the Americas and 185% in Africa, compared to a decrease in Europe of 5% (239). Based on the large increase in global pesticide consumption, many more farmers and workers are now likely to be exposed to pesticides globally, or more often exposed through more frequent use. Our estimates are based on the size of the agricultural population provided by the World Bank, which is calculated by a given share of the total employment. It has to be pointed out that these estimates are probably too low because “employment” is for some countries too narrow a definition, as it might not include informal employment or people engaged in subsistence farming.

### 5.3 Challenges for estimations of UAPP

#### 5.3.1 Comparability of case identification and at-risk times

There is no generally agreed upon definition of acute pesticide poisoning. Studies often refer to a classification tool provided by the Intergovernmental Forum on Chemical Safety (IFCS), which was hosted by the WHO (240). An acute pesticide poisoning according to the IFCS definition is any illness or health effect resulting from suspected or confirmed exposure to a pesticide within 48 h. Clinical presentations and symptoms of poisoning were tabulated by this tool. The chosen latency period from exposure to onset of symptoms is decisive for case identification and comes as a trade-off, especially as unspecific symptoms like headache or nausea are also recognized as exposure effects. A too-short period might exclude symptoms with longer latency, while a too-long period could lead to the recognition of poisoning by symptoms that might have resulted from other causes. Besides the case definition, the studied at-risk-time when exposure might have taken place is also crucial for identification of acute poisoning.

Figures for UAPP in this review originated from registers (e.g., mortality or hospital discharge) or from surveys. Registers usually provide data by ICD codes based on medical records of all defined cases and time span, whereas the surveys identify UAPP by questionnaires applied cross-sectionally to a selected population. Usually, persons are the observation units in surveys and person characteristics are related to the poisonings, whereas from registers, cases are reported and the monitoring of poisoning is the aim. As a person can suffer from repeated poisonings in a given time span, the incidence of cases usually exceeds the incidence of poisoned persons.

Studies included in this review varied with respect to the study design, which affected the prevalence of UAPP by various factors, such as sample size, the case definition, and the at-risk times. Several studies referred to the IFCS definition with differing at-risk-times, e.g., a week (28), or even a lifetime “… whether any of 12 listed symptoms had ever been experienced within 48 h of using such pesticides …” (154). Other studies used their own case definitions focused on symptoms, which can show up immediately after spraying (55), within 24 h (50), or have delayed latency for up to a month (146). Some studies refrained from mentioning any latency time and left it to the respondents to link symptoms to exposure, such as “during application last year” (32), or “had ever experienced incidents related to agrochemicals” (166). Some studies, in contrast, used stricter case definitions compared to IFCS, e.g., by requiring more than three symptoms present and discontinuation of work (127). Some studies analysed effects from different time frames. For example, Choudhary et al. (50) studied poisoning symptoms with respect to different at-risk times. Prevalence of skin related problems was highest in the 18 months exposure group (50%), in contrast to those exposed for 12 months (13%), or for 6 months of exposure (8%). However, no information was given on how often or to what extent pesticides were used in those periods.

Kofod et al. (241) questioned the validity of self-reported symptoms as a proxy for acute organophosphate poisonings. The authors found by a randomized design a high prevalence of nonspecific symptoms, taken from a standardized list of clinical presentations, in the intervention group (chlorpyrifos application) as well as in the placebo group (neem application). The study also found no difference in biomarker plasma cholinesterase (PChE) activity between the groups and after intervention. A surprisingly high percentage of the farmers reported symptoms for a seven-day period which was thought to be a “washout” period without any pesticide exposure. In contrast, other studies found differences in UAPP between pesticide applicators and matched control groups (30, 50, 147).

In summary, it is difficult to assess the influence of individual studies’ characteristics on our estimations because most studies gave no clear case definitions and timeframes. In general, we aimed at annual figures and annualized figures when data for more than one year was provided by registers. However, we made use of survey results as annual prevalence, even when the at-risk time differed. To ensure that results were not biased by long-term exposures, we excluded studies with an ‘ever’ at-risk time from our national estimations whenever surveys with other at-risk times were available. In the case of four countries (France, Hungary, UK, Zimbabwe), the only available studies used an ‘ever’ at-risk time and were used for the estimation. However, our sensitivity analysis (Table 9) indicated that omitting countries with unspecified at-risk times or variation in the quality score had little influence on the estimation of global UAPP figures. Based on our sensitivity analysis, the estimates of UAPP ranged from 433 million cases (sample 0) to 402 million cases (sample 2). We acknowledge that extrapolations might lead to an overestimation of country-wide UAPP by surveys carried out in regions with high pesticide usage or high-risk populations, and by studies that use non-specific symptoms as case indicators.

#### 5.3.2 Underreporting by register and hospital discharge data

Data from registers like the WHO Mortality Database or hospital discharge statistics rely on the utilisation of health services and effectiveness of reporting systems. Both are limited in many countries. These data are likely prone to underestimation, because individuals suffering from acute pesticide poisoning may not seek medical care for various reasons, such as lack of financial capacity, language and cultural barriers, lack of health insurance, inability to take time off from work or fear of losing paid work, lack of access to transportation, or lack of medical facilities (241). The country specific reporting systems might give further causes for underreporting (17) including:

- Lack of a universal, mandatory legal duty to report incidents,
- Lack of a central reporting point for all incidents,
- Similarity of symptoms associated with pesticide poisonings to other causes,
- Misdiagnosis by physicians because of a lack of familiarity with pesticide effects,
- Inadequate investigation of incidents to identify the pesticide that caused the effects,
- Difficulty in identifying and tracking chronic effects,
- Physicians’ lack of knowledge, or inability or reluctance to report incidents,
- Limited geographic coverage of individual poisoning databases.

Studies have examined the number of counted deaths or poisonings against what is likely an underlying greater number of poisonings. A survey conducted in a potato-producing province in Ecuador reported a pyramid of estimated pesticide health impacts with four deaths per year translated to ten hospitalisations per year, with 40 poisonings that reached medical care per year, 400 possible poisonings with no clinical care, and 4,000 cases of prevalent subclinical neurotoxicity with important performance deficits (242). A recent study calculated a factor of up to 71 to correct for underestimation of occupational pesticide poisoning in routine community based surveillance (99).

Finally, we expect a considerable underreporting of fatal occupational UAPP because the respective ICD10 codes were not used or WHO mortality data were not available. For example, a Government of India document (219) reported about 7,805 fatalities in 2021, many of them probably resulting from occupational exposure, but India did not transfer these data to the WHO Mortality Database, nor did the government identify the number of occupational poisonings in its report.

### 5.4 Public health framework—recommendations

Realizing that the conditions of use in developing countries are such that toxic pesticides cannot be used safely, the FAO/WHO International Code of Conduct on Pesticide Management (243) states that “Pesticides whose handling and application require the use of personal protective equipment that is uncomfortable, expensive or not readily available should be avoided, especially in the case of small-scale users and farm workers in hot climates.” In 2006, the FAO Council recommended that consideration be given to the progressive ban of highly hazardous pesticides (HHPs) (244), a call that was supported by the 2015 International Conference on Chemicals Management (ICCM4) (245), and by a FAO/WHO Guideline to the International Code of Conduct on Pesticide Management (243). The lack of action on FAO’s 2006 recommendation and the ongoing problems with pesticides led the UN Special Rapporteur on the Right to Food to recommend to the UN Human Rights Council in 2017 that there should be a comprehensive binding treaty to regulate pesticides throughout their life cycle (4). Finally, the UNEP-based voluntary Global Framework on Chemicals has set a target (A7) of 2035 for the phase-out of HHPs from agriculture, where the risks have not been managed (246). Implementing these recommendations—especially encouraging all stakeholders to implement agro-ecologically based alternatives to HHPs, which was also recommended by ICCM4—would drastically reduce the unacceptably high level of UAPP. Several studies have indicated that phasing out HHPs does not need to result in reduced agricultural productivity (247).

### 5.5 Limitations

In addition to the above-mentioned challenges for estimating world-wide UAPP, our study has some limitations. First, the search strategy might have been too restrictive to identify all relevant publications. We carried out sensitivity tests, e.g., by deleting items or by extending to more specific terms like, e.g., “organophos*” or to active ingredients in pesticides, but these appeared to barely change our results. We further discuss study variability in the studies in the ‘Challenges’ section of the discussion. Further, we might have missed relevant contributions in the grey literature and surely from national or regional poison control centres. Second, our world-wide estimate of UAPP is partly based on a weak database—our literature search yielded the best available data allowing us to conduct a systematic review of UAPP, but some countries were covered by only one publication or by data on small samples sizes of specific study populations.

We have grouped countries in regions and according to UN determination, with the understanding that consistency in types of agriculture, pesticides used and conditions of use that influence exposure is likely to be greater across sub-regions than regions. Overall, studies reported too heterogeneously for global extrapolations to be based on pesticide use pattern. Finally, although deaths from pesticides in food are known to still occur (248), we did not try to estimate them, nor was there any information in the publications we reviewed that could lead to such an estimate.

## 6 Conclusion

Our review, done according to the international scientific PRISMA standards, updates world-wide unintentional acute pesticide poisoning figures, such as the outdated WHO figures from 1990 and our previously published review (1). The estimate in this study complements a recent review on suicidal pesticide poisoning. Taken together, robust evidence is provided that acute pesticide poisoning is an ongoing major global public health challenge. This is despite the efforts over recent years to establish programs to reduce the detrimental effects of pesticide use.

Our results point to a heavy burden of non-fatal UAPP, particularly for farmers and farmworkers, with an estimated 402–433 million cases of UAPP and more than 11,000 deaths per year. This brings into focus the current bias towards focusing only on fatalities and the need to more seriously address the problem of non-fatal UAPP in both the international policy arena and in national pesticide, agriculture, environmental, and health policies.

Estimations of global unintentional acute pesticide poisoning rely entirely on the quality and completeness of available data. Currently available information is inadequate for producing robust, comparable estimates across countries and pesticide use patterns. Existing mortality registers and surveys do not provide consistent, high-quality coverage, hospital discharge records and poison control centre data are rarely consolidated at the national level, and these fragmented systems often fail to feed into national mortality registers. Although part of the peer-reviewed literature contains well-planned surveys and comprehensively describes the methodology used, many others lack sufficient detail in the methods description and/or are less well-planned.

To address these gaps, sustained international support is needed to build and harmonize national documentation and monitoring systems and to expand the coverage of the WHO Mortality Database. Establishing standardized surveillance and reporting protocols is essential so that the information from sources mentioned above can be integrated, cases can be classified consistently, and global estimates of pesticide-related deaths become reliable and comparable. Only with coordinated, standardized data collection and reporting can researchers and policymakers accurately measure the burden and target effective prevention efforts.

Many countries lack surveys of UAPP amongst farmers and workers. Additionally, surveys on UAPP lack a standardized case-definition of acute poisoning. We recommend that investigators base the case definition on the IFSC classification and clearly report on the chosen population, latency and at-risk times. Future study directions on UAPP would include prospective cohort studies for chronic outcomes to better understand long term effects of acute poisoning. In the future, government and hospital reports should differentiate between farmers, workers, and children in reporting mechanisms to allow a better understanding of the extent of the problem in these population categories. Finally, improvements in data collection would allow for a regular and reliable monitoring of UAPP and support the implementation and evaluation of preventive public health policies.

Independent of improvements in data collection and the available database on UAPP, our review of the existing evidence is more than sufficient to warrant immediate action. The implementation of the international recommendations by the FAO Council to phase out HHPs, and Target A7 of the Global Framework on Chemicals to phase HHPs out by 2035, where the risks have not been managed, could significantly reduce the burden of UAPP. It should be emphasized that for pesticides posing a high risk, the International Code of Conduct on Pesticide Management recommends the end of their use through regulatory action, if less hazardous alternatives are available. In the European Union, where virtually all pesticides of high acute toxicity are banned, certification of adequate training of professional pesticide users including the periodic renewal of this certification is legally required. It would be urgent to establish appropriate nationwide systems of training and certification in countries where pesticides of high acute toxicity are still marketed. At the same time the availability of mitigating measures (e.g., personal protective equipment) proposed during such trainings needs to be ensured.

Preventive measures should particularly address pesticides listed by WHO as Class 1a (extremely hazardous) and Class 1b (highly hazardous) which should only be permitted for use by trained persons. Employers should be obliged to provide suitable personal protective equipment.

## Data Availability

The original contributions presented in the study are included in the article/Supplementary material, further inquiries can be directed to the corresponding author.

## Footnotes

1
https://pan-germany.org/download/rebuttal-by-the-authors-of-the-retraction-of-the-article-on-the-global-distribution-of-unintented-acute-pesticide-poisoning/

## Supplementary material

SUPPLEMENTARY TABLE S1 Extracted articles by study characteristics.

SUPPLEMENTARY TABLE S2 Summary of national estimates.

SUPPLEMENTARY MATERIAL S1 Protocol for a systematic review on the global distribution of acute unintentional pesticide poisoning.

SUPPLEMENTARY MATERIAL S2 PRISMA checklist.

SUPPLEMENTARY MATERIAL S3 FULL search strategy.

SUPPLEMENTARY MATERIAL S4 MS-Excel sheet for data extraction.

SUPPLEMENTARY MATERIAL S5 Countries by region and data sources.

SUPPLEMENTARY MATERIAL S6 WHO mortality data by country.

SUPPLEMENTARY MATERIAL S7 National syntheses of UAPP by country.
