# Supplementary material for: Worldwide unintentional acute pesticide poisonings: reassessing the occupational and non-occupational burden
Source: Front Public Health. 2026 Sep 2;14:1825899. doi: 10.3389/fpubh.2026.1825899 (PMC13578083; doi:10.3389/fpubh.2026.1825899)
Supplement: Supplementary file 1 [file Data_Sheet_1.PDF]

Protocol for a systematic review on the global distribution of acute unintentional pesticide poisoning.

According to: PRISMA-P (Preferred Reporting Items for Systematic review and Meta-Analysis Protocols) 2015 checklist *

Section and topic	Item No	Checklist item
**ADMINISTRATIVE INFORMATION**		
Title:		
Identification	1a	The global distribution of acute unintentional pesticide poisoning: protocol for a systematic review
Update	1b	Does not apply
Registration	2	This systematic review protocol was not registered.
Authors:		
Contact	3a	Wolfgang Boedeker, PAN Germany, boedeker@epicurus.de<br>Peter Clausing, PAN Germany, pcl@jpberlin.de<br>Emily Marquez, PAN North America, emily@panna.org<br>Meriel Watts, PAN Asia Pacific, meriel@merielwatts.net
Contributions	3b	This protocol was drafted by WB. All authors provided feedback, discussed, and finally agreed on the manuscript.
Amendments	4	In the event of protocol amendments, the date of each amendment will be accompanied by a description of the change and the rationale
Support:		
Sources	5a	This review has been commissioned by the Pesticide Action Network (PAN). PAN is a network of over 600 participating nongovernmental organizations, institutions and individuals in over 90 countries working to replace the use of hazardous pesticides with ecologically sound and socially just alternatives. PAN North America and PAN Asia Pacific support this review financially by assigning staff members (EM) and consultants (MW).
Sponsor	5b	Does not apply
Are Role of sponsor or funder	5c	Does not apply

**INTRODUCTION**		
Rationale	6	Human poisoning by pesticides has long been considered a severe public health problem. Although the issue is prevailing and addressed in recent policy papers of international institutions the still most cited figure on acute poisoning was published by the World Health Organization (WHO) as long ago as 1990. The WHO taskforce estimated that about one million unintentional pesticide poisonings occur annually leading to approximately 20,000 deaths worldwide. Authors pointed to a vast underreporting as most incidents occur in countries with no effective monitoring of poisonings. More recent figures are available for self-poisoning by pesticides which in a systematic review is estimated to amount to 109,700-167,800 deaths per year (Mew et al. 2017.) However, twenty-eight years after WHO first published its estimate very little has been done to provide an accurate up-to-date picture of unintentional pesticide poisoning. Still today, peer reviewed authoritative studies relay on outdated information and estimates derived from the 1980s.
Objectives	7	The objective of our study is to estimate the global number of acute pesticide poisoning based on rates published for defined populations or groups within countries or regions, with the exception of intentional self-harm.
**METHODS**		
Eligibility criteria	8	The term “pesticides” refers to chemicals mainly used in crop production, vector control, biosecurity operations, vertebrate pest control, and household pest and weed management, and includes e.g. insecticides, herbicides, rodenticides, fungicides, and fumigants. The FAO/WHO International Code of Conduct on Pesticide Management defines pesticides as follows: Pesticide means any substance, or mixture of substances of chemical or biological ingredients intended for repelling, destroying or controlling any pest, or regulating plant growth. Papers will be included when the term “pesticide” or its subgroups, such as organophosphate, are stated.<br>We will include studies on acute pesticide poisoning covering accidental, homicidal and malicious poisoning in the general population as well as work-related and occupational poisoning. Studies dealing exclusively with suicidal pesticide poisonings or intentional self-harm will be excluded. So will studies on long-term effects like cancer.<br>We will include articles published between 2006 and December 2018.
Information sources	9	The primary source of this review will be the electronic databases PUBMED, EMBASE and Web of Science. The search will be supplemented by inspecting bibliographic reference lists in identified papers. Additionally, the websites of WHO, FAO, UNE, UNEP will be searched.<br>Other data sources will include other publically-related material, media reports, government reports, Poison Centre data, UN databases and reports, and other relevant reports that provide a clear methodology.<br>The search was developed by the study group. We consulted an expert for bibliographic data bases and search machines who checked the approach and improved the syntax. Searches of data bases will be carried out by WB. Websites will be inspected by PC and MW. All authors will be engaged in hand searches from lists of references.

Search strategy	10	The search starts at the above mentioned electronic databases. We aim at broad search categories while resulting in a manageable number of hits. A first extensive PUBMED search was refined by restricting search terms after inspection for clearly ineligible hits. The following search strategy was finally drafted for PUBMED and will be adapted to the other search engines:

Step	search terms	hits
#7	#6 AND Humans[Filter]	1,028
#6,	#5 AND ("2000"[Date - Publication] : "2018"[Date - Publication])	1,408
#5	#4 AND (survey[tw] OR register[tw] OR inciden*[tw] OR prevalen*[tw] OR mortality[tw] OR morbidity[tw])	2,088
#4	#3 AND (poison*[tw] OR "health effects"[tw])	10,677
#3	#1 OR #2	124,856
#2	insecticides[tw] OR insecticide[tw] OR fungicides[tw] OR fungicide[tw] OR herbicides[tw] OR herbicide[tw] OR rodenticides[tw] OR rodenticide[tw]	89,801
#1	Pesticide[tw] OR pesticides[tw] OR "crop protection chemicals"[tw] OR agrochemicals[tw] OR agrochemical[tw]	54,771

Study records:		
Data management	11a	Search results will be collected and stored by the literature data management software Zotero (https://www.zotero.org/) which allows handling of references, abstracts, and full-texts including check for duplicates. The Zotero data base will be made available as a group version for all authors.
Selection process	11b	All references will first be screened by title and abstract for eligibility of studies. Papers which by their abstract appear to meet the eligibility criteria or do not offer sufficient information to decide will be obtained in full-text. Each full-text manuscript will then be screened by two reviewers independently for meeting the inclusion criteria. In case of disagreement between the reviewers a consensus will be sought.
Data collection process	11c	A form for the extraction of information will be developed and applied to all included studies by two reviewers independently. We aim at identifying studies which are covered by more than one article in order to avoid double counting. In case of disagreement between the reviewers a consensus will be sought. We do not aim at contacting authors of primary studies for details not given in publications.
Data items	12	The number and outcomes of pesticide poisoning, the size of the at-risk population, incidence/prevalence rates or proportions will be extracted along with study characteristics as type, year, country/region/district, poisoned persons, cause of poisonings (accidental, occupational), conditions of use and pesticides involved.
Outcomes and prioritization	13	Primary outcome will be acute non-fatal and fatal pesticide poisoning without intentional self-harm. We rely on the definitions of study authors and aim to report for the categories of poisoning (accidental, work-related/occupational, homicides/malicious) as well as for groups of pesticides (e.g. organophosphate insecticides) when stated. We aim at an

		incidence rate in defined at-risk populations for the most recent reported year but will report for the time span addressed in studies.
Risk of bias in individual studies	14	Risk-of-bias of studies will be assessed by an agreed form. The form will be developed by the study group according to the GRADE recommendation on study limitations in observational studies and will consider the "Proposed Reporting Checklist for Authors, Editors, and Reviewers of Meta-analyses of Observational Studies" of the MOOSE-Statement. The risk-of-bias assessment will inform the data synthesis especially when more than one study is available for the categories of reporting (see below).
Data synthesis	15a	We expect different types of studies in terms of the target population (country/regions/occupations), reported expositions (all pesticides/group of pesticide/active ingredients/) and study designs (registers/surveys).<br>Our objective will be addressed by two steps. First, we review and calculate country-wide incidence rates and second, we estimate global figures for regions comprising these countries (see below). The above mentioned study types will be addressed differently in data synthesis. We will categorize the results according to the study characteristics and report in accordance with the PRISMA statement with the study countries as primary report category.
	15b	With respect to the country specific incidence rates we will consider results from registers or surveys giving the number of pesticide poisoning per total or well defined population and time span. In case results are available only for subsets of the population (e.g. farmers) or for a group of pesticides (e.g. organophosphates) the figures will be limited to these subsets and serve as a lower limit of the overall country-wide pesticide poisoning. In case more than one study reports for the same population and year the results will be combined to an overall figure considering the assessment of homogeneity and risk-of-bias. Whenever results are aggregated we use an inverse-variance weighting method. Focus will be the number of pesticide poisonings and crude as well as age standardized incidence rates. Results from surveys with ill-defined populations or from hospitals with unclear catchment area will not inform the national incidence rates.<br>For the estimation of the number of world-wide pesticide poisoning we follow the approach by Mew et al. 2017 "The Global Burden of Fatal Self-Poisoning with Pesticides 2006-15: Systematic Review". *Journal of Affective Disorders* 219: 93–104". The country-wide numbers of pesticide poisoning and the incidence rates will however be grouped according to the UN regions: Arabic Region; Africa Region; Asia Region; Latin America and Caribbean Region; Western Europe and Others Group. Extrapolation is based on the regions share of the world population. As there will be data gaps we will provide an extrapolation range based on study based minimum and maximum numbers.
	15c	Additionally, we plan to base global extrapolations on pesticide use pattern. 'Conditions of use' information together with the amount of pesticides used, and agricultural employment data may give a more homogenous grouping of countries as a basis of extrapolation.
	15d	See 15a

Meta-bias(es)	16	Global figures on pesticide poisoning are challenged by overall bias sources. There will be an anticipated publication bias as poisonings are often studied by health service institutions not primarily interested in the scientific publication of results. Furthermore, register data may be available for health monitoring in some countries but will not get published or disseminated. We expect a vast underreporting especially in low-income countries due to lack of scientific resources. On the other hand, surveys might be carried out primarily in poisoning "hot spots" and may provide data not suitable for extrapolation. We will study meta-bias by identifying the distribution of studies across countries and within and by other study characteristics. We will provide Funnel plots where homogeneity allows comparison of endpoints.
Confidence in cumulative evidence	17	We will refrain from formal grading of our confidence in the results. However, we will report on prevailing study types and number of studies with respect to world-regions, countries, study characteristics and pesticide use pattern. Besides the number of acute pesticide poisoned (excluding intentional self-harm) we provide incidence rates to allow for extrapolation to other populations by readers.

** Shamseer L, Moher D, Clarke M, Ghersi D, Liberati A, Petticrew M, Shekelle P, Stewart L, PRISMA-P Group. Preferred reporting items for systematic review and meta-analysis protocols (PRISMA-P) 2015: elaboration and explanation. BMJ. 2015 Jan 2;349(jan02 1):g7647.*
