# Supplementary material for: Worldwide unintentional acute pesticide poisonings: reassessing the occupational and non-occupational burden
Source: Front Public Health. 2026 Sep 2;14:1825899. doi: 10.3389/fpubh.2026.1825899 (PMC13578083; doi:10.3389/fpubh.2026.1825899)
Supplement: Supplementary file 3 [file Data_Sheet_3.PDF]

UAPP Revisited, Supplement 3

Presentation of the full search strategies for all databases according to PRISMA 2020.

Automatic search

The orientating PubMed Search was refined by varying the search terms, term truncation and limiting to specific fields. The results were compared with respect to the mutual coverage and checked against articles known to be relevant for the review. Search terms from missed articles were added, but skipped when results were shifted to more toxicological, clinical, and treatment issues. So e.g. we deleted “health effects” which along with “intoxication” seems to add to the above mentioned shifting of focus. We also included “organosphos*” with almost no change. The search strategy was finally agreed on for PUBMED and was adapted to the other search engines.

Table 1 to Table 4 give the search terms and results for every database.

Table 1: Results of PUBMED search by 2023-11-23 for period 2019-2023

Step	Search terms	hits
**#7**	#6 AND Humans[Filter]	288
**#6**	#5 AND ("2019"[Date - Publication] : "2023"[Date - Publication])	506
**#5**	#4 AND (survey[tw] OR register[tw] OR inciden*[tw] OR prevalen*[tw] OR mortality[tw] OR morbidity[tw])	2,626
**#4**	#3 AND (poison*[tw] OR "health effects"[tw])	12,593
**#3**	#1 OR #2	169,800
**#2**	insecticides[tw] OR insecticide[tw] OR fungicides[tw] OR fungicide[tw] OR herbicides[tw] OR herbicide[tw] OR rodenticides[tw] OR rodenticide[tw]	117,883
**#1**	Pesticide[tw] OR pesticides[tw] OR "crop protection chemicals"[tw] OR agrochemicals[tw] OR agrochemical[tw]	78,839

The search was applied to the Web-of-Science (WOS) and EMBASE.

Table 2: Results of Web-of-Science search by 2023-11-21 for period 2019-2023

Step	search Term	hits
**#1**	TS=(Pesticide OR pesticides OR "crop protection chemicals" OR agrochemicals OR agrochemical)	45,437
**#2**	TS=(insecticides OR insecticide OR fungicides OR fungicide OR herbicides OR herbicide OR rodenticides OR rodenticide)	44,480

**#1 OR #2**	TS=(Pesticide OR pesticides OR "crop protection chemicals" OR agrochemicals OR agrochemical) OR TS=(insecticides OR insecticide OR fungicides OR fungicide OR herbicides OR herbicide OR rodenticides OR rodenticide)	76,420
**#4**	TS=(poison* OR "health effects")	39,698
**#3 AND #4**	(TS=(Pesticide OR pesticides OR "crop protection chemicals" OR agrochemicals OR agrochemical) OR TS=(insecticides OR insecticide OR fungicides OR fungicide OR herbicides OR herbicide OR rodenticides OR rodenticide)) AND TS=(poison* OR "health effects")	2,214
**#6**	TS=(survey OR register OR inciden* OR prevalen* OR mortality OR morbidity)	1,759,861
**#5 AND #6**	((TS=(Pesticide OR pesticides OR "crop protection chemicals" OR agrochemicals OR agrochemical) OR TS=(insecticides OR insecticide OR fungicides OR fungicide OR herbicides OR herbicide OR rodenticides OR rodenticide)) AND TS=(poison* OR "health effects")) AND TS=(survey OR register OR inciden* OR prevalen* OR mortality OR morbidity)	626

Table 3: Results of EMBASE search by 2023-11-23 for period 2019-2023

Step	search terms	hits
**1**	(pesticide* or "crop protection chemicals" or agrochemical*)	29,958
**2**	(insecticide* or fungicide* or herbicide* or rodenticide*)	26,132
**3**	1 or 2	47,545
**4**	(poison* or "health effects")	44,864
**5**	(survey or register or inciden* or prevalent* or mortality or morbidity)	1,801,502
**6**	3 and 4 and 5	835

Search results including abstracts were downloaded and stored by the literature data management software Zotero (https://www.zotero.org/) which allows handling of references, abstracts, and full texts including checking for duplicates. The Zotero data base was made available as a group version for all reviewers. Finally, 1,156 references (without duplicates) were retrieved by the automatic search.

Table 4: Combined search results transferred to Zotero data base

Source	References found (dates see above)	Imported to Zotero (by 2023-11-27) *
PubMed	288	287
EMBASE	835	831
WOS	626	626
WOS not PUBMED		433
EMBASE not WOS not PUBMED		436
combined search no duplicates		1,156

* deviations due to cross-references and duplicates

Hand search

Supplementing the automatic search all authors checked their own collections for eligible papers not identified so far. Further articles were identified during the selection and extraction step of the review by inspecting bibliographic reference lists of included papers and citation tracking. All supplemental papers are assessed the same way as those ones we found by the automatic search.
