# Supplementary material for: Worldwide unintentional acute pesticide poisonings: reassessing the occupational and non-occupational burden
Source: Front Public Health. 2026 Sep 2;14:1825899. doi: 10.3389/fpubh.2026.1825899 (PMC13578083; doi:10.3389/fpubh.2026.1825899)
Supplement: Supplementary file 4 [file Data_Sheet_7.pdf]

National syntheses of unintentional acute pesticide poisoning (UAPP) by country

Procedure 3
1. Albania..........4
2. Argentina..........5
3. Australia..........6
4. Bangladesh..........8
5. Bolivia..........11
6. Brazil..........12
7. Burkina Faso..........18
8. Cambodia..........20
9. Cameroon..........23
10. Canada..........25
11. Chile..........26
12. China..........28
13. Colombia..........32
14. Costa Rica..........34
15. Cote d'Ivoire..........36
16. Ecuador..........38
17. Egypt..........39
18. Ethiopia..........40
19. France..........43
20. Gambia..........44
21. Georgia..........45
22. Germany..........46
23. Ghana..........47
24. Hungary..........48
25. India..........49
26. Indonesia..........55
27. Iran..........58
28. Italy..........61
29. Jamaica..........62
30. Japan..........63
31. Kenya..........64
32. Kuwait..........66
33. Lao DPR..........67
34. Lebanon..........69
35. Malawi..........70
36. Malaysia..........71
37. Mexico..........73
38. Moldova..........75
39. Morocco..........76
40. Nepal..........78
41. Nigeria..........81
42. Pakistan..........83
43. Palestine..........86
44. Philippines..........88
45. Portugal..........90
46. Saudi Arabia..........91

47. Senegal 92
48. Serbia 93
49. South Africa 94
50. South Korea 96
51. Spain 99
52. Taiwan 100
53. Tanzania 102
54. Thailand 105
55. Turkey 108
56. Uganda 109
57. UK 111
58. USA 113
59. Venezuela 121
60. Vietnam 123
61. Zambia 125
62. Zimbabwe 126
References 127

Procedure

This chapter is a supplementary part of the manuscript "Worldwide unintentional acute pesticide poisoning revisited". It provides information on the synthesis of data for the 62 countries for which data were extracted from publications and used to arrive at national estimates of UAPP. These country synopses highlight specific limitations of the data used, the estimation approach, and specify which publications and data were used for national estimates.

For each country, all included papers were revisited and related to each other and the WHO mortality data. We guided the extrapolation as close as possible to the study population, so for example abstaining from an extrapolation to the whole population when the study base was farmers. Data on the national at-risk or country population were searched for via the internet if not reported in the extracted papers. We looked for population data most closely matching the studied population and study period. If data were not available from other sources, we used data from the World Bank (https://databank.worldbank.org/source/population-estimates-and-projections/Type/TABLE/preview/on#). World Bank provides figures on the overall population and children 0-14 years, as well as the percentage of employment in agriculture and the total employment in countries worldwide. Some studies reported on specific applicators (e.g. contract workers), farm-type (e.g. orchards), crops (e.g. tomatoes), or even pesticides (e.g. paraquat). The decision whether to consider those studies for national estimates was taken on a case-by-case basis, and dependent on the availability of other studies, how representative they could be considered to be, etc.

We calculated the "farmers/occupational" population by multiplying the share of agriculture by the total employment figures. Because "employment" is for some countries too narrow a definition as it might not include informal farming, estimations of cases are probably too low for some countries. If studies cover the same population/pesticides etc., those more recent or those with less risk-of-bias were preferred, for example those with representative samples and verified diagnoses or pesticides. We reported fatal and non-fatal cases on three types of populations: general (the "all" population category in the spreadsheet of the systematic review), farming/occupational (includes "farmers & workers", "farmers only" and "workers only") and children.

The included data covers the years 2006-2018 (first review period) and 2019-2023 (second review period). For WHO mortality data, we preferred data from the second period. However, if no recent data were available for countries which were included in the first review period, we carried forward the data. This was the case for three countries: Jamaica, Morocco, and Venezuela.

In general, the number of national cases of UAPP was based on the national figures, if provided by the publications, or was estimated by extrapolation of a prevalence ratio to the respective population. For example, if, based on average of surveys, 30 % of farmers are reported to suffer UAPP the total number of non-fatal cases would be derived by multiplying this ratio by the size of the respective population in the country. Country specific approaches are detailed below.

National estimates for nonfatal UAPP among the farming/occupational population are mainly based on publications reporting on surveys. For this population, we therefore provide a summary table of study characteristics for each country. The table includes the number of studies considered and the quality criteria, e.g. in terms of sampling and case definition. All appropriate studies for each country are combined in the table and the highest values for each quality criterion are reported.

1. Albania

Publications extracted for synthesis 2006-2018: 1

Publications extracted for synthesis 2019-2023: 0

Sulaj et al. 2015 (1)

- Aluminium phosphide poisonings only
- Period of study = 2009-2013
- Area of study = Tirana, the capital of Albania
- Mainly suicides
- Of 140 fatalities, 8 cases (6%) were not suicides
- The population figure of 773,843 was taken from Table 2 of the study, where the authors reported the population of Tirana

Population type	Year	Population	Fatal	Non-fatal	Total cases
General	2009-2013	773,843	8		8

Estimation of national figures

The study focused on poisonings and fatalities from a single pesticide, aluminum phosphide. As this pesticide is commonly available and comes in a tablet form, many of the cases were via ingestion and the great majority were due to suicides, so only the data on unintentional poisonings reported in this study can be used. The authors' focus was on the fatalities, and the non-fatal cases had no information on whether accidental poisonings were included.

The study also focused on a single university centre in the capital city of Tirana, Albania. The data were extracted, but Albania was not included in the estimate because the study did not represent the general use of, or exposure to, pesticides in for the following reasons: the data were from one city, for one pesticide that has very narrow and specific uses.

2. Argentina

Publications extracted for synthesis 2006-2018: 1

Publications extracted for synthesis 2019-2023: 0

Butinof et al. 2015 (2)

- Period of study = 2007-2010
- Agricultural workers, i.e. terrestrial pesticide applicators
- Area of study = Province of Córdoba
- Sample size = 880 male applicators with direct exposure to pesticides was identified for further analysis from a "universe" of 3,500 individuals
- Self-reported symptoms
- Morbidity = 47.4% (for highest prevalence). This was for "irritation", a term that – according to the authors – subsumed "irritation of skin, eyes, nausea, vomiting, and respiratory disorders"

WHO Mortality data: available from 2017-2021 with 2.4 annualized fatal cases of UAPP for the general population, including 0.2 cases for children and 0 for the farming/occupational population.

Estimation of national figures

For the farming/occupational population we used the results of Butinof et al. 2015, for an average population of 1,684,948.

Annual national estimate for Argentina

Population type	Year	Population	Fatal	Non-fatal	Total cases
General	2017-2021	44,932,707	2.4		2.4
Children	2017-2021	10,741,326	0.2		0.2
Farming/occupational	2007-2010,<br>2017-2021	1,684,948<br>1,487,664	0	798,665	798,665

National estimates based on studies with following study characteristics

Criteria	Farming/occupational population
Number of studies used for calculation	1
Review periods covered (first, second, both)	first
Sampling (representative, simple sample or unspecified)	representative
Diagnose (study scientists, self-reported)	self-reported
Latency period (24 h, 48 h, other, unspecified)	unspecified
At-risk time (annual, <1 y, other, ever, unspecified)	unspecified
No. of symptoms (>=1, >=2, unspecified)	unspecified

3. Australia

Publications extracted for synthesis 2006-2018: 1

Publications extracted for synthesis 2019-2023: 0

Ling et al 2018 (3)

- A retrospective case series of the Poison Centre for the State of Victoria
- Period of study = June 2005 - December 2013
- Occupational, but does not specify if it is farmers, workers, urban pest control operators or others using pesticides in an occupational setting
- Morbidity = 70 cases per year

WHO Mortality data: On average, 1.3 fatal pesticide poisonings per year were reported for the general population of Australia (2017-2022) and zero cases for children and the farming population respectively.

Estimation of national figures

The population of Victoria was estimated to be 6,566,200 on 31st March 2019, according to the Australia Bureau of Statistics (ABS), representing 38.5% of the total Australian population of 25,287,400. The ABS did not provide population figures for all years, while FAOSTAT did provide data for all years, but did not provide population figures for Victoria. In order to address these data gaps, the data sources were combined. Data for particular years available from both FAOSTAT and ABS were found to match.

ABS figures (5,642,873) were used to calculate the % of Australian population that lives in Victoria and this percentage was then applied to the FAOSTAT annual population for the relevant years to arrive at a projected annual reported occupational pesticide poisoning for the whole of Australia over the period 2005-2013.

Year	Population Australia (FAOSTAT)	Population Victoria (ABS)	Total cases Victoria
2005	20,178,540		
2006	20,526,303		
2007	20,916,340		
2008	21,332,282		
2009	21,750,851		
2010	22,154,679		
2011	22,538,001		
2012	22,903,948		
2013	23,254,913		
average	21,728,429	5,642,873	70

Annual national estimate for Australia

Population type	Year	Population	Fatal	Non-fatal	Total cases
General	2017-2022	25,371,812	1.3		1.3
Children	2017-2022	4,709,835	0		0
Farming/ occupational	2005-2013	349,697	0	270	270

National estimates based on studies with following study characteristics

Criteria	Farming/occupational population
Number of studies used for calculation	1
Review periods covered (first, second, both)	first
Sampling (representative, simple sample or unspecified)	simple sample
Diagnose (study scientists, self-reported)	study scientists
Latency period (24 h, 48 h, other, unspecified)	unspecified
At-risk time (annual, <1 y, other, ever, unspecified)	annual
No. of symptoms (>=1, >=2, unspecified)	unspecified

Limitations

There is only one publication available, for only one state. It provides occupational data but does not specify the population using pesticides in an occupational setting, i.e., farmers, workers, urban pest control operators and/or others. It reports only on cases reported to the Poisons Centre, so underreporting is highly likely.

4. Bangladesh

Publications extracted for synthesis 2006-2018: 6

Tomenson and Matthews 2009 (4)

- Period of study = 2006
- Representative sample of 258 farmers & workers
- Morbidity = 42.64% (110 cases in the past 12 months)
- Data ok to extrapolate

Dey 2010 (5)

- Period of study = presumed to be 2008
- Area of study = three sub-districts, not identified
- Sample = 180 vegetable farmers; representative sample, randomly selected from three different sites with high, medium, low cropping intensities
- Morbidity = 29.8% (for symptom with highest prevalence, 54 cases)
- Data ok to extrapolate

Miah et al. 2014 (6)

- Period of study = 2012
- Area of study = Bharella, Mokam and Rajapur unions of Burichong Upazila
- Sample = 120 male farmers; representative sample selected for high, medium, and low level of vegetable production
- Morbidity = 55% (for symptom with highest prevalence, 66 cases)
- Data ok to extrapolate

Akhter et al. 2016 (7)

- Period of study = 2016
- Area of study = Kaligonj and Jhenidah sadar upazilas in the District of Jhenidah
- Sample = 80 male farmers; representative sample, growing mainly vegetable crops
- Morbidity = 84% (for symptom with highest prevalence, 67 cases)
- Data ok to extrapolate

Akter et al. 2018 (8)

- Period of study = 2015-2016
- Area of study = Palbandha and Char Goalini unions in Islampur Upazila in the District of Jamalpur
- Sample = 101 male vegetable farmers; representative sample
- Morbidity = 60.4% (for symptom with highest prevalence, 61 cases)
- Data ok to extrapolate

Rengam et al. 2018 (9)

- Period of study = 2017
- Area of study = the five sub-districts of Satkhira district

- Sample = 534 farmers (77%) and workers, including 40 women, rice and vegetable growers
- Morbidity = 62% (for symptom with highest prevalence, 330 cases)
- Data ok to extrapolate

Publications extracted for synthesis 2019-2023: 2

Kobashi et al 2021 (10)

- Period of study = 2020-2021
- Area of study = Lohagara Upazila in Narail District
- Community-based interventional study in which a checklist for pesticide protective habits was distribute to the sample
- Farmers and workers, growing paddy, jute, wheat, mustard, betel leaf, and vegetables, while the main fruits are mango, jackfruit, litchi, banana, papaya, star apple, blackberry, coconut, betel nut, shaddock, and hog-plum
- Sample = 100 males
- Symptoms self-reported
- Morbidity = 14% (14 cases)
- Whilst use of protective clothing was measured before and after the intervention (average pesticide-protective behavioural score increased from 4.58 in the baseline survey to 8.11 in the follow-up), it appears symptoms were measured only after the intervention. It's likely that the reported morbidity is lower as a result of the intervention
- Data ok to extrapolate

Diyana et al 2022 (11)

- Period of study = 2021
- Area of study = Manikganj and Cumilla districts
- Farmers and workers, rice and vegetables, maize, jute
- Sample = 99, representative sample, 50% female
- Symptoms self-reported
- Morbidity = 21.21% (21 cases)
- Data ok to extrapolate

WHO Mortality data: no mortality data were available.

Estimation of national figures

We based our extrapolation on the average morbidity of all included studies.

Study	Study year	Sample	Morbidity (%)
Tomenson & Matthews 2009	2006	258	42.6
Dey 2010	2008	180	29.8
Miah et al. 2014	2012	120	55
Akhter et al. 2018	2015-16	101	60.4
Akter et al. 2016	2016	80	84
Rengam et al. 2018	2017	534	62
Kobashi et al. 2012	2020-21	100	14
Diyana et al. 20221	2021	99	21
	Average		*46*

Annual national estimate for Bangladesh

Population type	Years	Population	Fatal	Non-fatal	Total cases
Farming/occupational	2006-21	28,559,345		13,137,299	13,137,299

National estimates based on studies with following study characteristics

Criteria	Farming/occupational population
Number of studies used for calculation	8
Review periods covered (first, second, both)	both
Sampling (representative, simple sample or unspecified)	representative
Diagnose (study scientists, self-reported)	self-reported
Latency period (24 h, 48 h, other, unspecified)	24 h
At-risk time (annual, <1 y, other, ever, unspecified)	annual
No. of symptoms (>=1, >=2, unspecified)	unspecified

5. Bolivia

Publications extracted for synthesis 2006-2018: 1

Publications extracted for synthesis 2019-2023: 0

Jørs et al. 2014 (12)

- Period of study = 2002, 2004, 2009
- Area of study = four municipalities within the La Paz County
- Analysed the effect of "Farmer Field Schools" (FFS) on the absence of self-reported pesticide intoxication symptoms in 22 FFS-farmers and 47 "neighbor" farmers who had no FFS-participation
- Prevalence of self-reported symptoms could be inferred from the rates of absence of symptoms in Table 2: 2009; of the neighbor farmers only 45.7 % "self-reported symptoms after spraying last year" (21 of 46 cases)

WHO Mortality data: no mortality data were available.

Annual National estimate for Bolivia

Population type	Year	Population	Fatal	Non-fatal	Total cases
Farming/occupational	2009	1,333,541		608,790	608,790

National estimates based on studies with following study characteristics

Criteria	Farming/occupational population
Number of studies used for calculation	1
Review periods covered (first, second, both)	first
Sampling (representative, simple sample or unspecified)	simple sample
Diagnose (study scientists, self-reported)	self-reported
Latency period (24 h, 48 h, other, unspecified)	unspecified
At-risk time (annual, <1 y, other, ever, unspecified)	annual
No. of symptoms (>=1, >=2, unspecified)	unspecified

6. Brazil

Publications extracted for synthesis 2006-2018: 7

Faria et al. 2009 (13)

- Period of study = 2006
- Area of study = municipality of Bento-Gonçalves (Southern Brazil)
- Sample = 290 fruit farmers in 2006
- Morbidity = 3.8% during a 12 month period
- Symptoms occurring within 48 hrs of exposure and over the 10 days before the interview
- Data ok to extrapolate

Santana et al. 2013 (14)

- Period of study = 2000-2009
- Farmers and workers
- National mortality data from the *Sistema de Informação de Mortalidade* (SIM) were used and the extracted mortality data classified as X48 according to ICD-10 for farm workers
- Mortality rates (per 100,000 persons) of 0.37 for 2006 to 2007 and 0.39 for 2008 to 2009
- Data on fatalities were not used because WHO mortality data (2017-2021) were available

de Albuquerque et al. 2015 (15)

- Period of study = 2008-2012
- Area of study = state of Pernambuco
- General population at the district level
- Cross-sectional study that compared the contribution of three different databases (SINAN, CEATOX and SIM) to the characterization of pesticide poisoning
- Total number of fatal cases for the five-year period which was highest in SIM (n=552), but this number includes categories other than X48 (e.g. X68 [self-poisoning] etc.)
- 161 fatal UAPP cases reported, no population data given
- Data on fatalities were not used because WHO mortality data (2017-2021) were available

dos Santos Cargnin et al. (2017) (16)

- Period of study = 2012
- Area of study = state of Rio Grande do Sul
- Sample = 100 male tobacco growers (97 of whom confirmed using pesticides)
- Morbidity = 20.6% (20 cases)
- Inconsistencies and limitations: 14 cases of vomiting in the text, described as 22.6%; interviews were carried out in January 2012, the authors claimed to have made a survey in 2012-2013
- Data ok to extrapolate

De Souza Campos et al. (2017) (17)

- Period of study = October 2013 to September 2014 (12 months)

- Data from a regional database (CIATOX) for the region of Campinas within the state of São Paulo) encompassing 90 municipalities and an estimated population of 6.5 million inhabitants, reporting on "legally and illegally commercialized household sanitizers"
- Children under 7 yrs
- 21 cases, no population data given
- Data were not used for an extrapolation because the population size of children under 7 years of the Campinas region is unknown

Lermen et al. (2018) (18)

- Period of study assumed to be 2014/2015 because of the ethics protocol number (2 072.009.2014)
- Area of study = state of Rio Grande do Sul
- Sample = 73 citrus growers (61.6% male); non-exposed control group of 30 persons
- Morbidity = 35% for most prevalent symptom (25 cases)
- Data ok to extrapolate

Magalhães and Caldas 2018 (19)

- Period of study = 2009-2013
- General population at national level
- Compared for fatal poisonings federal district data of 4 different databases and included national data from the *Sistema de Informação de Mortalidade* (SIM). SIM data which employed the ICD-10 classification were used here
- 246 fatal UAPP cases (X48)
- Data on fatalities were not used because WHO mortality data (2017-2021) were available

Publications extracted for synthesis 2019-2023: 11

Dalbó et al. 2019 (20)

- Period of study = 2012-2013 (according to information provided by lead author)
- Area of study = Mata Fria community in the state of Espirito Santo
- Sample= 142 farmers, simple sample, 71.8% male
- Survey with self-reported poisonings
- Morbidity = 66.2% (94 cases)
- Data ok to extrapolate

Silva et al. 2019 (21)

- Period of study = January to March 2013
- Area of study = 6 municipalities in the state of Mato Grosso
- Sample = 753 inhabitants, 293 of them "working in agriculture or similar activities", 59.2% male
- A "convenience" sample was used
- Morbidity = 28.0% (82 cases), farming/occupational

- At-risk time: 1 month: "The dependent variable for this study was having reported any acute intoxication symptom in the month prior to the interview and which had been diagnosed by a doctor."
- The authors provide data on “self-reported intoxications diagnosed by a doctor” and state in their Methods section: “We did not ask whether those symptoms were related to pesticides or not”. This contradiction was resolved because the overall prevalence refers only to medically confirmed UAPP, but the individuals were not interviewed further by the study scientists. Prevalence was based on the number of cases found in the agricultural/occupational population
- Data ok to extrapolate

Bortolotto et al. 2020 (22)

- Period of study = January 2016-June 2016
- Area of study = municipality Pelota in the state of Rio Grande do Sul
- Sample = 1,518 farmers and workers; representative, 51.7% female
- Morbidity = 5.9% (89 cases)
- ‘Ever’ at-risk timeframe
- Data not used for extrapolation because of ‘ever’ timeframe

de Araujo Nascimento et al. 2020 (23)

- Period of study = 2007-2017
- Study area = national
- Sample: DataSUS, Ministry of Health database on “agricultural poisoning”
- Morbidity = 0.0011% (estimated from Figure 1 – 2,290 annual unintended poisonings, national population 199,838,180)
- Data not included for extrapolation because of unclear reference population and coverage

Silvério et al. 2020 (24)

- Period of study = June 2014-June 2015
- Area of study = 26 municipalities in the state of Minas Gerais
- Sample = 1,027 rural workers; representative
- Morbidity = 17.3% (178 cases)
- ‘Ever’ at-risk prevalence
- Data not used for extrapolation because of ‘ever’ prevalence

Xavier et al. 2020 (25)

- Period of study = January 2018-March 2018
- Area of study = South Region of Brazil (no further specification)
- Children and young adults (10-24 years), resident in rural areas, working with the family in the rural environment with or without periodic remuneration
- Sample =211; representative, 55.9% female
- Morbidity = 2.6% (5.5 cases), based on a survey on occurrence of and reason for work accidents
- At-risk time: 3 months, “exposed to risks and accidents … during the study period”

- Data were not used for extrapolation because the study includes young adults “working with the family,” which might result in other pesticide exposure than occupational activities. We could not retrieve data on the size of the agricultural population in this age group

dos Santos et al. 2021 (26)

- Period of study = 2012-2016
- Area of study = state of Espírito Santo
- Sample = cases notified to the Poison Center and recorded in their TOXCEN database, no selection
- Morbidity = 9.5 per 100,000 (332 cases/year, population 3,514,952)
- Authors states that, in Brazil reports to the poison centres are not mandatory. In addition, “the TOXCEN service is located in an urban area within the capital of the State (Vitória) and individuals living in rural areas where agricultural activity is more prevalent, may not know of its existence”. Thus, both factors are likely to contribute to an underestimation of the true prevalence
- Data not included for extrapolation because of unclear reference population and coverage

Fassa et al. 2021 (27)

- Period of study = January-March 2011
- Area of study = municipality of São Lorenço do Sul in the south of Brazil
- Sample = 99 child workers (young people); representative
- Morbidity = 3.0%
- ‘Ever’ at-risk timeframe
- Data not used for extrapolation because of ‘ever’ timeframe

Carvalho et al 2022 (28)

- Period of study = 2007-2016
- Area of study = state of Espírito Santo
- “In this study, we used records of notified poisoning cases in the state, made available by Espírito Santos State Department of Health (SESA/ ES), through its Research Analysis Committee within the State Health Network. Data were generated through exogenous poisoning investigation forms input to SINAN by Municipal Health Departments”
- Sample = 3,514,952 (population of state in 2010)
- Morbidity = 0.00367% (129 nonfatal, unintended poisonings per year), 1 fatality per year
- There is underreporting due to an incomplete data base in this study: “When comparing this investigation data with the number of deaths registered on the Mortality Information System (SIM), it can be seen significant underreporting in the SINAN data…”
- Data not included for extrapolation because of unclear reference population and coverage

de Melo et al. 2022 (29)

- Period of study = 2007-2015
- Area of study = municipality Arapicara in state of Alagoas
- Children (0-9)
- Sample = 3,405 (no selection)

- Morbidity = 0.18% (6 cases)
- Other age-groups reported but including suicides
- Records were extracted from the Notifiable Diseases Information System (Sistema de Informação de Agravos de Notificação – SINAN) of the Brazilian Health Informatics Department of the Ministry of Health (DATASUS)
- Data not included for extrapolation because of unclear reference population and coverage

Faria et al. 2023 (30)

- Period of study = not stated
- Area of study = municipality São Lourenço do Sul in state of Rio Grande do Sul
- "This article presents a subsample analysis of family tobacco growers aged 18 years or over, who applied pesticides…"
- Sample = 492; representative sample, 95.2% male
- Morbidity = 10.2% (51 cases)
- At-risk time = 15 days ("25 symptoms common to pesticide poisonings that occurred in the last 15 days were investigated")
- Data ok to extrapolate

Estimation of national figures

For children nonfatal UAPP, we could not estimate based on data from De Souza Campos et al. 2017 because the population data were not available for the Campinas region.

For fatal UAPP, we did not use the data from the studies by Magalhães and Caldas 2018, Santana et al. 2013, de Albuquerque et al. 2015, because the more recent and complete WHO mortality data from 2017-2021 were available.

For the farming/occupational UAPP estimate, we excluded Carvalho et al 2022, De Araujo Nascimento et al. 2020, de Melo et al 2022, and dos Santos et al. 2021, as all are based on registers with an unclear reference population and coverage. We did not use the studies of Bortolotto et al. 2020, Fassa et al. 2021, and Silvério et al. 2020 for the estimate, due to the 'ever' prevalence.

We did not include non-fatal UAPP for child agricultural workers in the farming/occupational estimate, because the study from Xavier et al. 2020 includes young adults "working with the family" which might result in other pesticide exposure than occupational activities. Furthermore, we could not retrieve data on the child agricultural worker population.

For non-fatal UAPP in the farming/occupational population, the average prevalence from the six included studies was 25.9 %, so 10,938,464 * 0.26 = 2,844,001 cases:

Study	Study Year	Sample	Morbidity (%)
dos Santos Cargnin et al. 2017	2012	100	20.6
Faria et al. 2009	2006	290	3.8
Lermen et al. 2018	2014- 2015?	73	35

Study	Study Year	Sample	Morbidity (%)
dos Santos Cargnin et al. 2017	2012	100	20.6
Dalbó et al. 2019	2012-2013	142	66.2
Faria et al. 2023	not stated, assumed 2020	492	10.2
Silva et al. 2019	2013	753	28
Average			*25.9*

Annual national estimate for Brazil

**Population Type**	Year	**Population** *	**Fatal**	**Non-fatal**	**Total cases**
General	2017-2021	211,595,391	39		39
Children	2017-2021	44,725,859	3		3
Farming/occupational	2017-2021	9,672,899	5		
	2006,2012-2015, 2020	10,938,464		2,844,001	2,844,006

*average, World Bank data

National estimates based on studies with following study characteristics

**Criteria**	**Farming/occupational population**
Number of studies used for calculation	6
Review periods covered (first, second, both)	both
Sampling (representative, simple sample or unspecified)	representative
Diagnose (study scientists, self-reported)	study scientist
Latency period (24 h, 48 h, other, unspecified)	48 h
At-risk time (annual, <1 y, other, ever, unspecified)	annual
No. of symptoms (>=1, >=2, unspecified)	>=2

7. Burkina Faso

Publications extracted for synthesis 2006-2018: 2

Publications extracted for synthesis 2019-2023: 0

Toe et al. 2012 (31)

- Period of study = July-August 2009
- Area of study = Sahelian region
- Sample =112, male workers using insecticides
- Self-reported symptoms
- Morbidity = 85% (highest prevalence symptom, 95 cases)
- Data ok to extrapolate

Toe et al. 2013 (32)

- Period of study =June-July 2010
- Area of study = 3 major cotton growing areas: Hauts-Bassins, Cascades, and Boucle de Mouhoun
- Farmers
- Sample =650, representative
- Self-reported symptoms
- Morbidity = 82.66% (at least one symptom during or just after spraying, 537 cases), 98.3% male
- Data ok to extrapolate

The average prevalence, based on the most prevalent symptoms of pesticide poisoning reported in the two studies was 83.83%.

WHO Mortality data: no mortality data were available.

Estimation of national figures

Burkina Faso agricultural employment data are from the World Bank table: 3,556,616; so: 0.8383 * 3,556,616 = 2,981,511

National estimate for Burkina Faso

**Population type**	Year	**Population**	**Fatal**	**Non-fatal**	**Total cases**
Farming/occupational	2009-2010	3,556,616		2,981,511	2,981,511

National estimates based on studies with following study characteristics

**Criteria**	**Farming/occupational population**
Number of studies used for calculation	2
Review periods covered (first, second, both)	first
Sampling (representative, simple sample or unspecified)	representative
Diagnose (study scientists, self-reported)	self-reported
Latency period (24 h, 48 h, other, unspecified)	other
At-risk time (annual, <1 y, other, ever, unspecified)	unspecified
No. of symptoms (>=1, >=2, unspecified)	unspecified

8. Cambodia

Publications extracted for synthesis 2006-2018: 3

Publications extracted for synthesis 2019-2023: 0

Jensen et al. 2011 (33)

- Period of study = not specified, assumed to be >2006
- Area of study = villages of Thnout Chrum and Kba Tumnub at Boeung Cheung Ek Lake, Phnom Penh
- Sample = 89; representative sample, of aquatic farmers cultivating water spinach, grown as a "floating" crop; this sample was all the farmers from the villages
- Morbidity = 88% (78 cases), symptoms of UAPP in relation to spraying in the preceding month
- Data ok to extrapolate to other aquatic farmers; it is unknown whether it is representative of terrestrial farmers

FAO 2013 (34)

- Period of study = 2008 and 2010
- A complex study involving an FAO training programme. The report identified impacts of the IPM training, including comparing UAPP after the training in IPM (2010) with a baseline study before the training (2008), and also involved control groups of farmers not exposed to IPM training as well as those exposed to it indirectly. Only farmers for whom both baseline and impact data were available were included in the analysis, hence the sample size was 250 (90 +74 +86). Only the 2008 data could be extracted because of incomplete data from 2010
- The report states that in addition to the 58,716 farmers directly trained in FAO-supported Farmer Field Schools (FFS), thousands of additional farmers have benefited from FFS, so the results are relevant for a significant portion of Cambodian farmers and are ok to extrapolate
- Area of study = six FAO project and four control villages in two provinces (Battamabang and Prey Veng) on opposite sides of the country, both major rice and vegetable growing areas
- Sample = 250 respondents (127 respondents were interviewed twice)
- Paper states usually only serious pesticide poisoning cases or suicide attempts are reported
- Baseline study: in Battambang, 67% of farmers reported experiencing signs and symptoms, while in Prey Veng this figure was 73%
- The 2010 study shows a 13% decrease in UAPP in Battambang and a 17% decrease in Prey Veng as compared with the controls but does not give the actual data
- The UAPP has been averaged across both provinces, all groups
- Morbidity = 70% (210 cases)
- Data ok to extrapolate

Schreinemachers et al. 2017 (35)

- Period of study = 2015
- Area of study = 2 provinces, 6 districts, 12 subdistricts/communes, 22 villages, 300 households
- Sample = 300; representative sample, from main production area and main production season for farmers growing yard-long bean and leaf mustard, 29% female

- Morbidity = 54% (162 cases), for highest symptom incidence
- Authors noted that across three SE Asian countries pesticide use was 42% less when a woman was in charge of pest management
- Data ok to extrapolate

WHO Mortality data: no mortality data were available.

Estimation of national figures

The 3 studies cover aquatic vegetable growing, terrestrial vegetable growing and rice. There are 2 options:

i) Average all results across all crops, which results in 70.67%.

ii) Omit the aquatic vegetable growing because it is a specific situation, is earlier and a much smaller sample size. It is not known how big a portion of Cambodian agriculture involves aquatic spinach growing (FAO does not include it in crop data), although it is reported to be a major crop for Phnom Penh (Holm et al 2010)*. However, this cultivation is fed by both domestic and industrial sewage, and it is possible that some of the health impacts experienced by farmers arise from the contaminated water sources.

Option ii) was used for the estimate, however, the impacts of pesticide use on UAPP in aquatic vegetable growing should be noted.

Study	Study Year	Sample	Morbidity (%)
FAO 2013	2008, 2010	250	70
Schreinemachers et al. 2017	2015	300	54
Average (Option ii)			*62*

Annual national estimate for Cambodia

Population type	Year	Population	Fatal	Non-fatal	Total cases
Farming/occupational	2008-15	3,667,204		2,273,666	2,273,666

National estimates based on studies with following study characteristics

Criteria	Farming/occupational population
Number of studies used for calculation	2
Review periods covered (first, second, both)	first
Sampling (representative, simple sample or unspecified)	representative sample

Diagnose (study scientists, self-reported)	self-reported
Latency period (24 h, 48 h, other, unspecified)	unspecified
At-risk time (annual, <1 y, other, ever, unspecified)	unspecified
No. of symptoms (>=1, >=2, unspecified)	unspecified

* Holm PE, Marcussen H, Dalsgaard A, 2010. Fate and risks of potentially toxic elements in wastewater-fed food production systems— the examples of Cambodia and Vietnam. Irrig Drainage Syst. 2010 24:127–142 DOI 10.1007/s10795-009-9086-6.

9. Cameroon

Publications extracted for synthesis 2006-2018: 5

Publications extracted for synthesis 2019-2023: 0

Tomenson and Matthews 2009 (4)

- Period of study = 2006
- A survey of pesticide applicators and farmers commissioned by Syngenta Crop Protection among 2,431 users in eight countries
- Sample =261, representative
- Self-reported symptoms
- Morbidity = 59% (154 cases, reporting minor-to-serious incidents in the previous year)
- Data ok to extrapolate

Tandi et al. 2014 (36)

- Period of study = September 2012-November 2013
- Area of study = Buea municipality, SW region of Cameroon
- Sample = 93, tomato farmers, 96.7% male
- Morbidity = 84.9% (79 cases) reporting weakness as most prevalent symptom after spraying
- Data ok to extrapolate

Assokeng et al. 2017 (37)

- Period of study = April 10-August 15, 2013
- Area of study = Ngaoundere, a northern city of Cameroon
- Sample =60; representative, market gardeners (farmers)
- Self-reported symptoms
- Morbidity = 30% (18 cases, reporting most prevalent symptom of headache when handling pesticides)
- Data ok to extrapolate

Pouokam et al. 2017 (38)

- Period of study = 2016
- Area of study = 24 villages from five sites, representing the most important agricultural production areas of the five agroecological zones of Cameroon
- Sample = 395; representative, from farmers' groups
- Morbidity = 40.3% (159 cases)
- 'Ever' experienced poisoning
- This study was excluded from the extrapolation because of 'ever' prevalence

Achancho and Nsobinenyui 2019 (39)

- Period of study = October to December 2017
- Area of study = Munyenge, SW Cameroon
- Sample = 296; farmers, 97% male
- Self-reported symptoms

- Morbidity = 21% (62 of 296 reporting the most prevalent symptom of headache after spraying)
- Data ok to extrapolate

WHO Mortality data: no mortality data were available

Estimation of national figures

World Bank data for the population employed in agriculture were available for Cameroon through 2016. The population data from the years that data were collected in the studies was averaged. Pouokam et al 2017 was excluded from the estimation because 'ever' prevalence was reported.

Study	Year	Population	Morbidity (%)
Achancho et al.	2016	6,055,412	21
Assokeng et al.	2013	5,661,619	30
Tandi et al.	2012-2013	2012: 5,571,169<br>2013: 5,661,619	84.9
Tomenson & Matthews	2006	4,788,083	59.0
average			*49%*

National estimate for Cameroon

Population type	Year	Population	Fatal	Non-fatal	Total cases
Farming/occupational	2006, 2012-2013, 2016	5,519,071		2,704,345	2,704,345

National estimates based on studies with following study characteristics

Criteria	Farming/occupational population
Number of studies used for calculation	4
Review periods covered (first, second, both)	first
Sampling (representative, simple sample or unspecified)	representative sample
Diagnose (study scientists, self-reported)	self-reported
Latency period (24 h, 48 h, other, unspecified)	other
At-risk time (annual, <1 y, other, ever, unspecified)	annual
No. of symptoms (>=1, >=2, unspecified)	unspecified

10. Canada

Publications extracted for synthesis 2006-2018: 1

Publications extracted for synthesis 2019-2023: 0

Boyd 2007 (40)

- Period of study = 2006
- Reported unintentional poisonings for each province
- Morbidity = 0.018%
- No mortality data
- Because children under age 6 represented 46.5% of the reported poisonings, we calculated the statistic for children under 6 for the national estimates

WHO Mortality data: Three deaths over a 6-year period, resulting in 0.5 deaths per year from 2017-2022. No fatal UAPP was reported for children and the farming population.

Estimation of national figures

The calculation for the general population 18 per 100,000:

General population 2006: 0.00018* 32,570,505= 5,862

Children under age 6: 0.465 * 5,862 = 2,725.83

National estimates for Canada

Population type	Year	Population	Fatal	Non-fatal	Total cases
General	2006	32,570,505		5,862	
	2017- 2022	37,729,186	0.5		
General, years combined	2006, 2017-2022	34,820,511	0.5	5,862	5862.5
Farming/occupational	2017-2022	299,303	0		0
Children	2006 (< 15 y)	5,812,994	0		0
	2006 (< 6 y)	2,084,512		2725.8	2725.8
	2017-2022	5,998,933	0	0	0
Children, years combined	2006, 2017-2022		0	2725.8	2725.8

11. Chile

Publications extracted for synthesis 2006-2018: 2

Publications extracted for synthesis 2019-2023: 0

Ramirez-Santana et al. 2014 (41)

- Period of study = 3 years, January 2009 to December 2011
- Cases reported at the Ministry of Health from the Coquimbo area
- Sample = 45,000, population of Coquimbo agricultural workers, 52.6% males.
- Morbidity = 0.13% of agricultural workers were reported poisoned during that period (57 cases)

Munoz-Quezada et al. 2017 (42)

- Period of study not clear but assumed to be 2013-2016; 2014 was mentioned in association with knowledge of what pesticides were used in 2014
- Agricultural and non-agricultural workers from Maule, Chile, a large rural population with high rates of organophosphate (OP) pesticide poisoning; only agricultural worker data were extracted
- Organophosphate use
- Sample = 114, representative, 65% female
- Self-reported from a survey
- The authors also referenced a 2014 study that they had done, presumably with the same population of workers
- Morbidity = 35% (40 cases) poisoned by OP use (based on most prevalent symptom, headache)
- Data ok to extrapolate

WHO Mortality data: Reported as follows for 2017-2021, the total fatalities were 36, with an average of 7.2 fatalities per year from the general adult population and zero fatalities for children and the farming population.

Estimation of national figures

World Bank data on the population of farmers and workers from the years 2009-2011 and 2013-2016 (assumption for the years covered by the Munoz-Quezada study, see above) were used for this estimate. For the farming population, an average prevalence of 17.63% pesticide poisoning was used for the estimate.

Annual national estimates for Chile

Population type	Year	Population	Fatal	Non-fatal	Total cases
General	2017-2021	18,703,171	7.2		7.2
Farming/occupational	2009-2011, 2013-2016	784,044		138,226	138,226

National estimates based on studies with following study characteristics

Criteria	Farming/occupational population
Number of studies used for calculation	2
Review periods covered (first, second, both)	first
Sampling (representative, simple sample or unspecified)	representative
Diagnose (study scientists, self-reported)	self-reported
Latency period (24 h, 48 h, other, unspecified)	unspecified
At-risk time (annual, <1 y, other, ever, unspecified)	annual
No. of symptoms (>=1, >=2, unspecified)	>=2

12. China

Publications extracted for synthesis 2006-2018: 6

Zhang et al. 2011 (43)

- Period of study = 2009
- Area of study = Jiangsu province
- Sample = 910 farmers and workers from 2 villages; representative, 1 low- (Subei, 53.1% females) and 1 high-income (Sunan, 80% males) who had come into contact with pesticides while performing agricultural work in the 12 months preceding the study
- Symptoms self-reported during spraying or within 24 hr of pesticide exposure
- The authors required 2 or more symptoms to be indicative (8% morbidity); the authors had anticipated 10% was the likely poisoning rate
- Morbidity = 13.63% (124 cases) for any single symptom within 24 hrs of applying pesticides
- Data ok to extrapolate

Zhang et al. 2013 (44)

- Period of study = 2000-2010
- Area of study = Heilongjiang province
- Homicide = 4 cases per year
- Data source is the Forensic Science and Technology Division (FSTD) of the Public Security Bureau, which investigates toxic substances in crimes and cases of poisoning or death in which the cause is unclear
- Data not used in extrapolations because of unclear catchment area and at-risk population

Zhang et al. 2013b (45)

- Period of study = 2006-2010
- Area of study = Zhejiang province
- Data obtained from the Occupational Disease Surveillance and Reporting Systems, which covers hospitals, community healthcare clinics, rural clinics
- Morbidity = 478.4 (accidental) + 804.2 (occupational) cases per year = 1282.6
- Mortality =11.2 cases (accidental) + 5.4 (occupational) per year = 16.6
- Data ok to extrapolate for the general population

Zhang et al. 2016 (46)

- Period of study = 2009-2011
- Area of study = Jiangsu province
- Study reports “figures suggest 53,300-123,000 Chinese people are poisoned every year”
- Sample = 1490, representative sample of workers
- Morbidity = 8.12% (121 cases), experienced symptoms in last 12 months
- Data ok to extrapolate

Wang et al. 2017 (47)

- Period of study = 2006-2015

- Are of study = Jiangsu province – the pesticide poisoning data were acquired by means of a routine surveillance system by the Jiangsu Centre for Disease Prevention and Control
- Morbidity = 1325 cases; 401 accidental and 924 occupational cases)
- Mortality = 12 cases; 8 accidental, 4 occupational
- Data ok to extrapolate for general population

Wang et al. 2018 (48)

- Period of study = 2006-2017
- Area of study = Jiangsu province, children only, cases reported to Jiangsu Centre for Disease Prevention and Control
- At the age of 7, oral intake of pesticides to commit suicide began to appear, and it increased significantly after the age of 10
- Morbidity = 0.00049433% (55 cases UAPP)
- Data ok to extrapolate for children

Publications extracted for synthesis 2019-2023: 1

Wang et al 2019 (49)

- Period of study = 2006-2018
- Area of study = Jiangsu Province - no information on population size of Jiangsu over the period 2006-2018
- Data was collected from the pesticide poisoning report card established by the health hazard detection information system
- Farmers and workers in agriculture and forestry
- 607 non-fatal cases recorded
- Data were not included in the extrapolation due to a lack of information on the at-risk-population

WHO Mortality data: no mortality data were available.

Estimation of national figures

General population

We based the extrapolation for the general population on Wang et al. 2017 and Zhang et al. 2013 (b) and used the population share of the respective regions. The following population figures were obtained from Wikipedia:[1]

	Population	% of China's population
China	1,339,724,852	100%
Jiangsu	78,659,903	5.9 %
Zhejiang	54,426,891	4.1 %

[1] https://en.wikipedia.org/wiki/List_of_Chinese_administrative_divisions_by_population

Study	Region	Nonfatal	Fatal	Estimated nonfatal	Estimated fatal
Wang et al. 2017	Jiangsu	401	8	6,796.6	135.6
Zhang et al 2013(b)	Zhejiang	478.4	11.2	11,668.3	273.2
Average				*9,232.5*	*204.4*

Farming/occupational estimate

For the farming population, we based the extrapolation for the farming/occupational population on the surveys Zhang et al 2011 and 2016. We did not include data from Wang et al. 2017 and Zhang et al. 2013(b) because the at-risk populations were poorly defined.

Study	Study year	Sample	Morbidity (%)
Zhang et al 2011	2009	910	13.63
Zhang et al 2016	2009-2011	1,490	8.12
Average			*10.88*

For children, we used the study of Wang et al. 2018 for the Jiangsu region.

Annual national estimates for China

Population type	Year	Population	Fatal	Non-fatal	Total cases
General	2013,2017	1,376,797,500	204.4	9,232.5	9,437
Children	2006-2017	245,346,659		932	932
Farming/occupational	2011,2016	198,748,479		21,623,835	21,623,835

National estimates based on studies with following study characteristics

Criteria	Farming/occupational population
Number of studies used for calculation	2
Review periods covered (first, second, both)	first
Sampling (representative, simple sample or unspecified)	representative sample
Diagnose (study scientists, self-reported)	self-reported
Latency period (24 h, 48 h, other, unspecified)	24 h
At-risk time (annual, <1 y, other, ever, unspecified)	annual
No. of symptoms (>=1, >=2, unspecified)	>=2

13. Colombia

Publications extracted for synthesis 2006-2018: 4

Publications extracted for synthesis 2019-2023: 0

Tomenson and Mathews 2009 (4)

- Period of study = 2005-2006
- Surveyed pesticide applicators and farmers, commissioned by Syngenta Crop Protection among 2,431 users in eight countries
- Sample = 251, representative
- Self-reported symptoms
- Morbidity = 63.4% (159 cases, reporting minor-to-serious incidents)

Uribe et al. 2012 (50)

- Period of study = 2009-2010
- Area of study = la Merced-Caldas
- Sample = 132; group of workers engaged in tomato planting who had used carbamates, organophosphate and organochlorine pesticides in the last 6 months, 91% males
- Morbidity = 56% (74 cases, reporting having presented some symptoms at the time they were using pesticides)

Chaparro-Narvaez and Castaneda-Orjuela 2015 (51)

- Period of study = 1998-2011
- Only fatal pesticide poisonings. National registry of pesticides reports fatalities from pesticide exposure for homicides and accidental poisonings (classified by ICD-10 codes)
- Data were not used because the WHO mortality data were more recent

Varona et al. 2016 (52)

- Period of study = 2014-2015
- Area of study = Guamo-Espinal area in Tolima, Colombia
- Sample = 339; survey of farmers and workers working in rice cultivation, males
- Self-reported symptoms after direct contact with pesticides
- Morbidity = 86.1% (292 cases)

Estimation of national figures

For the farming/occupational population, we averaged the prevalence of UAPP of the included studies to arrive at a mean of 68.5% leading to:

0.685* 3,689,694= 2,527,440 cases of UAPP

**Study**	**Study Year**	**Sample**	**Morbidity (%)**
Tomenson & Matthews	2006	251	63.4
Uribe et al 2012	2009-2010	132	56
Varona et al 2016	2014-2015	339	86.1
Average			*68.5*

WHO Mortality data: available for 2017-2021, 15.6 fatalities per year, 2.4 child fatalities per year, farmer fatalities were 1 per year.

World Bank data on populations of Colombia from 2017-2021 were used.

Annual national estimates for Colombia

Population type	Year	Population	Fatal	Non-fatal	Total cases
General	2017-2021	50,052,652	15.6		15.6
Children	2017-2021	11,207,169	2.4		2.4
Farming/occupational	2006, 2009-2010, 2014-2015	3,689,694		2,527,440	
	2017-2021	4,148,162	1		2,527,441

National estimates based on studies with following study characteristics

Criteria	Farming/occupational population
Number of studies used for calculation	3
Review periods covered (first, second, both)	first
Sampling (representative, simple sample or unspecified)	representative
Diagnose (study scientists, self-reported)	self-reported
Latency period (24 h, 48 h, other, unspecified)	other
At-risk time (annual, <1 y, other, ever, unspecified)	annual
No. of symptoms (>=1, >=2, unspecified)	unspecified

14. Costa Rica

Publications extracted for synthesis 2006-2018: 1

Tomenson and Matthews 2009 (4)

- Period of study = 2005-2006
- Survey of pesticide applicators and farmers, commissioned by Syngenta Crop Protection among 2,431 users in eight countries
- Sample = 250, representative
- Self-reported symptoms
- Morbidity = 32% (80 cases, reported minor to serious incidents)
- Data ok to extrapolate

Publications extracted for synthesis 2019-2023: 1

Farnham et al. 2021 (53)

- Period of study = May-August 2016
- Sample =300, survey of conventional and organic farmers from a county in Costa Rica, male
- Self-reported symptoms
- Morbidity = 14% of farmers were reported experiencing 'ever' poisoning, once or more in a lifetime
- Data not included because of 'ever' at-risk prevalence

WHO Mortality data: available from 2017-2020: 1.75 deaths per year; 0.5 child death per year; 0.25 farmer deaths per year.

Estimation of national figures

Population data were taken from World Bank for the study year 2006.

Annual national estimate for Costa Rica

Population type	Year	Population	Fatal	Non-fatal	Total cases
General	2017-2020	5,060,553	1.75		1.75
Children	2017-2020	1,082,449	0.5		0.5
Farming/occupational	2006	259,564	NA	83,060	
	2017-2020	386,874	0.25		83,060.25

National estimates based on studies with following study characteristics

Criteria	Farming/occupational population
Number of studies used for calculation	1
Review periods covered (first, second, both)	first
Sampling (representative, simple sample or unspecified)	representative
Diagnose (study scientists, self-reported)	self-reported
Latency period (24 h, 48 h, other, unspecified)	unspecified
At-risk time (annual, <1 y, other, ever, unspecified)	annual
No. of symptoms (>=1, >=2, unspecified)	unspecified

15. Cote d’Ivoire

Publications extracted for synthesis 2006-2018: 1

Publications extracted for synthesis 2019-2023: 0

Ajayi et al. 2008 (54)

- Period of study = unclear but likely around or after 2006; paper also references a population recruited in a paper from 2007, so it’s possible the data were collected pre-2006
- Sample = 132, survey poisonings among cotton farmers from Cote d’Ivoire
- Self-reported symptoms, “once for every five times (20%) that they carried out spraying operations. These were the symptoms that began during or within 24 hours after spraying operation”
- Morbidity =20% (26 cases)

WHO Mortality data: no mortality data were available.

Estimation of national figures

World Bank data on agricultural employment reported 3,185,756 farmers, while the number of cotton farmers in 2006 in Cote d’Ivoire was reported as 72,000 in a 2010 World Bank Report.

0.20 * 72,000 = 14,400 for cotton farmers gives the lower boundary estimate.

Annual national estimates for Cote D’Ivoire

Population type	Year	Population	Fatal	Non-fatal	Total cases
Farming/occupational, lower boundary	2006	72,000		14,400	14,400

National estimates based on studies with following study characteristics

Criteria	Farming/occupational population
Number of studies used for calculation	1
Review periods covered (first, second, both)	first
Sampling (representative, simple sample or unspecified)	representative
Diagnose (study scientists, self-reported)	self-reported
Latency period (24 h, 48 h, other, unspecified)	24 h
At-risk time (annual, <1 y, other, ever, unspecified)	unspecified
No. of symptoms (>=1, >=2, unspecified)	unspecified

Source of population data

Gergely N. The Cotton Growing Sector of Cote d'Ivoire. World Bank; Africa Region Working Paper Series No. 130 (a); June 2010. Available from: http://documents.worldbank.org/curated/en/552341468247813306/The-cotton-sector-of-Cote-dIvoire

From the report (p.17): "The number of cotton growers was estimated at 72,000 for the 2005/06 season (based on seed cotton delivery reports from the ACE), which, for an area devoted to cotton production of 300,000 ha for that season, gives an average cotton production area of 4.2 ha per farm."

16. Ecuador

Publications extracted for synthesis 2006-2018: 1

Publications extracted for synthesis 2019-2023: 0

Gonzalez-Andrade et al. 2010 (55)

- Period of study: only 2006-2007 data were used for data extraction
- Sample = the general population of Ecuador from 2006 to 2007, obtained from FAOSTAT
- Morbidity = 0.0154%
- Reported ICD-10 codes from Ecuador's National Register of Hospital Admissions/Discharges and data from the Ministry of Public Health from 2001-2010

WHO Mortality data: available for the years 2017-2021 showing 38 annual fatalities for the general population, 1.6 case for children, and 0.6 case for the farming population.

Estimation of national figures

Based on the data reported from 2006-2007 in Gonzalez-Andrade et al. 2010, for the general population the estimated non-fatal cases were 2,168 per year.

Table: National estimate for Ecuador

Population type	Year	Population	Fatal	Non-fatal	Total cases
General	2006-2007	14,086,467		2,168	
	2017-2021	17,288,538	38		2,206
Children	2017-2021	4,665,194	1.6		1.6
Farming/occupational	2017-2021	2,477,360	0.6		0.6

Limitations

The authors reported that the exposure "seems to be" mostly unintentional—the register did "not contain information about the location of the exposures but indicates that the poisonings in this study occurred in the workplace". Thus, we assumed that the data reported in the paper have a negligible number of intentional poisonings. An additional assumption was made that the number of fatalities was distributed evenly across the years that data were collected.

17. Egypt

Publications extracted for synthesis 2006-2018: 1

Publications extracted for synthesis 2019-2023: 0

Lein et al. 2012 (56)

- Period of study = 2009
- Area of study = Menoufia
- Sample = 31 Egyptian pesticide applicators, applying pesticides to cotton; exposed to chlorpyrifos and limited pyrethroids, males
- Self-reported symptoms
- Morbidity = 61.3% (19 cases)
- There are 1,080 cotton field spray applicators in Menoufia, one of 29 governates of Egypt; sample representative of cotton workers but query whether it can be extrapolated to other farmers and workers; paper states “The Egyptian pesticide workers provide a population with a relatively consistent exposure history stretching back for many years. No information on cotton growing in other governates, although it appears Menoufia is the main area
- Data were not extrapolated because of a combination of small sample size and inadequate data on population

WHO Mortality data: available from 2017-2019: annualized 60.3 fatal poisonings per year reported for the general population of Egypt, including 11.7 for children.

Estimation of national figures

As we were not able to find information on the population of cotton growers or cotton pesticide applicators in Egypt, a non-fatal occupational rate cannot be estimated.

Annual national estimates for Egypt

Population type	Year	Population	Fatal	Non-fatal	Total cases
General	2017-2019	103,716,274	60.3		60.3
Children	2017-2019	34,667,101	11.7		11.7

18. Ethiopia

Publications extracted for synthesis 2006-2018: 3

Nigatu et al. 2016 (57)

- Period of study = 2014
- Area of study = 1025 villages in Oromia region
- Flower farm workers
- Sample = 516, representative sample, 58% male
- Self-reported symptoms, with those reporting two or more typical pesticide intoxication symptoms being assessed as having UAPP
- Morbidity = 26.36% (136 cases)
- Data ok to extrapolate

Adinew et al. 2017 (58)

- Period of study = 2010-2014
- Area of study = north Gondar area
- Sample = 543, registered poisoning cases admitted to emergency centres for three hospitals serving an approximate population of 5,000,000 in the north Gondar area. Most of the cases involved patients >15 years. Some cases were intentional. No data on children
- Morbidity = 0.00026 % (13 cases)
- Data ok to extrapolate

Negatu et al. 2018 (59)

- Period of study = not given but presumed to be post-2006
- Area of study = central eastern Ethiopia
- Pesticide applicators from small-scale irrigated farms, a large-scale open farm and a large-scale greenhouse (commercial agriculture)
- Sample = 256; representative sample of workers, males
- Morbidity = 16.01% (41 cases)
- ‘Ever’ timeframe
- Data not used in second synthesis because of ‘ever’ prevalence

Publications extracted for synthesis 2019-2023: 3

Loha et al. 2022 (60)

- Period of study = 2015
- Area of study = Central Rift Valley
- Sample = 333, survey of farmers
- Morbidity = 69% (230 cases) reporting feeling discomfort or illness
- ‘Ever’ timeframe
- Data not used because of ‘ever’ timeframe

Tessema et al. 2021 (61)

- Period of study = 2019

- Area of study = six districts were selected based on the extent of pesticide use and agricultural activities out of 27 districts in the east Hararge Zone and Harari region
- Sample = 254, surveys of extension officers, 78% male
- 97 cases; Ethiopian farmers experiencing pesticide poisoning responding yes, according to the officers
- 'Ever' timeframe
- Data not used because of 'ever' at-risk timeframe

Tessema et al. 2022 (62)

- Period of study = 2017
- Area of study = East Harage Zone within the region of Oromiya; of 18 districts of the zone, the study was conducted in three districts
- Sample = 803, survey of residents (83% male) and pesticide applicators (93% female)
- Morbidity = 35.99% (289 cases)
- 'Ever' timeframe
- Data not used because of 'ever' at-risk timeframe

WHO Mortality data: no mortality data were available.

Estimation of national figures

Negatu et al 2018, Loha et al. 2022, Tessema et al. 2021 and 2022 reported 'ever' at-risk times. The studies were therefore not included in the estimates.

For the general population, we used the results from Adinew et al. (2017). The general population was averaged from 2010-2014 and used to calculate poisoning figures for the entire general population:

0.0000026 * 92,489,621= 240.

For the farming/occupational population, we used the study of Nigatu et al. (2016) who reported an UAPP prevalence of 26% among flower plantation workers. The size of the farming population was taken from World Bank for 2015.

Annual national estimates for Ethiopia

Population type	Year	Population	Fatal	Non-fatal	Total cases
General	2014-2016	92,489,621		240	240
Farming/occupational	2015	32,923,713		8,560,165	8,560,165

National estimates based on studies with following study characteristics

**Criteria**	**Farming/occupational population**
Number of studies used for calculation	1
Review periods covered (first, second, both)	first
Sampling (representative, simple sample or unspecified)	representative
Diagnose (study scientists, self-reported)	study scientists
Latency period (24 h, 48 h, other, unspecified)	48 h
At-risk time (annual, <1 y, other, ever, unspecified)	annual
No. of symptoms (>=1, >=2, unspecified)	>=2

19. France

Publications extracted for synthesis 2006-2018: 1

Publications extracted for synthesis 2019-2023: 0

Baldi et al. 2014 (63)

- Period of study = 2008
- Data from the AGRICAN cohort, which surveyed a representative sample of farmers and workers, 54.8% male
- Morbidity = 6.1 % (845 cases), "A history of pesticide poisoning was reported by 845 individuals (6.1%) among the 13,900 who completed the information"
- Data ok to extrapolate

WHO Mortality data: data from 2017-2020 were available, with 1 death occurring per year in the general population and zero cases for children and the farming population.

Estimation of national figures

The only study available for non-fatal UAPP is Baldi et al. The question of the study was "Have you ever been intoxicated by a pesticide?" with answer categories: "Never - Once - Several times. If yes, in which year(s)." Three answers were possible. The study reports the prevalence of poisoning with no indication that only the "once" (i.e., 'ever') category was analysed. The enrolment phase was 2005-2007. We based the annual national estimates on this study, according to our protocol when no other studies were available: 0.061* 751,471=45,839.

Annual national estimates for France

Population type	Year	Population	Fatal	Non-fatal	Total cases
General	2017-2020	67,258,869	1		1
Farming	2008	751,471	0		0
	2017-2020	761,786		45,839	45,839
Children	2017-2020	11,945,721	0		0

National estimates based on studies with following study characteristics

Criteria	Farming/occupational population
Number of studies used for calculation	1
Review periods covered (first, second, both)	first
Sampling (representative, simple sample or unspecified)	representative
Diagnose (study scientists, self-reported)	self-reported
Latency period (24 h, 48 h, other, unspecified)	unspecified
At-risk time (annual, <1 y, other, ever, unspecified)	ever
No. of symptoms (>=1, >=2, unspecified)	unspecified

20. Gambia

Publications extracted for synthesis 2006-2018: 1

Publications extracted for synthesis 2019-2023: 0

Idowu et al. 2017 (64)

- Period of study = after 2013
- Area of study = Kombo North District of the Gambia
- Sample = 66, representative sample of farmers, 76% females
- Self-reported symptoms of poisoning
- Morbidity = 51.52% (34 cases)
- Data ok to extrapolate

WHO Mortality data: no mortality data were available.

Estimation of national figures

As the data were collected after 2013, an average was taken of the most recent data after 2013 on the farmer population (2014-2016 World Bank data).

0.5152 * 162,169 = 83,549.5

Annual national estimate for Gambia

Population type	Year	Population	Fatal	Non-fatal	Total cases
Farming/occupational	2014-2016	162,169		83,549	83,549

National estimates based on studies with following study characteristics

Criteria	Farming/occupational population
Number of studies used for calculation	1
Review periods covered (first, second, both)	first
Sampling (representative, simple sample or unspecified)	representative
Diagnose (study scientists, self-reported)	self-reported
Latency period (24 h, 48 h, other, unspecified)	unspecified
At-risk time (annual, <1 y, other, ever, unspecified)	unspecified
No. of symptoms (>=1, >=2, unspecified)	unspecified

21. Georgia

Publications extracted for synthesis 2006-2018: 1

Publications extracted for synthesis 2019-2023: 0

PAN UK 2015 (65)

- Period of study = 2016
- Area of study = Kvemo Kartli, Georgia
- In this area main crops include grains (corn, barley, wheat) and vegetables (potatoes, tomatoes, onions, beet cucumber). Fruit orchards are common on small holdings and a small number of large commercial farms grow orchard fruits
- Sample = 920, representative of farmers and workers, male and female workers
- Self-reported data on symptoms of poisonings over the previous 12 months
- Morbidity = 20% (184 cases)
- Data ok to extract

WHO Mortality data: reported for 2017-2022, with 2.83 deaths per year for the general population, 0 for the farming population, and 0 for children.

Estimation of national figures

Annual national estimates for Georgia

Population type	Year	Population	Fatal	Non-fatal	Total cases
General	2017-2022	3,719,757	2.83		2.83
Children	2017-2022	772,168	0		0
Farming/occupational	2016	740,545		148,109	
	2017-2022	760,088	0		148,109

National estimates based on studies with following study characteristics

Criteria	Farming/occupational population
Number of studies used for calculation	1
Review periods covered (first, second, both)	first
Sampling (representative, simple sample or unspecified)	representative
Diagnose (study scientists, self-reported)	self-reported
Latency period (24 h, 48 h, other, unspecified)	unspecified
At-risk time (annual, <1 y, other, ever, unspecified)	annual
No. of symptoms (>=1, >=2, unspecified)	unspecified

22. Germany

Publications extracted for synthesis 2006-2018: 1

Publications extracted for synthesis 2019-2023: 0

Moebus & Bödeker 2015 (66)

- Period of study = 2005-2010
- National mortality data
- No fatalities

WHO Mortality data: reported for 2017-2020 showed 0.75 fatal cases per year within the general population. No cases were reported for children and farmers.

Annual National estimates for Germany

Only WHO mortality data were reported in the table below.

Population type	Year	Population	Fatal	Non-fatal	Total cases
General	2017-2020	82,954,154	0.75		0.75
Farming/occupational	2017-2020	543,617	0		0
Children	2017-2020	11,262,038	0		0

23. Ghana

Publications extracted for synthesis 2006-2018: 1

Publications extracted for synthesis 2019-2023: 0

Ae-Ngibise et al. 2015 (67)

- Period of study = December 2011
- Area of study = Kintampo North and South municipalities
- Cross-sectional survey of randomly selected farmers (n=1,040)
- Self-reported data on frequency of use and symptoms after pesticide use
- Morbidity = 39.04 % (406 cases), for the most prevalent symptom (skin irritation)
- Data ok to extrapolate for farmer population

WHO Mortality data: no mortality data were available.

Estimation of national figures

The farming/occupational population for 2011 was taken from World Bank

39.04 % *4,592,281 = 1,792,758

Annual national estimates for Ghana

Population type	Year	Population	Fatal	Non-fatal	Total cases
Farming/occupational	2011	4,592,281		1,792,758	1,792,758

National estimates based on studies with following study characteristics

Criteria	Farming/occupational population
Number of studies used for calculation	1
Review periods covered (first, second, both)	first
Sampling (representative, simple sample or unspecified)	representative
Diagnose (study scientists, self-reported)	self-reported
Latency period (24 h, 48 h, other, unspecified)	unspecified
At-risk time (annual, <1 y, other, ever, unspecified)	unspecified
No. of symptoms (>=1, >=2, unspecified)	unspecified

24. Hungary

Publications extracted for synthesis 2006-2018: 0

Publications extracted for synthesis 2019-2023: 1

Tessema et al. 2021 (61)

- Period of study = September-December 2020
- Area of study = 13 Hungarian counties and the capital Budapest
- Sample = 92; extension workers and plant doctors, whose main task is to ensure safe pesticide use, their duties range from advice to the individual farmers to adopt self-protective behaviour to the farming companies to proper handling, effective and safe use of agrochemicals including pesticides in their control area". 78% male, pesticide exposure is likely limited
- Due to the onset of the COVID-19 pandemic, a web-based survey was conducted instead of a field-survey, which included the question whether (yes or no) pesticide poisoning was experienced "in the past"
- Morbidity = 6.5% (6 cases)
- 'Ever' timeframe

WHO Mortality data: On average, 0.67 fatal pesticide poisonings per year were reported in the WHO Cause of Death database for the general population of Hungary (available for 2017-2022). Zero UAPP reported for children and farming population.

National estimates for Hungary

Population type	Year	Population	Fatal	Non-fatal	Total cases
General	2017-2022	9,739,627	0.67		0
Farming/occupational	2020	224,737		14,608	14,608
	2017-2022		0		
Children	2017-2022	1,415,110	0		0

National estimates based on studies with following study characteristics

Criteria	Farming/occupational population
Number of studies used for calculation	1
Review periods covered (first, second, both)	second
Sampling (representative, simple sample or unspecified)	representative
Diagnose (study scientists, self-reported)	self-reported
Latency period (24 h, 48 h, other, unspecified)	unspecified
At-risk time (annual, <1 y, other, ever, unspecified)	ever
No. of symptoms (>=1, >=2, unspecified)	unspecified

25. India

Publications extracted for synthesis 2006-2018: 11

Tomenson and Matthews 2009 (4)

- Period of study = 2006
- Sample = 259, representative, farmers and workers using pesticides
- Morbidity = 39% (101 cases), any symptom of UAPP over past 12 months
- Data ok to extrapolate

Shetty et al. 2011 (68)

- Period of study = date not given, may be pre-2006
- Area of study = 290 randomly selected villages 28 districts in 12 states covering different agroecological zones in India growing crops such as paddy, cotton, sugarcane, wheat, apple, pomegranate, mango, grapes and vegetables
- Sample =1577; representative, farmers, workers, agricultural officers, pesticide vendors and doctors,
- Morbidity = 19.4% (306 cases); experienced symptoms after handling pesticides – but this also includes vendors and agricultural officers as well as farmers and workers
- Data not ok to extrapolate because at-risk population is not well defined

Patil and Katti 2012 (69)

- Period of study = 2010
- Area of study = a cash crop zone in Maharashtra, from Shirol region of Kolhapur district
- Sample = 78 workers; representative, closely linked with applying pesticides, 73% males
- Morbidity = 97.44% (76 cases), for most prevalent symptom
- Data ok to extrapolate

Banerjee et al. 2014 (70)

- Period of study = 18 months from January 2008
- Area of study = Burdwan, West Bengal
- Sample = 500 (male), cross-sectional study of farmers purchasing pesticides from the Bardhaman Thana Co-operative Agricultural Marketing Society Ltd
- Morbidity = 29.8% (149 cases), for most prevalent symptom
- Data ok to extrapolate

Choudhary et al. 2014 (71)

- Period of study = 2009-2011 (18 months)
- Area of study = 3 villages (Bagarauda, Jhagariya Khurd, Babadiya Khurd) in Misrod, southern suburb of Bhopal, Madhya Pradesh
- Sample = 105; representative, but all male farmers, principally soybean crops
- Organophosphates only
- Symptoms reported by questionnaire, during or immediately after spraying
- 50% of farmers exposed for 18 months; 20% of farmers exposed for 12 months, and 8.2% exposed for 6 months

- Morbidity averaged over the 3 time periods = 26.7% (28 cases), for most prevalent symptom
- The study indicates that the longer farmers are exposed to pesticides, the more symptoms of UAPP they display during or immediately after spraying
- “Subjects who self-reported have had two or more of the mentioned symptoms within 24 hours after pesticide spraying were considered to have suffered acute pesticide poisoning.” However, the paper does not report how many there were
- Data limited to OPs so not ok to extrapolate to all other pesticides

Peshin et al. 2014 (72)

- Period of study = 1999-2012
- Based on telephone calls to National Poisons Information Centre, All India Institute of Medical Sciences, New Delhi
- Annual average morbidity = 130/year, no population given
- Bears no relationship to results from surveys
- Data not ok to extrapolate for the general population; phone calls to one centre without information on the catchment area and the coverage

Kumar 2015 (73)

- Period of study = 2014-15
- Area of study = 15 workers from the States of Arunachal Pradesh (small scale tea gardens) and Andhra Pradesh (daily labourers working in small scale tea gardens in Namsai district but also daily labourers working in cotton, paddy, and vegetable farms; and 8 male workers from Andhra Pradesh, Madhya Pradesh, Telangana, West Bengal
- Sample = 23; purposive, male and female workers, paraquat users
- Paraquat only
- Morbidity = 91.30% (21 cases), for any symptom after spraying
- Data not ok to extrapolate beyond paraquat sprayers

Kaur 2016 (74)

- Period of study = presumed 2010
- Area of study = Malwa region of Punjab
- Sample = 88; farm workers, male, purposive sampling, 99% of whom were engaged in spraying pesticides daily during harvesting season (crop not identified)
- Morbidity = 90.91% (80 cases), for highest symptom prevalence
- Study is not representative of all farmers and all farm workers but rather reflects those workers who spray frequently
- Does not identify a time period over which the symptoms occurred
- Date not ok to extrapolate

Kumari and John 2018 (75)

- Period of study = 2016 (assumed)

- Area of study = 8 randomly selected villages of Kinnaur district of Himachal Pradesh (Sangla, Rakcham, Kamroo, Sungra, Nichar, Kangos, Lutuksa and Bhabanagar) of Himachal Pradesh
- Sample = 96 workers; representative, 81 males, in apple orchards
- Morbidity = 84% (81 cases); for most prevalent symptom, "during and after the spray season"
- Data ok to extrapolate

Rengam et al. 2018 (9)

- Period of study = 2017
- Area of study = Chittoor, Andhra Pradesh
- Sample = 80, farmers and workers in Mango orchards
- Morbidity = 100% (80 cases), for any symptom
- Data ok to extrapolate

National Crime Records Bureau 2016 (76)

- A report by the National Crimes Records Bureau was available for 2014 and 2015 on accidental deaths from pesticides (NCRB 2016)

Year	Total deaths
2014	5,915
2015	7,060
average	*6,488*

Publications extracted for synthesis 2018-2023: 8

PAN India et al 2020 (77)

- Period of study = 2019
- Area of study = Tamil Nadhu
- Sample = 24; survey in community, child workers in floriculture, aged 9-13
- Morbidity = 87.5% (21 cases)
- Data ok to extrapolate

Reshma and Jayalakshmi 2020 (78)

- Period of study = 2018; 12 months
- Area of study = Idukki district, Kerala
- Sample = 300, workers in cardamom plantations, 79.3% males.
- Descriptive cross-sectional study
- Morbidity = 100% (300 cases), within 24hr of spraying
- Data ok to extrapolate

Thakur et al 2020 (79)

- Period of study = 2019
- Area of study = 3 villages in Gujarat, Maharastra

- Community-based cross-sectional study; representative sample
- Sample = 380, all agricultural workers who were > 18 years of age and were handling pesticides for > 1 year were included in study. 53% males.
- Morbidity = 52.11% (198 cases), highest prevalence symptom
- Data ok to extrapolate

Kumari et al. 2021 (80)

- Period of study = March-August 2017
- Kinnaur district of North India
- Sample = 96, Population-based cross-sectional survey among apple orchard workers (81 male, 15 female)
- Self-reported acute health issues among the pesticide applicators
- Morbidity = 85% (82 cases), for at least one symptom
- Data ok to extrapolate

Diyana et al. 2022 (11)

- Period of study = 2021
- Area of study = Yavatmal district (n = 66: 16 women, 50 men); Wyanad District (n = 35: 17 women and 18 men), in Wyanad only 18 respondents used pesticides
- Sample = 84, representative sample of farmers and workers
- Morbidity = 39.2% (33 cases)
- Data ok to extrapolate

Lohan et al. 2022 (81)

- Period of study = 2012-2015
- Punjab
- Sample = 43,177 sprayers, both males and females in the study but sex of nonfatal UAPP not reported
- Mortality and morbidity are those who received compensation for injury, so not representative of all UAPP
- Mortality = 0.2264% (98 fatalities)
- Morbidity = 0.0185% (8 cases)
- Date not ok to extrapolate as there is a lack of information on the completeness of the register

Lari et al. 2023 (82)

- Period of study = not specified, assumed to be after 2006 because of the publication year
- Area of study = Rangareddy district, Telangana
- Sample = 180, community-based survey of farm workers, 113 male, 67 female
- Self-reported symptoms
- Morbidity = 95% (173 cases), for most prevalent symptom
- Data ok to extrapolate

National Crime Records Bureau (NCRB 2022) (76)

- Period of study = 2020-2021

- A report by the National Crimes Records Bureau was available for the whole country on deaths from accidental intake of pesticides

Year	Total deaths	Non-fatal cases
2020	7,402	
2021	7,950	44
Average	7,676	

For the year 2021, 178 fatal cases are reported for children <14 years.

WHO Mortality data: no mortality data were available.

Estimation of national figures

We averaged the percentages of acute pesticide poisonings across studies referring to farmers/workers and excluded those that report on specific pesticides only (paraquat, OPs) or populations (sprayer), because there are sufficient more general studies. We based the extrapolation on an average farmers/workers population for the respective years. Although some studies were of specific crops, e.g. apple, cardamon, flowers, cotton, orchards, all of these crops are widespread in India and there are not particular conditions affecting any of these that would render the results too specific to be extrapolated to farming in general. For the general population, we averaged the figures from 2020 and 2021 of the National Crime Bureau.

Results from Peshin et al. 2014 were not used. No information on the catchment area and the coverage was given so this might not be related to the total population.

Results from Shetty et al. 2011 were not used: agricultural extension officers, vendors and doctors were combined with farmers and workers.

For children, one study gave occupational morbidity and National Crime Bureau gave mortality. Child morbidity is included in occupational figures, as it would not be reflective of morbidity among children in the general population.

We based our extrapolation on the average morbidity of all included studies.

Study	Study Year	Sample	Morbidity (%)
Tomenson and Mathews 2009	2006	259	39
Banerjee 2014	2008-10	500	29.8
Patil and Katti 2012	2010	78	97.4
Choudhary et al. 2014	2009-11	28	26.7
Kumari & John 2018	2016	91	84
Rengam 2018	2017	80	100
Kumari et al 2021	2017	96	85
Reshma & Jayalakshmi 2020	2018	300	100

Thakur et al 2020	2019	380	52.1
PAN India et al 2020	2019	24	87.5%
Diyana et al 2022	2021	84	39.3
Lari et al 2023	?	180	95.8
Average			*73.6*

Annual National estimates for India: 2006-2023

**Population type**	**Year**	**Average Population size**	**Fatal**	**Non-fatal**	**Total cases**
General	2020-2021	1,401,975,485	7,676		7,676
Child <14 yrs	2021	361,569,242	178		178
Farming/occupational	2006-2010,2016-2023	229,940,444		169,305,149	169,305,149

National estimates based on studies with following study characteristics

**Criteria**	**Farming/occupational population**
Number of studies used for calculation	11
Review periods covered (first, second, both)	both
Sampling (representative, simple sample or unspecified)	representative
Diagnose (study scientists, self-reported)	self-reported
Latency period (24 h, 48 h, other, unspecified)	24 h
At-risk time (annual, <1 y, other, ever, unspecified)	annual
No. of symptoms (>=1, >=2, unspecified)	>=1

26. Indonesia

Publications extracted for synthesis 2006-2018:4

Sekiyama et al. 2007 (83)

- Period of study = 2006
- Area of study = two villages in the watershed of the Citarum River, Bandung district, West Java (crops, tea planting, dairy farming)
- Sample = 73 farmers
- Morbidity = 63% (46 cases), for most prevalent symptom; farmers experienced an average of 6.7 symptoms each; ever experienced after spraying
- Data were included in first synthesis, not included in second extrapolation because of lifetime prevalence

Tomenson and Matthews 2009 (4)

- Period of study = 2006
- Sample = 290, representative sample of farmers and workers using pesticides
- Morbidity = 28.28% (82 cases) any symptom of APP over past 12 months
- Data ok to extrapolate

Perwitasari et al. 2017 (84)

- Period of study = 2006
- Area of study = Kulon Progo County
- Sample = 84, farmers using OPs during onion planting season, recruited from 3 onion farmer groups, 85.7% male
- Organophosphates only
- Morbidity = 39.29% (33 cases), for any symptom
- Not used for extrapolations because only organophosphates were studied

Rengam et al. 2018 (9)

- Period of study = 2016-17
- Area of study = North Sumatra
- Sample = 57, oil palm plantation workers selected from 4 plantations (different companies), 93 males, 27 females
- Morbidity = 96 % (55 cases), for most prevalent symptom
- There are 10,400,000 plantation workers in Indonesia (Zidane 2018)
- Data ok to extrapolate

Publications extracted for synthesis 2019-2023: 2

Setyopranoto et al 2020 (85)

- Period of study = not specified
- Area of study = a Central Java village
- Sample = 120, cross-sectional study of farmers who used pesticides, 77.5% male
- Morbidity = 68.3% (82 cases), as determined by cholinesterase levels

- Data ok to extrapolate

Sodikin et al. 2020 (86)

- Period of study = March-June 2019
- Area of study = Barito Utara district, Teweh Selatan subdistrict
- Sample = 55, cross-sectional study of farmers
- Morbidity = 56.36 % (31 cases), based on cholinesterase inhibition

WHO Mortality data: no mortality data were available.

Estimation of national figures

**Study**	**Study year**	**Sample**	**Morbidity (%)**
Rengam et al. 2018	2016-17	57	96
Tomenson & Matthews 2009	2006	290	28.3
Setyopranoto et al 2020	Not stated	120	68.3
Sodikin et al. 2020	2019	55	56.4
Average			*62.3*

Annual national estimates for Indonesia

**Population type**	**Year**	**Population size**	**Fatal**	**Non-fatal**	**Total cases**
Total farmers/workers	2006, 2016-17, 2019	40,517,515		25,242,412	25,242,412

National estimates based on studies with following study characteristics

**Criteria**	**Farming/occupational population**
Number of studies used for calculation	4
Review periods covered (first, second, both)	both
Sampling (representative, simple sample or unspecified)	representative sample
Diagnose (study scientists, self-reported)	self-reported
Latency period (24 h, 48 h, other, unspecified)	unspecified
At-risk time (annual, <1 y, other, ever, unspecified)	annual
No. of symptoms (>=1, >=2, unspecified)	>=1

Source:

Zidane. Indonesia: Exploitation of Women and Violation of Their Rights in Oil Palm Plantations. Bulletin 236; World Rainforest Movement; 2018 March 7. Available from https://www.wrm.org.uy/bulletin-articles/indonesia-exploitation-of-women-and-violation-of-their-rights-in-oil-palm-plantations

27. Iran

Publications extracted for synthesis 2006-2018: 5

Ahmadi et al. 2010 (87)

- Period of study = 2006-2008
- Area of study = Sari city, Mazandaran province, Northern Iran
- Descriptive and retrospective study conducted at poisoning ward of Imam teaching hospital
- Morbidity = 0.0018% (9 cases), general population, estimated population of over 500,000
- Hospitalised cases only
- Data ok to extrapolate

Hashemi et al. 2012 (88)

- Period of study = date not stated
- Area of study = Ashjerd village of the County Marvdasht in Fars Province in the southwestern part of Iran
- Sample = 132, representative sample of farmers "who usually engage in" pesticide use and handling, all male
- Morbidity = 37% (64 cases), for any symptom, ever experienced after spraying
- Excluded from extrapolation because of the 'ever' prevalence

Soltaninejad et al. 2012 (89)

- Period of study = 2007-2010
- Patients with acute "rice tablet" poisoning who were admitted to Loghman Hakim Hospital Poison Center, Tehran
- Aluminium phosphide only
- Catchment area of hospital has a population of 14,276,978
- Mortality = 0.000007% (1 case)
- Morbidity = 0.000021% (3 cases)
- Data not ok to extrapolate as single pesticide only that is not representative of other pesticide use

Afshari et al. 2018 (90)

- Period of study = 2017
- Area of study = Twiserkan County, Hamadan province, west Iran, an important province in agricultural production (wheat, barley, garlic, almonds, peach, plum, apple, and especially walnuts)
- Sample = 474; farmers and workers who had used pesticides at least 1 week before interview, 73% of farmers themselves sprayed pesticides, 98% male
- Morbidity = 60.34% (286 cases), for at least one symptom within 48 hrs of spraying, during the past 7 days
- Data ok to extrapolate

Sharafi et al. 2018 (91)

- Period of study = date not stated

- Area of study = Kermanshah province
- Sample = 311, representative farmers, all male, older than 21 years old, their main income was depended on agriculture, and were working on minimum 5 and maximum 10 ha of agricultural lands during the past year
- Growing wheat and barley (68.1%), chickpea (21.3%), and industrial crops (8.0%). Industrial crops include corn, sugar beet, and rapeseed
- Morbidity = 80.7% (251 cases), for most prevalent symptom
- Data ok to extrapolate

Publications extracted for synthesis 2019-2023: 1

Sharifzadeh et al. 2019 (92)

- Period of study = June 2016-February 2018
- Area of study = Sari County of Mazandaran Province in the southern Caspian Sea sub-region of northern Iran
- Sample = 373, representative, rice farmers, all male
- Symptoms self-reported
- Morbidity = 53.9% (201 cases)
- Data ok to extrapolate

WHO Mortality data were available for 2017 showing 39 fatalities in total including 3 for children and 2 for the farming/occupational population.

Estimation of national figures

The study by Hashemi et al 2012 was not included in the extrapolation because the time frame is unclear and the symptoms were 'ever' experienced poisonings. The publication of Soltaninejad et al. 2012 was not included in the extrapolation because only one pesticide was studied and it is not representative of other pesticides' use.

For the farming/occupational population we averaged the three remaining studies on farmers and workers to arrive at an occupational morbidity of 65%. We based the extrapolation on an average farming population of 4,645,991.

The study by Ahmadi et al 2010 does not give information on the catchment area and coverage of the hospital, so the study results were not considered for national estimates.

Synthesis of studies

Study	Study year	Sample	Morbidity (%)
Afshari 2018	2017	474	60.3
Sharafi 2018	?	311	80.7
Sharifzadeh et al. 2019	2016-2018	373	53.9
Average			*65*

Annual national estimate for Iran

Population type	Year	Population size	Fatal	Non-fatal	Total cases
General	2017	84,505,076	39		39
Children	2017	20,259,996	3		3
Farming/occupational	2016-18	4,645,991	2	3,019,894	3,019,896

National estimates based on studies with following study characteristics

Criteria	Farming/occupational population
Number of studies used for calculation	3
Review periods covered (first, second, both)	both
Sampling (representative, simple sample or unspecified)	representative sample
Diagnose (study scientists, self-reported)	self-reported
Latency period (24 h, 48 h, other, unspecified)	24 h
At-risk time (annual, <1 y, other, ever, unspecified)	annual
No. of symptoms (>=1, >=2, unspecified)	unspecified

28. Italy

Publications extracted for synthesis 2006-2018: 1

Publications extracted for synthesis 2019-2023: 0

Settimi et al. 2007 (93)

- Period of study = 2006, February 1-March 31
- Area of study = Milan, Pavia, and Bergamo, in the Piedmont region
- Accidental poisoning cases from poison control centres
- Piedmont area population = 4,340,000
- Morbidity = 0.00082949% (6 cases in 2 months = 36 cases per year; n=482)

WHO Mortality database: reported data as follows from 2017- 2020, with 2.5 fatalities per year for the general population, 0 for the farming population, and 0 for children.

Estimation of national figures

The Piedmont region population was reported in a 2009 OECD book (OECD 2009) as 4.34 million in 2006, and the prevalence of poisoning extrapolated to the year 2006 from the Piedmont region was 0.00082949%. The general population for Italy in 2006 was 58,143,979.

0.00082949% * 58,143,979 = 482

Annual national estimates for Italy

Population type	Year	Population	Fatal	Non-fatal	Total cases
General	2006	58,143,979		482	
	2017-2020	60,031,600	2.5		484.5
Children	2017-2020	7,895,742	0		0
Farming/occupational	2017-2020	994,671	0		0

Sources

OECD Reviews of Regional Innovation: Piedmont, Italy 2009, p. 72 Table 2.1 Population and change 1990-2006, by Italian region; OECD publishing; 2009. Available from: https://books.google.com/books?id=hFdvgXvIv6wC&dq=population+of+piedmont+italy+2006&source=gbs_navlinks_s

29. Jamaica

Publications extracted for synthesis 2006-2018: 1

Publications extracted for synthesis 2019-2023: 0

Ncube et al. 2011 (94)

- Period of study = 2006
- Area of study = 3 parishes of northwestern Jamaica
- Sample = 349, population survey of farmers, 87% males. Study reported that 21% of the poisonings were the female farmers
- Self-reported symptoms: “one or more incidents of acute pesticide poisoning within the last two years”
- Morbidity = 16 % (57 cases)
- Data ok to extrapolate

WHO Mortality database: no data available for the period after 2017, we used annualized data for general population (0.7. cases/year) and 0 for children and farming population.

Estimation of national figures

As there is only one study available, we base the national estimation on Ncube et al. even though an at-risk time of 2 years was reported. The farming/occupational population was taken from World Bank for 2006 as 212,508.

National estimates for Jamaica

Population type	Year	Population	Fatal	Non-fatal	Total cases
General	2009-2011	2,816,928	0.7		0.7
Children	2009-2011	759,957	0		0
Farming/occupational	2006	212,508	0	34,001	34,001

National estimates based on studies with following study characteristics

Criteria	Farming/occupational population
Number of studies used for calculation	1
Review periods covered (first, second, both)	first
Sampling (representative, simple sample or unspecified)	simple sample
Diagnose (study scientists, self-reported)	self-reported
Latency period (24 h, 48 h, other, unspecified)	unspecified
At-risk time (annual, <1 y, other, ever, unspecified)	other
No. of symptoms (>=1, >=2, unspecified)	unspecified

30. Japan

Publications extracted for synthesis 2006-2018: 0

Publications extracted for synthesis 2019-2023: 1

Eddleston et al. 2022 (95)

- Period of study = 2019
- Based on national statistics
- 221 deaths from pesticide poisonings, 75 of which were unintentional deaths. Non-fatal poisonings were not reported in this publication

WHO Mortality data: On average, 44 fatal pesticide poisonings per year were reported in the WHO Mortality database for the general population of Japan (2017-2021). No fatal UAPP were reported for children and farming population.

Estimation of national figures

For the general population we used the data by Eddleston et al. as they were obtained from the Vital Statistics of Japan and are considered more complete than the WHO data.

Annual national estimates for Japan

Population type	Year	Population	Fatal	Non-fatal	Total cases
General	2019	126,633,000	75		75
Children	2017-2020	15,279,618	0		0
Farming/occupational	2017-2020	2,233,052	0		0

31. Kenya

Publications extracted for synthesis 2006-2018: 3

Mureithi et al. 2011 (96)

- Period of study = 2006-2007 data
- Area of study = Sagana, Central Highlands
- Sample = 140, representative, small farmers raising horticultural crops for commercial purposes
- Morbidity = 25% (35 cases), based on symptom of highest prevalence (breathing problems)
- Data ok to extrapolate

Macharia et al. 2015 (97)

- Period of study = 2005 and 2008
- Area of study = seven major vegetable producing districts of Central Province (Kiambu, Kirinyaga, Murang'a, Nyandarua, and Nyeri North) and Eastern Province (Makueni and Meru Central)
- Sample =363, representative, 70% male farmers in 2008
- Survey with self-reported symptoms
- Morbidity = 34% (124 cases)
- Data ok to extrapolate

Ngolo et al. 2018 (98)

- Period of study = May-August 2016
- Area of study = Ewaso Narok wetland, Laikipia County
- Sample = 86, simple sample, small-scale farmers, 87% male
- Morbidity = 46.5% (40 cases) for highest symptom prevalence (headache) after pesticide application
- Data ok to extrapolate

Publications extracted for synthesis 2019-2023: 1

Marete et al. 2021 (99)

- Period of study = March – May 2016
- Area of study = Meru County
- Sample = 173, farmers engaged in horticulture, 57% male, 26% female, 9% children
- Mortality = 26% (45 cases) farmers experiencing poisoning due to specific active ingredients
- At-risk timeframe of 3 years prior to the time of the survey
- Data not included because of 3-yr timeframe

WHO Mortality data: no mortality data were available.

Estimation of national figures

We base the extrapolation on the following studies arriving at an averaged percentage of 35.17 and an national estimate: 0.3517*5,399,851= 1,899,128

Study	Study year	Sample	Morbidity (%)
Macharia et al 2015	2008	363	34
Mureithi et al 2011	2006-2007	140	25
Ngolo et al 2018	2016	86	46.5
Average			*35.2*

National estimates for Kenya

Population type	Year	Population	Fatal	Non-fatal	Total cases
Farming/occupational	2006-2007,2008,2016	5,399,851		1,899,128	1,899,128

National estimates based on studies with following study characteristics

Criteria	Farming/occupational population
Number of studies used for calculation	3
Review periods covered (first, second, both)	first
Sampling (representative, simple sample or unspecified)	representative
Diagnose (study scientists, self-reported)	self-reported
Latency period (24 h, 48 h, other, unspecified)	24 h
At-risk time (annual, <1 y, other, ever, unspecified)	unspecified
No. of symptoms (>=1, >=2, unspecified)	unspecified

32. Kuwait

Publications extracted for synthesis 2006-2018: 1

Publications extracted for synthesis 2019-2023: 0

Jallow et al. 2017 (100)

- Period of study = not given, but post-2010
- Area of study = 2 major agricultural regions (Wafra and Abdally) which account for 90% of total crop production (dates, vegetables, cereals, pulses, and forages)
- Sample = 250; representative, reported as farmers only, but likely to be workers also as the majority of farms are operated by expatriate labours from India and North Africa (n=250)
- Self-reported symptoms
- Morbidity = 82% (205 cases) having at least one symptom
- Data ok to extrapolate

WHO Mortality Data: available for 2017-2019: General population = 1.3 cases annually; children =1; farming/occupational = 0

Estimation of national figures

We used the Jallow et al prevalence and the size of the farming/occupational population for 2014.

Annual national estimates for Kuwait

Population type	Year	Size	Fatal	Non-fatal	Total
General	2017-2019	4,294,396	1.3		1.3
Children	2017-2019	908,396	1		1
Farming/occupational	2014	67,233	0	55,131	55,131

National estimates based on studies with following study characteristics

Criteria	Farming/occupational population
Number of studies used for calculation	1
Review periods covered (first, second, both)	first
Sampling (representative, simple sample or unspecified)	representative
Diagnose (study scientists, self-reported)	self-reported
Latency period (24 h, 48 h, other, unspecified)	other
At-risk time (annual, <1 y, other, ever, unspecified)	annual
No. of symptoms (>=1, >=2, unspecified)	>=1

33. Lao DPR

Publications extracted for synthesis, 2006-2018: 1

Schreinemachers et al. (35)

- Period of study =2015
- Area of study = not specified
- Farmers only, only those growing yard-long bean and leaf mustard
- Sample =300, representative, about 38% female
- Self-reported symptoms
- Morbidity = 39% (117 cases), for highest symptom only
- Data ok to extrapolate

Publications extracted for synthesis, 2019-2023: 1

Shattuck 2021 (101)

- Period of study = 2017-2018
- Area of study = Kham District of Xieng Khouang Province in Northeastern Laos – a center of both the smallholder maize boom
- Sample =86, representative, farmers, all male except for one female
- Morbidity = 92% (79 cases), one or more symptoms
- Data ok to extrapolate

WHO Mortality data: no mortality data were available.

Estimation of national figures

For the national estimation, we used an average prevalence of 66 % and an average farming population (2015, 2017, 2018) of 1,760,134.

Annual national estimates for Laos

Population type	Year	Population size	Fatal	Non-fatal	Total cases
Farming/occupational	2015,2017,2018	1,760,134		1,161,688	1,161,688

National estimates based on studies with following study characteristics

Criteria	Farming/occupational population
Number of studies used for calculation	2
Review periods covered (first, second, both)	both
Sampling (representative, simple sample or unspecified)	representative sample
Diagnose (study scientists, self-reported)	self-reported
Latency period (24 h, 48 h, other, unspecified)	Other
At-risk time (annual, <1 y, other, ever, unspecified)	annual
No. of symptoms (>=1, >=2, unspecified)	>=1

34. Lebanon

Publications extracted for synthesis 2019-2023: 0

Publications extracted for synthesis 2019-2023: 1

Abou Ibrahim et al. 2023 (102)

- Period of study = November 2021 to October 2022
- Area of study = 3 provinces (Mount Lebanon, Nabatiyeh, and Beqaa)
- Sample = 104, cross-sectional using a representative sample of farmers, approximately 1:1 male to female
- It was confirmed by personal communication with the lead author that the data refer to "annual" poisonings, however this is not described in the publication itself
- Morbidity = 38.1% (40 cases), most frequently reported symptom of extreme fatigue
- Data ok to extrapolate

WHO Mortality data: On average, 3.2 fatal pesticide poisonings per year were reported in the WHO Cause of Death database for the general population of Lebanon (2017-2021). In addition, 0.2 fatal pesticide poisonings per year were reported for the same period for children. No fatal UAPP was reported for the farming population.

Annual national estimates for Lebanon

Data for children and general population based on WHO data.

Population type	Year	Population size	Fatal	Non-fatal	Total cases
General	2017-2021	5,819,510	3.2		3.2
Children	2017-2021	1,586,112	0.2		0.2
Farming/occupational	2022	63,820	0	24,315	24,315

National estimates based on studies with following study characteristics

Criteria	Farming/occupational
Number of studies used for calculation	1
Review periods covered (first, second, both)	second
Sampling (representative, simple sample or unspecified)	representative
Diagnose (study scientists, self-reported)	self-reported
Latency period (24 h, 48 h, other, unspecified)	unspecified
At-risk time (annual, <1 y, other, ever, unspecified)	annual
No. of symptoms (>=1, >=2, unspecified)	>=1

35. Malawi

Publications extracted for synthesis 2006-2018: 1

Publications extracted for synthesis 2019-2023: 0

Kasambala Donga and Eklo 2018 (103)

- Period of study = June 2015-January 2016
- Area of study = Nkhata Bay, Nkhotakota and Chikwawa Districts
- Sample = 55, representative, sugarcane farmers
- Morbidity = 78% (43 cases) based on most prevalent symptom, skin irritation
- Data ok to extract

WHO Mortality data: no mortality data were available.

Estimation of national figures

0.78 * 6,075,069 farmers in Malawi = 4,738,554

National estimates for Malawi

Population type	Year	Population	Fatal	Non-fatal	Total cases
Farming/occupational	2015-2016	6,075,069		4,738,554	4,738,554

National estimates based on studies with following study characteristics

Criteria	Farming/occupational
Number of studies used for calculation	1
Review periods covered (first, second, both)	first
Sampling (representative, simple sample or unspecified)	representative
Diagnose (study scientists, self-reported)	self-reported
Latency period (24 h, 48 h, other, unspecified)	unspecified
At-risk time (annual, <1 y, other, ever, unspecified)	unspecified
No. of symptoms (>=1, >=2, unspecified)	unspecified

Limitations

The study surveyed sugar cane farmers. The study authors report that sugarcane is the second most valuable crop after tobacco, but a 2015 FAO report does not have data on the number of sugarcane farmers—possibly because the farming situation is complex, as large estates make up 84% of those growing sugarcane, with the rest of the crop grown by smallholder farmers. (FAO. Review of Food and Agricultural Policies in Malawi: Country report 2014. Rome: Food and Agriculture Organization of the United Nations; 2015. Available from: http://www.fao.org/3/a-i4675e.pdf)

36. Malaysia

Publications extracted for synthesis 2002-2018: 3

All studies were from the National Poisons Control Centre, giving national figures. However, one paper studied the herbicide paraquat only, and one paper focused on children only.

Nur et al. 2012 (104)

- Period of study = 2006-2009
- Cases reported to the National Poisons Centre of Malaysia
- Children only
- Morbidity = 85% (93.5 cases)
- Likely to be an underestimation as 60.1% of cases were toddlers and no suicides reported in this group

Leong et al. 2018 (105)

- Period of study = 2004-2015
- Paraquat only
- Paraquat poisoning calls to the Malaysia National Poison Centre following its ban and subsequent restriction
- Morbidity = 35 cases per year, but may include mortality

Tangiisuran et al. 2018 (106)

- Period of study = 2006-2015
- Evaluation of poisoning exposure calls managed by the Malaysia National Poison Centre
- Morbidity = 420 cases per year

Publications extracted for synthesis 2019-2023: 3

Ahmad et al. 2023 (107)

- Period of study = Sept 2018-Feb 2019
- Area of study = Bachok and Kota Bharu districts, Kelantan state
- Sample = 150 farmers
- Morbidity = 32.67% (49 cases), at least one symptom reported within 48 hrs of spraying
- Data ok to extract

Kamaruzaman et al. 2020 (108)

- Period of study = 2006-2015
- National Poisons Centre register: calls made by health care professionals from hospitals only
- Morbidity = 0.00001394% (420 cases)
- Not extrapolated: hospital cases only that resulted in a call to National Poisons register so not representative of UAPP

Sulaiman et al. 2019 (109)

- Period covered = unknown
- Area of study = Sabah district
- Sample = 270, palm oil plantation workers

- Symptoms occurred during the course of spraying; those who were not sure if the symptoms came from the pesticides were not included
- Morbidity = 54.81% (148 cases)
- Data ok to extrapolate

Summary of studies 2019-2023

Study	Study year	Sample	Morbidity (%)
Ahmad et al 2023	2018-2019	150	32.7
Sulaiman et al, 2019	unknown	270	54.8
Average			*43.7*

WHO Mortality data available from 2017 to 2020: general population = 0.5 cases/yr; children = 0 cases; farmers = not recorded

Estimation of national figures

For the farming/occupational population we averaged prevalence and averaged the population size. For the general population we excluded the Leong et al paper on paraquat from the synthesis as it covers only one pesticide and is rendered unnecessary by the other papers.

Annual national estimates for Malaysia

Population type	years	Population	Fatal	Non-fatal	Total cases
General	2017-2020	32,393,032	0.5		0.5
	2006-2015	28,148,720		420	420.5
Children	2017-2020	7,756,357	0		0
	2006-2009	7,807,418		93.5	93.5
Farmers/occupational	2017-2020	1,722,158		753,272	753,272

National estimates based on studies with following study characteristics

Criteria	Farming/occupational population
Number of studies used for calculation	2
Review periods covered (first, second, both)	second
Sampling (representative, simple sample or unspecified)	representative
Diagnose (study scientists, self-reported)	self-reported
Latency period (24 h, 48 h, other, unspecified)	48 h
At-risk time (annual, <1 y, other, ever, unspecified)	annual
No. of symptoms (>=1, >=2, unspecified)	>=1

37. Mexico

Publications extracted for synthesis 2006-2018: 1

Gonzales-Santiago et al. 2017 (110)

- Period of study = 2000-2012 dealing with unintentional and self-poisoning mortalities
- The supplementary tables contain national data on a yearly basis using ICD-10 codes. Table S2 provides data for unintentional poisoning and Table S3 the population data. From this it was possible to derive data for unintentional pesticide poisoning for 2006 to 2011 and calculate the annual average for the general population and for children
- The paper contains fatal poisoning data, but not population data for this year. These fatalities data were older than those provided by WHO and, therefore, were not used

Publications extracted for synthesis 2019-2023: 2

Bernardino-Hernández et al. 2019 (111)

- Period of study = 2011-2012
- Area of study = three different municipalities (with distinct cultivation characteristics) in the Highlands of Chiapas
- Sample = 523, peasant farmers cultivating different products (149 flower producers, 116 vegetable growers, 258 corn producers)
- Self-reported symptoms after pesticide application
- Morbidity = 62.7% (328 cases). The percentage of those who experienced intoxications ranged from 77.9% (floriculturists, municipality of Zinacantán) through 65.5% (horticulturists, municipality of Chamula) to 52.7% (corn growers, municipality of Amatenango del Valle)
- Data ok to extrapolate

Esquivel-Valenzuela et al. 2019 (112)

- Period of study = 2015
- Area of study = border region of the states of Coahuila and Durango
- Sample = 90, study of practices and risks of pesticide use of horticultural farm workers
- None used personal protective equipment (PPE), 25% had PPE available, but did not use it because it was too uncomfortable
- Morbidity = 34% (31 cases)
- The authors did not specify the at risk time, stating that “three of ten” of these persons suffered “at least once” from a pesticide intoxication
- Data ok to extrapolate

WHO Mortality data: available from 2017-2021 reporting 108.2 annual cases for the general population, 15.6. for children and 3.8 for the farming population

Estimation of national figures

For the farming/occupational population we averaged the study results of Bernardino-Hernández et al. 2019 (62.7%) and Esquivel-Valenzuela et al. 2019 (34%) arriving at a non-fatal UAPP prevalence of 48%.

Annual national estimates for Mexico

Population type	Year	Population	Fatal	Non-fatal	Total cases
General	2017-2021	124,928,374	108.2		108.2
Children	2017-2021	32,194,859	15.6		15.6
Farming/occupational	2017-2021	7,333,626	3.8		
	2011-2012, 2015	6,891,822		3,308,075	3,308,078.8

National estimates based on studies with following study characteristics

Criteria	Farming/occupational population
Number of studies used for calculation	2
Review periods covered (first, second, both)	second
Sampling (representative, simple sample or unspecified)	representative
Diagnose (study scientists, self-reported)	self-reported
Latency period (24 h, 48 h, other, unspecified)	other
At-risk time (annual, <1 y, other, ever, unspecified)	unspecified
No. of symptoms (>=1, >=2, unspecified)	unspecified

38. Moldova

Publications extracted for synthesis 2006-2018: 1

Publications extracted for synthesis 2019-20123 0

Pinzaru et al. (113)

- Period of study = 2011-2015
- Reviewed records from local registers and a reporting mechanism for states in Moldova for the general population, for chemical poisonings
- On average 142 pesticide poisonings were reported with 10 annualized fatalities. Around 90% of the total 52 reported fatal cases were suicides with no information on the share of suicides among the non-fatal cases

WHO Mortality data: the most recent data were from 2017-2018 giving annually 1 case for the general population, 0.5 for children and no fatalities for the farming population.

Estimation of national figures

For fatalities we used the more recent WHO data. For non-fatal UAPP in the general population, we used the averaged cases per year of 142. For children from 2011 to 2015, there were 35.3 non-fatal poisoning cases per year.

National estimates for Moldova

Population type	Year	Population	Fatal	Non-fatal	Total cases
General	2011-2015	3,557,715		142	
	2017-2018	2,731,196	1		143
Children (1-18 years)	2011-2015	566,948		35.3	
Children	2017-2018	521,412	0.5		35.8
Farming/occupational	2017-2018	886,499	0		0

Limitations

For fatal poisonings, Pinzaru et al. 2017 stated that 90% were suicides. However, it was not clear if there were any intentional cases among the non-fatal poisonings recorded, though it is highly likely that there would be less intentional poisonings among the non-fatal cases. In calculating the non-fatal cases of pesticide poisoning per year, the assumption was made that all of the cases were accidental. In discussing the non-fatal cases, Pinzaru et al. 2017 stated: “Lately, frequent cases of accidental poisonings with pesticides were registered in Republic of Moldova, particularly within the pre-university education institutions” and: “In the past years, it was registered an increasing number of cases of lethal chemical poisoning both for adults and children, which occurred accidentally, or as suicides (defined as intentional self-poisoning).”

39. Morocco

Publications extracted for synthesis 2006-2018: 2

Publications extracted for synthesis 2019-2023: 0

Imane et al. 2016 (114)

- Period of study = 2008
- Area of study = 15 rural communes in the district of Meknes El Hajeb in the north-east
- Sample =402, cross-sectional study of farmers, mostly engaged in cereals, fodder, potatoes, onions, vineyards, orchards
- Self-reported symptoms
- Morbidity = 20.6% (83 cases)
- 80.5% don't wear protective mask, only 6.3% wore impervious gloves every time
- 35.3% are illiterate
- Data ok to extrapolate

Tomenson and Matthews 2009 (4)

- Period of study = 2006
- Survey conducted by market research company for Syngenta, across many countries targeting smallholder knapsack sprayers, in regions of moderate to very intensive use of pesticides, users said to be at highest risk of exposure
- Sample = 250; 62% were farm owners, <1% contractors so the rest are presumably workers
- Morbidity = 85.2% (213 cases) in past 12 months – including 8 'serious' cases (hospitalisation), 47 'moderate' (requiring trained medical assistance) cases and the rest regarded as 'minor'
- Data ok to extrapolate

WHO Mortality data: used the data available for the period from 2010-2014 because there were no recent data available.

Estimation of national figures

Both studies are of a similar period, slightly preceding the WHO mortality data. One claims to be of users at highest risk and can be assumed to be an upper limit; the other doesn't make any claim about exposure, so the prevalence was averaged without weighting.

20.6% + 85.2% / 2 = 52.9%

This figure was then applied to the farming population averaged over 2006, 2008.

National estimates for Morocco

Population type	Year	Population	Fatal	Non-fatal	Total cases
General	2010-2014	33,349,020	14.6		14.6
Children	2010-2014	9,363,000	0.8		0.8
Farming/occupational	2006,2008	4,191,748	0	2,217,435	2,217,435

National estimates based on studies with following study characteristics

Criteria	Farming/occupational population
Number of studies used for calculation	2
Review periods covered (first, second, both)	first
Sampling (representative, simple sample or unspecified)	representative sample
Diagnose (study scientists, self-reported)	self-reported
Latency period (24 h, 48 h, other, unspecified)	unspecified
At-risk time (annual, <1 y, other, ever, unspecified)	annual
No. of symptoms (>=1, >=2, unspecified)	>=1

40. Nepal

Publications extracted for synthesis 2006-2018: 4

- Neupane et al. 2014 (115) Period of study = 2012
- Area of study = Chitwan
- Sample = 90, representative, commercial vegetable growers, all male
- The authors reported the prevalence of symptoms based on a list derived from of the WHO classification tools on clinical symptoms of acute organophosphate and carbamates poisoning
- In addition to asking “Did you suffer from any of the following symptoms in the last month?” they recorded whether these symptoms were recognized “immediately after spraying pesticide”. However, for the latter the authors gave only the lifetime prevalence
- Data not used for extrapolation because of the ‘ever’ prevalence

Singh et al. 2015 (116)

- Period of study = unknown
- Area of study = Haldibari, Danabari and Kanyam tea estates of Nepal
- Sample = 262, cross-sectional study of tea workers
- Morbidity = 34 % (90 cases), for most frequent symptom (eye irritation)
- The paper is a short communication only. No details on the sample, the questionnaire and the study year were given; no assessment of the study quality could be made. Tea cultivation seems not to have a larger share in Nepal’s agriculture. Given these restrictions we do not use this paper for estimation
- Data not ok to extrapolate

Gyenwali et al. 2017 (117)

- Period of study = 2015
- Area of study = Chitwan which according to the authors is one of 75 districts in Nepal and is inhabited by 2.2% of Nepal’s population. It is located in the south-central part of the country and known for its commercial vegetable farming where pesticides are used extensively
- Study basis is four major hospitals which were purposively selected as people from Chitwan mostly visit these hospitals for all medical emergencies
- The authors state the population at risk and calculate the annual incidence rates of unintentional pesticide poisoning in Chitwan district of 6.99 per 100,000 population derived from 44 hospital admissions in 2015. The cases likely include fatalities which were not differentiated for non-intentional poisoning
- Overall fatality ratio was 3.8 %
- Data ok to extrapolate

Bhandari et al. 2018 (118)

- Period of study = not given but presumed to be after 2008 as their research model is based on a study published in 2008
- Area of study = Gaidahawa Rural Municipality in the Rupandehi district of Nepal. The population of the municipality was stated as 47,565 individuals. The Gaidahawa Rural Municipality is comprised of nine wards – the smallest administrative unit. The study selected

wards where the majority of the farmers are engaged in commercial vegetable farming. Each ward is comprised of a number of villages

- Sample =183, random, 10 % of households, 89.6% male
- Morbidity = 78% (for most prevalent symptom of headache); "almost all" farmers perceived acute health symptoms after pesticide application
- Data ok to extrapolate

Publications extracted for synthesis 2019-2023: 1

Bhattarai et al 2021 (119)

- Period of study = 2013-2014
- Area of study = Kavrepalanchowk, Nawalparasi, Ilam and Rasuwa districts
- Sample = 628, vegetable farmers, 64.2% male
- Cross sectional study which made use of data from a study conducted by the Nepal Health Research Council Health Effects of Pesticide, collected by a face-to-face interview
- The study aimed to analyse acetylcholinesterase levels with respect to self-reported acute health symptoms among vegetable farmers
- Morbidity = 50.5 % complaining about discomfort "immediately after spraying pesticides"
- Data ok to extrapolate

WHO Mortality data: no mortality data were available.

Estimation of national figures

Based on the above-mentioned studies the situation of pesticide poisoning in vegetable farming seems to be reasonably well addressed. According to FAO in Nepal 66 % of the population is directly engaged in farming which is mostly subsistence (http://www.fao.org/nepal/fao-in-nepal/nepal-at-a-glance/en/). We averaged World Bank population data for the years of the studies 2014, 2021 to amount to 28,822,657. We calculated the farming population from this average: 28,822,657 x 66% = 19,022,954.

Given that a newer study is available, we excluded the study by Bhandari et al. for the estimation of national figures because there is no information on the at-risk times. We also excluded the study by Neupane et al. 2014 because of the 'ever' prevalence. The prevalence of 50.5% by Bhattarai et al 2021 is used to estimate annual national figures as 0.51 x 19,022,954 = 9,701,707 cases within the farming population.

For hospital admissions we assume that the situation in Chitwan can be transferred to the whole country giving 6.99 x 286/100.000 = 2,003 nation-wide cases.

Annual National estimates for Nepal

Population type	Year	Population	Fatal	Non-fatal	Total cases
General	2015	28,656,282			2,003
Farming/occupational	2014,2021	19,022,954		9,701,707	9,701,707

National estimates based on studies with following study characteristics

Criteria	Farming/occupational population
Number of studies used for calculation	1
Review periods covered (first, second, both)	second
Sampling (representative, simple sample or unspecified)	representative sample
Diagnose (study scientists, self-reported)	self-reported
Latency period (24 h, 48 h, other, unspecified)	other
A-risk time (annual, <1 y, other, ever, unspecified)	unspecified
No. of symptoms (>=1, >=2, unspecified)	unspecified

41. Nigeria

Publications extracted for synthesis 2006-2018: 3

Oluwole and Cheke 2009 (120)

- Period of study = March 2008
- Area of Study = Ekiti state, major crop in the area is rice followed by cash crops (cocoa, kola nut), horticultural crops (vegetables, fruits) and cowpea
- Sample =150; representative, 93% male farmers
- Symptoms reported within 48 hours of pesticide spraying
- Morbidity = 91.3% based on the most prevalent symptom (eye irritation)
- Data ok to extrapolate

Ugwu et al. 2015 (121)

- Period of study = 2014
- Area of study = Oyo State
- Sample =150; representative, 122 male and 28 female vegetable farmers
- Morbidity = 74% (111 cases), for at least one health symptom from pesticide handling
- Data ok to extrapolate

Bassi et al. 2016 (122)

- Period of study = data were assumed to have been collected in 2006 or later based on the publication date and the 2006 census data used in the study
- Area of study = Fadan Daji district of the Kaduna state, described as a major farming area
- Sample = 250; representative, 154 male and 96 female farmers
- Self-reported data
- Morbidity = 42% (106 cases) based on the most prevalent symptom (cough)
- Data ok to extrapolate

Publications extracted for synthesis 2019-2023: 1

Adedire et al. 2023 (123)

- Period of study = not reported, assumption made that the study was conducted after 2006.
- Area of study = Idanre in southwest Nigeria
- Sample =150; 131 male, 19 female cocoa farmers
- Morbidity = 68% (102 cases) farmers reporting symptoms
- At-risk timeframe = 1 year
- Farmers (52%) reported applying pesticides more than 4 times in a year to their farms
- Data ok to extrapolate

WHO Mortality data: no mortality data were available.

Estimation of national figures

The farming population was averaged for the most recent 5 years (2012-2016). The World Bank data for the 2021 Nigerian farming population was used. The prevalence was averaged for the four studies was 69 %:

0.69*23,267,885=16,054,841

National estimates for Nigeria

Population type	Year	Population	Fatal	Non-fatal	Total cases
Farming/occupational	2008, 2012-2016, 2014; 2021	23,267,885		16,054,841	16,054,841

National estimates based on studies with following study characteristics

Criteria	Farming/occupational population
Number of studies used for calculation	4
Review periods covered (first, second, both)	both
Sampling (representative, simple sample or unspecified)	representative sample
Diagnose (study scientists, self-reported)	self-reported
Latency period (24 h, 48 h, other, unspecified)	48 h
At-risk time (annual, <1 y, other, ever, unspecified)	annual
No. of symptoms (>=1, >=2, unspecified)	>=1

42. Pakistan

Publications extracted for synthesis 2006-2018: 3

Kouser and Qaim 2013 (124)

- Period of study = Dec 2010-Feb 2011, immediately following cotton harvest
- Area of study = four major cotton producing districts (Vehari, Bahawalnagar, Bahawalpur, Rahimyar Khan) in Punjab, which has 80% of the cotton crop, so extrapolation is sound
- Sample = 352, cotton farmers, representative sample: 248 were Bt (or partial Bt) growers and 104 non-Bt growers
- Bt varieties reached 5.9 million acres in 2010, which is 75% of Pakistan's total cotton acreage
- Morbidity = 40.3% (142 cases)
- Data ok to extrapolate

Tahir and Anwar 2012 (125)

- Period of study = date not given, but ≤ 2012
- Area of study = Multan and Bahawalpur Divisions, the major cotton growing areas of Pakistan
- Sample = 30, women cotton pickers
- Morbidity = 100% suffering headache, vomiting and nausea
- Weak study, small sample size, selection of participants not explained
- Not extrapolated

Bakhsh et al. 2016 (126)

- Period of study = 2010
- Area of study = 10 villages in Vehari district, southern Punjab
- Sample = 270; representative, women cotton pickers, study compares BT cotton to non-BT cotton
- Cotton picking is solely done by women in Pakistan – about 0.1 million women, highly exposed to residual pesticides; Vehari = 9% of cotton production in Punjab, so extrapolation to all cotton pickers is sound
- Morbidity = 61% (167 cases); Bt cotton pickers reported one or more health effects of pesticide during picking season, whereas this percentage for non-Bt cotton households was 66 %; for 2 or more symptoms 35%/38% (BT/non-BT); highest symptom occurrence in BT = 23% (headache), non-BT = 58% (headache)
- Bt cotton and non-BT cotton morbidity were averaged, = 61.8%
- Data ok to extrapolate

Publications extracted for synthesis 2018-2023: 3

Elahi et al. 2019 (127)

- Period of study = 2017 (6 months)
- Area of study = three zones of Punjabi province, upper, central, and lower zone; selected Attock, Gujranwala, and Sargodha from the upper zone; Bhakkar, Faisalabad, and Jhang from the middle zone and D.G Khan, Multan, and Bahawalnar from the lower zone, purposively
- Sample =360, representative, rice farmers only

- Symptoms immediately after spraying
- Morbidity = 41.7% (150 cases)
- Data ok to extrapolate

Memon et al. 2019 (128)

- Period of study = 2017
- Sample = 260, female cotton pickers, from Shaheed Benazirabad District of Sindh Province
- Morbidity = 30% (78 cases) during cotton picking
- Data ok to extrapolate

Khan et al. 2022 (129)

- Period of study = 2020
- Sample = 332; farmers only, 99% male, simple sample, from Matli, Badin in the Sindh province
- Morbidity = 91.6% (304 cases), for highest symptom (headache)
- Data ok to extrapolate

WHO Mortality data: no mortality data were available.

Estimate of national figures

Whereas in the first review period there were only cotton farmers and pickers, the second review period added two studies on UAPP in general farming. We think that taken together the studies give a valid picture on UAPP in the country. Therefore, we averaged the results of all five eligible studies arriving at an UAPP prevalence of 53% for an averaged farming population in the respective years of 26,825,439.

Annual national estimates for Pakistan

Population type	Year	Population size	Fatal	Non-fatal	Total cases
Farming/occupational	2010,2019,2022	26,825,439		14,217,483	14,217,483

National estimates based on studies with following study characteristics

**Criteria**	**Farming/occupational population**
Number of studies used for calculation	5
Review periods covered (first, second, both)	both
Sampling (representative, simple sample or unspecified)	representative sample
Diagnose (study scientists, self-reported)	self-reported
Latency period (24 h, 48 h, other, unspecified)	other
At-risk time (annual, <1 y, other, ever, unspecified)	annual
No. of symptoms (>=1, >=2, unspecified)	>=1

43. Palestine

Publications extracted for synthesis 2006-2018: 3

Publications extracted for synthesis 2019-2023: 0

Zyoud et al. 2010 (130)

- Period of study = 2008
- Area of study = Nablus district, West Bank; the villages of Tammun, Tubas, EL-Far'a, Tayasir, and Nassariyah, 20 km north-east of Nablus city. These five regions are characterized by important agricultural activity (potatoes, onions, carrots, tomatoes, cucumbers, bitter gourds, cabbage, and cauliflowers)
- Sample = 381, farm workers who work in open fields or greenhouses or both, 97.9% male
- Morbidity = 37.5% (143 cases), for most prevalent symptom
- Data ok to extrapolate

Yasser 2016 (131)

- Date of survey unknown
- Area of study = Gaza
- Sample = 139, farmers and workers from 57 greenhouses, 70% male
- Morbidity = 18.7% (26 cases), for most prevalent symptom
- Data ok to extrapolate

El-Nahhal 2017 (132)

- Period of study = 2015-16
- Area of study = 4 locations in Gaza
- Sample = 169, farmers and workers from 67 greenhouses, 70% male
- Morbidity = 47.3% (80 cases), for most prevalent symptom
- Data ok to extrapolate

WHO Mortality data: for Occupied Palestinian Territory: 0 fatalities for 2017-2018.

Estimation of national figures

The 3 studies on farmers and workers were averaged to arrive at occupational morbidity of 34.5%.

Study	Study year	Sample	Morbidity (%)
El-Nahhal 2017	2015-6	169	47.3
Yasser 2016	?	139	18.7
Zyoud 2010	2008	381	37.5
Average			*34.5*

Annual National estimates for Palestine

Population type	Year	Population size	Fatal	Non-fatal	Total
General	2018	2,921,170	0		0
Children	2018	1,072,100	0		0
Farming/occupational	2010, 2016	81,489		28,113	28,113

National estimates based on studies with following study characteristics

Criteria	Farming/occupational population
Number of studies used for calculation	3
Review periods covered (first, second, both)	first
Sampling (representative, simple sample or unspecified)	representative sample
Diagnose (study scientists, self-reported)	self-reported
Latency period (24 h, 48 h, other, unspecified)	other
At-risk time (annual, <1 y, other, ever, unspecified)	unspecified
No. of symptoms (>=1, >=2, unspecified)	>=1

44. Philippines

Publications extracted for synthesis 2006-2018: 4

Publications extracted for synthesis 2019-2023: 0

Lu 2011 (133)

- Period of study = not stated, assumption made that study year likely 2006 or later
- Area of study = Benguet, the largest vegetable producing region in the northern part of Luzon, major crops = tubers, roots, bulbs, leafy vegetables, stems and flowers
- Sample = 400 vegetable farmers, 51.8% male
- Morbidity = 48% (192 cases)
- Data ok to extrapolate

Lu and Cosca 2011 (134)

- Period of study = not stated, assumption made that study year likely 2006 or later
- Area of study = 6 communities in Benguet
- Sample = 542, representative, vegetable farmers, 53.7% male
- Morbidity = 51% (276 cases)
- Data ok to extrapolate

Perez et al. 2015 (135)

- Period of study = 2014, Aug-Sept
- Southern Mindanao: the Municipality of Baungon and City of Valencia in Bukidnon Province; Municipalities of Alubijid and El Salvador in Misamis Oriental; Municipality of Panaon and City of Oroquieta in Misamis Occidental; Municipalities of Tukuran and Molave in Zamboanga del Sur
- Sample = 528 farmers, growing rice, corn, cassava, 97.8% male
- Morbidity = 32.95% (174 cases), most prevalent symptom of skin irritation
- Data ok to extrapolate

Del Prado-Lu 2015 (136)

- Period of study = not stated
- Area of study = 26 farms selected from 6 villages (barangays) in Sta. Maria, Pangasinan
- Sample = 68, representative, farmers and workers growing eggplant
- Morbidity = 100%
- Medical attention not sought
- Data ok to extrapolate

WHO Mortality data: data available for 2017-2019 35.33 annualized fatalities occurred in the general population, 2 in children. No data were available for the farming population.

Estimation of national figures

Study	Study year	Sample	Morbidity (%)
Del-Prado Lu 2015	not stated	58	100
Lu 2011	not stated	400	48
Lu & Cosca 2011	2008	542	51
Perez et al. 2015	2014	528	33
Average			*58*

The 4 studies on farmers and workers were averaged to arrive at a percentage occupational morbidity of 57.99%.

Because three of the four studies do not have dates but are likely to be towards the earlier time threshold of 2006, we used the farming population average of 2007-2014 of 12,467,311.

Annual National estimates for Philippines

Population type	Year (average)	Population size	Fatal	Non-fatal (estimated)	Total cases
General	2017-2019	108,562,714	35.3		35.3
Children	2017-2019	34,299,278	2		2
Farming/occupational	2007-2014	12,467,311		7,229,794	

National estimates based on studies with following study characteristics

Criteria	Farming/occupational population
Number of studies used for calculation	4
Review periods covered (first, second, both)	first
Sampling (representative, simple sample or unspecified)	representative sample
Diagnose (study scientists, self-reported)	study scientist
Latency period (24 h, 48 h, other, unspecified)	other
At-risk time (annual, <1 y, other, ever, unspecified)	unspecified
No. of symptoms (>=1, >=2, unspecified)	unspecified

45. Portugal

Publications extracted for synthesis 2006-2018: 1

Publications extracted for synthesis 2019-2023: 0

Tomenson and Matthews 2009 (4):

- Period of study = 2006
- Survey of pesticide applicators and farmers commissioned by Syngenta
- Sample = 250, representative, farmers and pesticide users reporting pesticide poisoning incidents, serious, moderate and minor
- Morbidity = 34% (85 cases) in the past 12 months

WHO Mortality data: Data from 2017 to 2019 were available, with 5 annual fatal UAPP for the general population and 0 for children and the farming population respectively.

Estimation of national figures

The farming/occupational population was taken for 2006, so 608,246 * 0.34 =206,804.

Table: National estimates for Portugal

Population type	Year	Population size	Fatal	Non-fatal	Total cases
General	2017-2019	10,290,128	5		5
Children, ages 0-14	2017-2019	1,416,274	0		0
Farming/occupational	2006	608,246		206,804	
	2017-2019	312,787	0		206,804

National estimates based on studies with following study characteristics

Criteria	Farming/occupational population
Number of studies used for calculation	1
Review periods covered (first, second, both)	first
Sampling (representative, simple sample or unspecified)	representative
Diagnose (study scientists, self-reported)	self-reported
Latency period (24 h, 48 h, other, unspecified)	unspecified
At-risk time (annual, <1 y, other, ever, unspecified)	annual
No. of symptoms (>=1, >=2, unspecified)	unspecified

46. Saudi Arabia

Publications extracted for synthesis 2006-2018: 2

Publications extracted for synthesis 2019-2023: 0

Alzahrani et al. 2017 (137)

- Period of study = 2011-2015
- Based on a review of records of cases reported to the Jeddah Health Affairs Directorate
- Accidental, 28 non-fatal cases/yr in Jeddah only
- Fatalities could not be extracted as the figures included intentional poisonings as well as accidental
- Non-fatal data ok to extrapolate

Alnasser et al. 2018 (138)

- Period of study = 2006-2017
- Only aluminium phosphide poisoning
- National figures, poisonings reported to Ministry of Health
- Accidental and occupational
- Mortality = 1 per year
- Morbidity = 4.4 per yr
- Data not ok for extrapolation

WHO Mortality data: No fatal UAPP were reported for 2021-2022.

Estimation of national figures

No occupational data were available. Alzarhani et al. 2017's data was averaged 28 from 2011-2015, the share of Jeddah to Saudi Arabia's population was 12.3%. Jeddah's population data were available for 2017 as 4,076,000 and the population of Saudi Arabia in 2017 was 33,099,147.

Source: World Population Review (WPR). http://worldpopulationreview.com/cities/saudi-arabia/jeddah

Annual National estimates for Saudi Arabia

Population type	Year	Population size (av)	Fatal	Non-fatal	Total cases
General	2013-2015	30,895,726		227.46	227.46
	2021-2022	36,179,608	0		0
Children	2021-2022	9,429,469	0		0
Farming	2021-2022	457,360	0		0

47. Senegal

Publications extracted for synthesis 2006-2018: 1

Publications extracted for synthesis 2019-2023: 0

Tomenson and Matthews 2009 (4)

- Period of study = 2006
- Survey of pesticide applicators and farmers commissioned by Syngenta Crop Protection among 6,359 users in 24 countries
- Sample = 249, representative, farmers and pesticide users from Senegal and pesticide poisoning incidents, serious moderate and minor
- Morbidity = 30.522% (76 cases) experiencing symptoms in the past 12 months

WHO Mortality data: No data were available.

Estimation of national figures

The farmer population was taken for 2006.

The percent prevalence was 30.522% * 1,198,448 = 365,790

National estimates for Senegal

Population type	Year	Population	Fatal	Non-fatal	Total cases
Farming/occupational	2006	1,198,448		365,790	365,790

National estimates based on studies with following study characteristics

Criteria	Farming/occupational population
Number of studies used for calculation	1
Review periods covered (first, second, both)	first
Sampling (representative, simple sample or unspecified)	representative
Diagnose (study scientists, self-reported)	self-reported
Latency period (24 h, 48 h, other, unspecified)	unspecified
At-risk time (annual, <1 y, other, ever, unspecified)	annual
No. of symptoms (>=1, >=2, unspecified)	unspecified

48. Serbia

Publications extracted for synthesis 2006-2018: 1

Publications extracted for synthesis 2018-2023: 0

Vucinic et al. (139)

- Period of study = 1998-2014
- Data from the National Poison Control Centre in Serbia. Non-fatal accidental poisonings data were available; the mortality data did not include information on whether the poisonings were intentional
- The non-fatal poisoning rate was reported as not having diminished during those years
- 410 cases were reported as being from organophosphate pesticide poisonings, with 8% of those cases due to accidental ingestion or inhalation

WHO Mortality data were available for the period 2017-2022, except for the farmer population.

Estimation of national figures

The average population of the last 5 years reported in the study (2010-2014) were used for the non-fatal poisoning prevalence. The non-fatal poisonings cases were reported in Vucinic et al. 2018 as: 410 cases * 8% accidental, divided by 17 years = 2 cases per year for the time period. As the rate had not changed according to authors, this was used as the number of cases per year for the Serbian general population.

National estimates for Serbia

Population type	Year	Population	Fatal	Non-fatal	Total cases
General	2010-2014	7,184,259		2	
General	2017-2022	6,891,100	1		3
Children	2017-2022	977,975	0		0

Limitations

The Vucinic study did not report data on poisonings from pesticides other than organophosphate insecticides and did not report these data by year for the general population. It was inferred based on the paper's discussion that non-fatal poisoning cases could be averaged per year. No data on fatalities were reported for the agricultural population.

49. South Africa

Publications extracted for synthesis 2006-2018: 1

Balme et al. 2010 (140)

- Period of study = 2006-2008
- Data on non-fatal and fatal pesticide poisonings of children from Red Cross War Memorial Children's Hospital, in Cape Town, South Africa
- Morbidity = 0.00482778% (67 cases)
- 1 fatality per year
- Data ok for extrapolation

Publications extracted for synthesis 2019-2023: 1

Balme and Stephen 2022 (141)

- Period of study = January 2009 to December 2019
- Retrospective review of non-fatal and fatal pesticide poisoning cases of children from who presented to the Red Cross War Memorial Children's Hospital, in Cape Town, South Africa
- Morbidity = 0.0017096% (18 cases)
- 0.5 fatalities per year
- Data ok for extrapolation

WHO Mortality data were available from 2017-2018 for the general (11.5 fatalities per year) and child population (8 per year). No farmer data were available.

Estimation of national figures

The catchment area described in Balme et al. 2010 for children aged 0-14 was 1,387,800. The eligible data were from 2006-2008, and the prevalence of non-fatal poisonings was taken as 0.00482778%. Balme et al. also reported 1 fatality per year.

The child population aged 0-14 from 2006-2008 was used to calculate the non-fatal pesticide poisonings estimate.

0.00482778% * 15,528,809 = 750

For Balme and Stephen 2022, the catchment area for the hospital had 1,042,259 for children aged 0-14. The prevalence from 2009 to 2019 was reported as 0.000048% for fatalities. The non-fatal poisonings prevalence and the child population from 2009-2019 were used to calculate the estimate.

0.0017096% * 15,863,085= 271

These numbers were averaged (=511) for the national estimate of nonfatal poisonings in children.

Both of the Balme et al. studies reported on fatalities, however, the WHO cause-of-death data were more recent and based on national level reporting, so the WHO data were used for the national estimate.

National estimates for South Africa

Population type	Year	Population	Fatal	Non-fatal	Total cases
General	2017-2018	56,990,422	11.5		11.5
Children	2006-2008, 2009-2019	16,011,149		511	511
Children	2017-2018	16,318,697	8		8

Limitations

It was not clear if the catchment area covered by the hospital in Cape Town in the Balme et al. studies was representative of other hospital areas, and the estimate was based only on the prevalence of poisoning from the hospital.

50. South Korea

Publications extracted for synthesis 2006-2018: 10

Kim et al. 2012 (142)

- Period of study = 2004-2006
- Hospital discharges provided rates of fatal and all APP per 100,000 populations; these were extrapolated by the authors from a representative sample of national hospitals
- However, rates were not differentiated for subpopulations for accidental poisoning, but the ratio of UAPP to all cases was provided as 15.5% (138 cases of 891)
- This share leads to a rate for fatal UAPP of 0.589 per 100,000 and 2.29 per 100,000 for UAPP for the general population
- Data ok to extrapolate

Lee et al. 2012 (143)

- Period of study = 2011
- Sample = 1,985, male farmers
- Cross-sectional survey in which respondents were asked whether any of 21 listed symptoms had been experienced within 48 hr of using pesticides
- Mortality = 23% (449 cases), reported experiencing at least one symptom
- Authors calculated an annual figure of 209,512 occupational cases nation-wide
- Data ok to extrapolate

Kim et al 2013 (144) same study as Lee et al. 2012

Kim et al. 2013 (145) same study as Lee et al. 2012, with a focus on depression

Kim et al 2013 (146) (146), same study as Lee et al. 2012, with a focus on suicides

Cha et al. 2014 (147)

- Period of study = 2008-2012
- Data from the national death registry gives 455 fatalities annually (averaged from 2006-2010)

Lee et al. (2014) (148)

- Period of study = 2006-2009
- Analysed data of the National Health Insurance which covers the entire South Korean population for children <14 yrs of age. All patient medical utilization related to pesticide poisoning is expected to be included in the database
- Morbidity = 0.0036% (307 cases)

- 1 fatality per year

Moon et al. 2016 (149)

- Period of study = 2011-2014
- Analysed data of the "Injury in-depth Surveillance system", which is operated by the Korea Center for Disease Control and Prevention and includes data of 20 emergency departments of hospitals in South Korea
- Annual average of 150 non-fatal cases
- No information is given on the nation-wide coverage of included departments but authors state that their results are compatible to other South Korean studies.
- Data not ok to extrapolate

Cha et al. 2016 (151)

- Provides data for accidental poisoning, showing 56 annual fatalities on the average 2008-2012
- Same data source as Cha et al 2014

Kim et al. 2017 (150)

- Period of study = 2008-2012
- Same data source as Cha et al 2014; gives 51 fatalities annually (average 2009-2013). The difference to Cha et al. is due to case identification. Both papers seem to make use of the ICD x-codes allowing differentiation of the external causes of poisoning. However, Kim et al. 2017 report separately for "accidental" and "undetermined" whereas Cha et al. 2014 include "undetermined" along with others in "unintentional"
- Data ok to extrapolate

Publications 2019-2023 extracted for synthesis: 2

Moon et al. 2021 (152)

- Period of study = 2011-2016
- Update of Moon et al 2016 (149)
- Annual 62 UAPP in children 7-18 years of age
- One child died "after accidental exposure to domestic repellents"

Shin and Roh 2019 (153)

- Period of study = period 2014, 2015, 2017
- Sample = 323, orchard farmers in south Chungcheong Province
- Acute occupational pesticide poisoning was defined as experiencing at least 1 out of 21 clinical symptoms provided in the questionnaire within 48 hours after exposure to pesticides over the last 1 year
- Morbidity = 16% (52 cases)
- Data ok to extrapolate

WHO mortality data: data were available from the WHO Mortality database, reporting 12.2 fatal UAPP annually in the period 2017-2021 for the general population and 1.2 cases for farming population. Zero cases were reported for children in this period.

Estimation of national figures

With respect to mortality, we use the WHO figures as the most recent period. For non-fatal poisoning, we based our calculations for the general population on Kim et al. 2012 with a rate of 2.29 per 100,000.

For farmers, non-fatal UAPP is based on Lee et al. 2012 (as the country-wide representative study) and Shin & Roh 2019, which also included female farmers. We averaged the annual prevalence of both studies (23%). Non-fatal UAPP for children was based on Lee et al. 2014. We excluded Moon et al. 2016 and Moon et al. 2021, because the catchment area was not given. Other studies were excluded because they reported on the same data extracted in other studies: Kim et al. 2013, Kim et al. 2017.

Annual national estimates for South Korea

**Population type**	Year	**Population**	**Fatal**	**Non-fatal**	**Total cases**
General	2009-2013	49,885,466		1,142	
	2017-2021	51,658,581	12.2		1,156
Farming/occupational	2011,2014,2015,2017	1,500,527		362,507	
	2017-2020	1,475,173	1.2		362,509
Children	2006-2009	8,556,926		307	
	2017-2020	6,438,312	0		307

National estimates based on studies with following study characteristics

**Criteria**	**Farming/occupational population**
Number of studies used for calculation	2
Review periods covered (first, second, both)	both
Sampling (representative, simple sample or unspecified)	representative sample
Diagnose (study scientists, self-reported)	self-reported
Latency period (24 h, 48 h, other, unspecified)	48 h
At-risk time (annual, <1 y, other, ever, unspecified)	annual
No. of symptoms (>=1, >=2, unspecified)	>=1

51. Spain

Publications extracted for synthesis 2006-2018: 1

Publications extracted for synthesis 2019-2023: 1

Tomenson and Matthews 2009 (4):

- Period of study = 2006
- Survey of pesticide applicators and farmers commissioned by Syngenta Crop Protection among 6,359 users in 24 countries
- Sample = 250, representative, farmers and pesticide users
- Morbidity = 30% (75 cases)
- Timeframe = past 12 months

WHO Mortality data were available from the period 2017-2021 giving 2.8 annualized fatal cases for the general population and 0.2 for children and the farming/occupational population respectively.

Estimation of national figures

Tomenson and Matthews reported 30% pesticide poisonings among pesticide users and the survey was conducted in 2006.

0.30*783,154 =234,946.2

National estimates for Spain

Population type	Year	Population	Fatal	Non-fatal	Total cases
General	2017-2021	47,061,455	2.8		2.8
Children	2017-2021	6,872,042	0.2		0.2
Farming/occupational	2006	783,154		234,946	
	2017-2021	950,593	0.2		234,946.2

National estimates based on studies with following study characteristics

Criteria	Farming/occupational population
Number of studies used for calculation	1
Review periods covered (first, second, both)	first
Sampling (representative, simple sample or unspecified)	representative
Diagnose (study scientists, self-reported)	self-reported
Latency period (24 h, 48 h, other, unspecified)	unspecified
At-risk time (annual, <1 y, other, ever, unspecified)	annual
No. of symptoms (>=1, >=2, unspecified)	unspecified

52. Taiwan

Publications extracted for synthesis 2008-2018: 2

Chien et al. 2011 (154)

- Period of study = 1999-2008
- National data, applying to all pesticides
- Accidental and occupational
- 47.5% of hospitalisations were farm workers; 16.5% of deaths were occupational
- Average 59.4 deaths per year; incidence rate = 0.0002579%
- Average 633 not-fatal hospitalizations/year; incidence rate = 0.00274772%
- Source = Health Insurance Database from 1999 to 2008 released by the Taiwan National Health Research Institute (NHRI) in 2009
- Mortality rate fell from 0.8 per100,000 in 2000, to 0.5 in 2008, so the data does not give a good representation of the situation for 2006-2008
- Hospitalization rate fell by 60% over the period
- Decisions was to review whether to use this in conjunction with other Taiwan studies
- Decision then taken that these data were too old

Chien et al. 2013 (155)

- Period of study = 2005-2007
- National figures, based on hospitalizations
- Average 470 non-fatal hospitalizations per year; incidence rate = 0.00204348%
- Data ok to extrapolate

Publications extracted for synthesis 2019-2023: 1

Wu et al. 2023 (156)

- Period of study = 2001-2016
- Study on long-term trends of pesticide poisoning in agricultural workers
- Data from National Health Insurance Research Database 2001-2016
- In 2016 the age-standardized incidence of pesticide poisoning was 18.98 per 100.00
- No further details as paper is abstract only
- Data from Wu et al. may include fatalities and suicides

WHO Mortality data: no mortality data were available.

Estimation of national figures

The data from Wu et al 2013 may include fatalities and suicides so this study was not used for the national estimate of UAPP.

Annual National estimates for Taiwan

Population type	Year	Population size	Fatal	Non-fatal	Total
General	2005-2007	22,927,215		470	470

Population 2007 from worldometers, farming population from WIKIPEDIA

Limitations

The data for the general population is from hospitalisations and therefore is likely to be an underestimate.

53. Tanzania

Publications extracted for synthesis 2006-2018: 7

Publications extracted for synthesis 2019-2023: 7

Tomenson and Matthews 2009 (4)

- Period of study = 2006
- Sample = 250, representative, farmers and workers
- Morbidity = 74.8% (187 cases), any symptom over past 12 months
- Data ok to extrapolate

Lekei et al. 2014 (157)

- Period of study = date not given
- Area of study = Arumeru district in Arusha region, the villages of Uwiro, Olkung'- wado, Nguruma, Moivaro, Makisoro, Ambureni and Sing'isi comprising about 5% of all villages in Arumeru district. The selected villages were typical of vegetable and coffee growers of Arumeru district
- Sample = 121, representative, vegetable farmers
- Morbidity = 93% (112 cases), any symptom, ever
- Study not considered for extrapolation because of 'ever' prevalence

Lekei et al. 2014 (158)

- Period of study = 2006
- Part of the study reviewed hospital admission data for APP retrospectively (2000–2005) in 30 facilities in four regions of Tanzania (covering 30% of all hospital beds in Tanzania). A prospective follow-up over 12 months in 2006 focused on 10 facilities with the highest reporting of APP; data is extracted only for the 2006 follow-up
- The prospective study found an incidence rate of 4.04 per 100,000; mortality rate of 0.2 per 100,000
- 31% of known poisoning cases were accidental, 10.2% of known poisoning cases were occupational, 1.1% of cases were homicidal
- Morbidity = 0.000011579%
- Mortality = 0.000000175%
- Occupational morbidity = 0.0000041208%; occupational mortality was not known
- Homicidal mortality = 0.00000002%
- Data ok to extrapolate

da Silva et al. 2016 (159)

- Period of study = 2013
- Area of study = 7 villages on Unguja Island, main island of Zanzibar Archipelago
- Sample = 99, representative, rice farmers, 95% male
- Morbidity = 69% (68 cases), any symptom
- Data ok to extrapolate

Lekei et al. 2016 (160)

- Period of study = 2006
- This paper is a re-analysis of data from Lekei et al. 2014a and 2014b, which are more detailed and have been extracted
- Estimated high, median and low correction factors for underestimation were 71.4, 22.2 and 9.6, respectively, to include occupational UAPP cases occurring in the community that are not detected from routine facility-based surveillance
- The underlying rate of occupational UAPP based on the lower boundary of the correction factor for under-estimation would be 11.3 to 37.6 cases per 1,000,000 and based on the upper boundary of the correction factor, rises to 84.3 to 280.0 cases per 1,000, 000. So, the range = 11.3 to 280 per 1,000,000
- Depending on the choice of scenario and under-reporting correction factor used, occupational UAPP could comprise from 52.2 to 96% of all UAPP cases

Lekei et al. 2017 (161)

- Period of study = 2006
- 10 health facilities in 4 districts; same data as used in the prospective part of Lekei et al. 2014b but focussing on children
- Children only
- Sample = 3,285,298
- 31 cases: accidental = 26, occupational = 4, homicide = 1
- Morbidity = 0.00094360%
- Paper states a "population-based incidence rate" in northern Tanzania
- Data ok to extrapolate

Manyilizu et al. 2017 (162)

- Period of study = 2015
- Area of study = Arusha: two rural areas in northern Tanzania, namely Lake Eyasi and Ngarenanyuki in adjacent Karatu and Meru districts, respectively. The main crops produced for sale were tomatoes and onions in the Meru and Karatu districts
- sample = 128, representative, farmers, 86% male
- Morbidity = 76.6% (98 cases), for most prevalent symptom, within the last 3 months
- Data ok to extrapolate

WHO Mortality data: no mortality data were available.

Estimation of national figures

The study by Lekei et al. 2014 (157) was excluded because of the 'ever' prevalence. The remaining three studies (Tomenson and Mathews 2009, da Silva 2016, Manyilizu 2017) were averaged. Lekei et al. 2014b and Lekei et al. 2017 were included for synthesis for the child population.

Study	Study year	Sample	Morbidity (%)
da Silva 2016	2013	99	61
Manyilizu 2017	2015	251	76.6
Tomenson and Mathews 2009	2006	250	74.8
Average			*70.8*

Annual National estimates for Tanzania

Population type	Year	Population	Fatal	Non-fatal	Total cases
General	2006	40,634,948	7.1	470	477.1
Farming/occupational	2013-2015	15,940,378		11,285,788	11,285,788
Children	2006	18,423,465		173	173

National estimates based on studies with following study characteristics

Criteria	Farming/occupational population
Number of studies used for calculation	3
Review periods covered (first, second, both)	first
Sampling (representative, simple sample or unspecified)	representative sample
Diagnose (study scientists, self-reported)	self-reported
Latency period (24 h, 48 h, other, unspecified)	48 h
At-risk time (annual, <1 y, other, ever, unspecified)	annual
No. of symptoms (>=1, >=2, unspecified)	>=1

54. Thailand

Publications extracted for synthesis 2006-2018: 5

Kachaiyaphum et al. 2010 (163)

- Period of study = 2007, Oct-Dec
- Area of study = Chatturat District, Chaiyaphum Province, 342 km northeast of Bangkok, where chilli is a major product; 5 of the 9 sub-districts were selected for study, 70 of the 75 villages (5 villages had no chilli growing), 5 households from each village
- Estimated population of Chatturat District = 75,948 people living in 22,401 households
- Sample = 350, representative, 62% male, chilli farmers and workers
- Morbidity = 38% (133 cases) most prevalent symptom
- Data ok to extrapolate

Sapbamrer and Nata 2014 (164)

- Period of study = August and Oct 2012
- Area of study = Bam Tom subdistrict, which is located close to Kwan Phayao Lake, northern Thailand
- Sample = representative, 50.5% females, 182 rice farmers
- Morbidity = 42.9% (78 cases), most prevalent symptom within 1 month prior to interview
- Data ok to extrapolate

Sapbamrer et al. 2017 (165)

- Period of study = 2014, Sept-Oct
- Aera of study = Phayao Province, Northern Thailand
- Sample = 84 farmers
- Morbidity = 29% (24 cases), most prevalent symptom
- Data ok to extrapolate

Thetkathuek and Jaidee 2017 (166)

- Period of study = date not given but before 2017 and presumed to be after 2006
- Area of study = area of concentrated tropical fruit production in three adjacent coastal provinces (Chonburi, Rayong, and Chanthaburi) in the eastern region of Thailand
- Sample = 891, migrant agricultural workers from 78 fruit farms who self-reported pesticide exposure and whose employers reported organophosphate and/or carbamate use on farm, 57.5% male
- Morbidity = 87.88% (783 cases), 74.3% with more than one symptom
- Data not extrapolated — study was not representative because of a very selective sample

Kongtip et al. 2018 (167)

- Period of study = August 2013 to February 2014
- Area of study = provinces of Nakorn Ratchasima in the northeast, Phisanulok province in the lower north, and Payao province in upper north of Thailand. 11.4 million people (38% of population) work in agriculture in Thailand

- Sample = 424, representative, vegetable farmers (mostly) and workers (60% female), representing 5 farm types: rice, vegetable, flower, rice/vegetable, and flower/vegetable
- Morbidity = 34.2% (145 cases), most prevalent symptom – last 3 months
- Data ok to extrapolate

Publications extracted for synthesis 2019-2023: 1

Ong-Artborirak et al 2022 (168)

- Period of study: 2020-2021
- Area of study = 6 districts out of 25 in Chiang Mai Province
- Sample = 6,045 farmers, 53.8% male
- Morbidity = 17.3% (1,045 cases)
- "lifetime exposure history to pesticides" was reported, so the study was excluded

WHO Mortality data: available for 2017-2019: 49 fatalities, including 5 children and 2 for the farming population, = 16.3 per year (general), 1.7 (children), 0.7 (farming).

Estimation of national figures

Study	Year	Sample	Morbidity (%)
Kachaiyaphum 2010	2007	350	38
Kongtip 2018	2013-14	424	34.2
Sapbamrer & Nata 2014	2012	542	42.9
Sapbamrer et al. 2017	2014	84	29
Average			*36*

We did not consider the study by Ong-Artborirak et al 2022, as very likely an ‘ever’ prevalence was reported. The four studies from the first review period on farmers and workers were averaged to arrive at a percentage occupational morbidity of 36.03%. The additional study of immigrant fruit farm workers who were exposed to organophosphates and/or carbamates, although not included here, should be noted because it indicates very high APP in at least one sector of the Thai agricultural economy.

Annual national estimates for Thailand

Population	Year	Population	Fatal	Non-fatal	Total cases
General	2017-2019	71,111,256	16.3		16.3
Children	2017- 2019	11,790,288	1.7		1.7
Farming/occupational	2012-2016	14,170,704	0.7	5,105,704	5,105,704.7

National estimates based on studies with following study characteristics

**Criteria**	**Farming/occupational population**
Number of studies used for calculation	4
Review periods covered (first, second, both)	first
Sampling (representative, simple sample or unspecified)	representative
Diagnose (study scientists, self-reported)	self-reported
Latency period (24 h, 48 h, other, unspecified)	other
At-risk time (annual, <1 y, other, ever, unspecified)	<1 y
No. of symptoms (>=1, >=2, unspecified)	unspecified

55. Turkey

Publications extracted for synthesis 2006-2018: 0

Publications extracted for synthesis 2019-2023: 1

Cevik et al. 2020 (169)

- Period of study = 2019
- Sample = 565, male farmers of the Karacabey district
- Morbidity = 11.3% (64 cases) reported UAPP – annual poisonings with at least two symptoms that occurred within 48 hours

WHO Mortality data: Available for 2017-2019, 11.3 fatal pesticide poisonings per year for the general population, 1.7 cases for children and no cases for the farming population were reported.

Estimation of national figures

For the estimation we used the male farming population only: 11.33 % *3,345,525 = 379.048
Data for children and general population based on WHO data.

Annual national estimates for Turkey

Population type	Year	Population size	Fatal	Non-fatal	Total cases
General	2017-2019	81,433,114	11.3		11.3
Children	2017-2019	19,561,279	1.7		1.7
Farming/occupational	2017-2019, 2019	3,345,525*	0	379,048	379,048

* Male farming/occupational population

National estimates based on studies with following study characteristics

Criteria	Farming/occupational population
Number of studies used for calculation	1
Review periods covered (first, second, both)	second
Sampling (representative, simple sample or unspecified)	representative
Diagnose (study scientists, self-reported)	self-reported
Latency period (24 h, 48 h, other, unspecified)	48 h
At-risk time (annual, <1 y, other, ever, unspecified)	annual
No. of symptoms (>=1, >=2, unspecified)	>=2

56. Uganda

Publications extracted for synthesis 2006-2018: 4

Okonya and Kroschel 2015 (170)

- Period of study = not reported but likely to be 2013
- Area of study = 6 sub-counties
- Sample= 204, survey of potato farmers, about half male/female
- Morbidity = 75% (154 cases) reported having felt sick after pesticide application

Clausen et al. 2017 (171)

- Period of study = 2012
- Sample = 114, 57% female, survey of small-scale farmers with cotton as main product, after an IPM intervention in Wakiso and Pallisa – the same districts as mentioned above
- Symptoms self-reported
- 13 acute symptoms caused by pesticide exposure were read out loud, and farmers affirmed whether they had experienced the symptom working with pesticides within the last 6 months
- Morbidity = 57% (65 cases), experienced at least 3 symptoms

Pedersen et al. 2017 (172)

- Period of study = 2013-2016
- Reports on an urban and a rural study. The urban study is the same as published by Ssemugabo et al. The rural study was carried out in the districts of Wakiso and Pallisa and included data from 22 of 61 Health Centers. Wakiso has a population of 2 million, Pallisa of 387,000. The data were collected by clinical staff who had received training in pesticide diagnosis and treatment of poisonings
- Annually 16 cases
- The mortality ratio was given as 3.8 % but intentional/unintentional not differentiated

Ssemugabo et al. 2017 (173)

- Period of study = 2010-2014
- Conducted in 5 hospitals in Kampala. The hospitals were selected for being the largest within the city and because treatment of a large number of pesticide poisoning patients. The hospitals are a mix of a national referral hospital, not-for profit, or private hospitals
- According to the authors Kampala has a population of about 1,516,210 of the country’s 34.8 million people
- Morbidity = annual average of 16 non-fatal cases

WHO Mortality data: no mortality data were available.

Estimation of national figures

The hospital-based studies Pedersen et al. 2017 and Ssemugabo et al. 2017 were excluded because the papers do not give information on the total number of hospitals in Kampala, or the rural districts, or the coverage by the selected hospitals, or the catchment areas.

In order to estimate the nation-wide number of non-fatal pesticide poisoning we averaged the results from Okonya and Kroschel 2015 and Clausen et al. 2017.

Annual national estimate for Uganda

Population type	Year	Population	Fatal	Non-fatal	Total cases
Farming/occupational	2012-2013	9,021,117		5,953,937	5,953,937

National estimates based on studies with following study characteristics

Criteria	Farming/occupational population
Number of studies used for calculation	2
Review periods covered (first, second, both)	first
Sampling (representative, simple sample or unspecified)	representative
Diagnose (study scientists, self-reported)	self-reported
Latency period (24 h, 48 h, other, unspecified)	other
At-risk time (annual, <1 y, other, ever, unspecified)	<1 y
No. of symptoms (>=1, >=2, unspecified)	>=2

57. United Kingdom

Publications extracted for synthesis 2006-2018: 2

Publications extracted for synthesis 2006-2018: 0

Solomon et al. 2007 (174)

- Period of study = unknown
- Area of study = three rural areas in England and Wales: north Devon, the Welsh borders and south Lincolnshire
- Sample = 4,108, male farmers and workers
- Non-fatal cases reported as those who had ever used pesticides who experienced symptoms within 48 hours of use
- Morbidity = 23% (944.84 cases), reported as having experienced symptoms
- This study reported a lifetime prevalence, but no other data were available for the farming/occupational population, therefore these data were used for the estimate

Perry et al. 2014 (175)

- Period of study = 2004-2013
- Data from the UK National Poisons Information Service included mortality and morbidity cases
- Data taken for extraction were based on 2010-2013 for acute unintentional poisonings: 6,789 unintentional acute pesticide exposures over 2004-2013; 3,727 total exposures for 3 years from 2010-2013, "a minimum estimated incidence of pesticide exposure requiring health care contact"
- 87% of the cases were acute unintentional poisonings = 3727 x 87% = 3242 = 1,081/yr
- Fatal cases were reported from 2004-2013 at a rate of 1 per year of unintentional poisonings for adults in the general population

WHO Mortality data: available for the period 2017-2020.

Estimation of national figures

WHO mortality data were more current than data from Perry et al. 2014. The mortality data reported by the WHO for the UK general population were 3.75 cases per year from 2017-2020 and zero for the children and farming population.

For acute unintentional exposures of children, Perry et al. reported that 54.1% of the cases were involving children ≤ 12 years of age. “More acute unintentional exposures involved children (≤12 years) than adults: 3,674 (54.1%) exposures were in children, 3,058 (45.0%) exposures were in adults (57 unknown [0.8%]).” So, we calculated the children unintentional poisonings based on the general populations estimate, taking 54.1% of the general population estimate, and we can take this as a lower boundary estimate for accidental pesticide poisonings of children: 0.541* 1,081 non-fatal pesticide poisonings in general population = 584.8 children cases <=12 years**.**

Solomon et al. 2007 data were used for national estimates based on the 2006 farmer and worker population.

National estimates for UK

Population type	Year	Population	Fatal	Non-fatal	Total cases
General	2010-2013	64,165,893		1,081	
	2017-2020	66,609,191	3.75		1,084.75
Children	2017-2020	11,268,488	0	584.8	584.8
Farming/occupational	2006	397,175		91,350	
	2017-2020	371,418	0		91,350

National estimates based on studies with following study characteristics

Criteria	Farming/occupational population
Number of studies used for calculation	1
Review periods covered (first, second, both)	first
Sampling (representative, simple sample or unspecified)	simple sample
Diagnose (study scientists, self-reported)	self-reported
Latency period (24 h, 48 h, other, unspecified)	48 h
At-risk time (annual, <1 y, other, ever, unspecified)	ever
No. of symptoms (>=1, >=2, unspecified)	>=1

58. United States of America

Publications extracted for synthesis 2006-2018: 25

Publications extracted for synthesis 2019-2023: 9

There is no national mechanism collecting data on all the pesticide poisonings in the US. For the reports by the American Association of Poison Control Centers (AAPCC), the most recent 5 years of data were used. The National Pesticide Information Center collects data that is shared with the US EPA, as do the AAPCC. Both publish annual reports.

The Sentinel Event Notification System for Occupational Risk-- Pesticides (SENSOR) has 13 participating states. SENSOR states have agreed to submit pesticide poisoning data to the US Centers for Disease Control, which publishes studies on some of the SENSOR data. California is a SENSOR state, and the California Department of Pesticide Regulation publishes an online database on poisonings, including drift and poisoning incidents. The US EPA Incident Data System has a searchable database of pesticide incidents over the past 10 years, but these two databases do not issue reports and therefore were not included in this study.

National studies

Yin 2011 (176)

- Period of study = 2000-2008
- National level data for children less than 7 years of age
- 0 fatalities reported
- 45 non-fatal cases of pesticide poisoning reported for malicious pesticide poisonings only
- These data were not reported as annual cases, but the time period from 2006-2008 can be taken with the assumption that the same number of cases occurred per year. The US population of children from 2006-2008 would be taken

Langley and Mort 2012 (177)

- Period of study = 2006-2010.
- Entire US population; various data sources including poison control centres and CDC.
- These data included mortality data, and the population for the time period was 292,500,000 with 22 fatalities per year and 41,132 non-fatal cases per year

Kamboj et al. 2018 (178)

- Period of study = 2000-2016
- Reported data on ocular exposure for the entire US population (poison control centres)
- The number of moderate and major cases for 2012-2016 were 5,540 or 1,108 cases per year * 4.23%= 46.8684 moderate/major cases per year for that period. It was not possible to track or calculate "minor" cases reported in this study – there was a figure of 51.1% "minor cases" for all compounds given, but it was questionable whether this can be used for pesticides.
- Pesticides moderate and major outcomes = 4.23% of ocular exposure for the entire period of 2000-2016. We made the assumption that the same percentage applied to the period of time we used for data analysis

National Pesticide Information Center (NPIC) reports

NPIC receives calls and online reports, and the data are sent to the US EPA. They have limited hours and are not as well-known as the US poison control centers. NPIC produces annual reports specifically on pesticide poisonings. Based on our previous assessment (Boedeker et al. 2020), the NPIC data are not as comprehensive, and the reports not used in this study.

American Association of Poison Control Centers (AAPCC) reports – 2018-2022

For AAPCC reports, fatalities data were not extracted because the unintentional poisonings cases were not reported separately. Child unintentional pesticide poisonings were reported as the cases in children 12 years of age or younger. AAPCC reports additionally on the medical outcomes of cases including a "none" category. Since this follow-up is restricted to less than 50 % of all cases, the distribution remains unknown for pesticide poisoning, and we extracted the unadjusted number of cases. For children, no stratification on intentional and unintentional cases were provided. However, to arrive at UAPP, we subtracted from cases by the share of overall intentional poisoning.

Gummin et al. 2019 (179)

- Reported data from 2018
- The general population (330,000,000) had 72,192 non-fatal unintentional cases per year
- The prevalence for non-fatal poisonings was 0.02% in the general population and for children the prevalence was 0.06%

Gummin et al. 2020 (180)

- Reported data from 2019
- The general population (334,000,000) had 72,246 non-fatal unintentional poisoning that year
- The prevalence for non-fatal poisonings was 0.02%
- The child population had a prevalence of 0.06%

Gummin et al. 2021 (181)

- Reported 2020 data
- The non-fatal unintentional poisoning cases = 66,362 cases in 2020 for the general US population (336,400,000) with a prevalence of 0.02%
- The child population had a prevalence of 0.05%

Gummin et al. 2022 (182)

- Reported 2021 data
- The non-fatal unintentional poisoning cases = 62,225 cases in 2021 for the general (338,700,000) US population that year with a prevalence of 0.02%
- The child population had a prevalence of 0.05%

Gummin et al. 2023 (183)

- Reported 2022 data
- The non-fatal unintentional poisoning cases = 62,275 cases for the general US population (337,000,000) with a prevalence of 0.02%
- The child population had a prevalence of 0.05%

WHO Mortality data: available from 2017-2021.

Publications (regional) extracted for synthesis 2019-2023

Lee et al. 2010 (184)

- Period of study 2006-2007
- Reported non-fatal exposures to fipronil and most of the data were pre-2006
- They reported 26 exposures per year for 2006-2007 period, in SENSOR states for a mixed population. Data from 12 SENSOR states were reported, excluding Arizona because so few cases of poisoning were reported: CA, FL, IA, LA,MI,NM,NY,NC,OR,TX, WA (AZ excluded, IA: 2006-2007; NM 2005-2007; NC 2007 only)

Lee et al. 2011 (185)

- Period of study = 2006
- reported drift cases, both occupational and nonoccupational non-fatal cases
- The population was 124,500,00 with 236 total cases for 2006 (prevalence = 0.0001896%), to be extrapolated to the whole US population
- Data from 11 SENSOR states were included in this study, including data from the California Department of Pesticide Regulation
- The study was not used in the national estimation because of the focus on drift cases only, and thus excluded the majority of UAPP

Kasner et al. 2012 (186)

- Period of study = 2000-2007
- These data were from SENSOR and CA DPR
- Farmworkers, 70% of the cases were male
- Non-fatal cases per year = 228, out of a population of 325,007.
- No fatalities reported
- Data ok to extrapolate

Spiller et al. 2013 (187)

- Period of study = 2006-2010
- Reported non-fatal poisonings of children under 6 years for 5 US states: KY, LA, NC, OH, TX
- The population was reported in the study as 54,900,000 and number of cases per year was 8,005. These data were from poison centres only in those states

Hudson et al. 2014 (188)

- Period of study = reported 2006-2008
- Data for 11 SENSOR states on non-fatal occupational pesticide poisonings with pyrethrin and pyrethroid pesticides
- 742 cases per year from 60,627,451 full time equivalents, calculated by the study scientists

Calvert et al. 2016 (189)

- Period of study = 2007-2011
- Occupational pesticide poisonings based on data from 12 states participating in the SENSOR program. Two of the states did not provide data for the entire period (Nebraska: 2011 only; New Mexico: 2007-2008 only)
- The entire population 2007-2011 for full time equivalents estimates was 296,764,512, with a 0.9 incidence rate
- Occupational poisonings prevalence was 521 poisonings per year and 0.4 deaths per year, 66.5% were males
- The study also reported separately the farmer/worker population for the states was 4,486,533 with 833 poisonings
- Data ok to extrapolate

Higgins et al. 2016 (190)

- Period of study = 2007-2012
- State of North Carolina; this state participates in SENSOR programme, and perhaps it is redundant. The population was occupational with two of the major industries for that population being agriculture and structural pesticide use; non-occupational exposures were also reported but these probably included suicides, so these data were not extracted
- 39 cases non-fatal poisonings, 73% of occupational cases involved males
- 0 fatalities
- Data ok to extrapolate

Namulanda et al. 2016 (191)

- Period of study = 2007-2011
- Reported acute nonoccupational pesticide poisonings from 7 states, 1159 of a population of 72,280,376 participating in the SENSOR program from 2007-2011
- Prevalence calculated as 0.00160348% for the 7 participating states (FL, LA, MI, NC, NY, OR, WA)

Trueblood et al. 2016 (192)

- Period of study = 2009-2013
- Poisonings of children $\leq$ 19 years of age in the state of Texas, and included only hospitalizations
- 0.4 deaths per year, and 12 non-fatal cases per year for that period with catchment area of 7,621,714

Liu et al. 2018 (193)

- Period of study = 2007-2015
- Reported poisonings from total release foggers
- The data from 2013-2015 were reported for a 10,000,000 population (10 SENSOR states) and taken as the prevalence, 0.00027900%, for the region (the data used were from 2011-2015)

Publications (regional) extracted for synthesis, 2019-2023

Capper et al. 2019 (194)

- Period of study: 2000 to 2015, with the data from 2006-2015 used for this study
- The population includes occupational and general population cases
- Morbidity = 0.02% (911 cases reported for the state of Minnesota, with a population of 5,323, per year), 57.5% of occupational cases were male
- Data ok to extrapolate

Kyeremateng-Amoah et al. 2020 (195)

- Period of study = 2010 to 2015
- Hospital registry data for the state of Illinois for the general population.
- Fatalities data were available but did not distinguish between intentional self-harm, so these data were not used
- For non-fatal cases, 551 total cases were reported with the average population for the state at 12,873,333 = 6 cases per year

VanWormer et al. 2022 (196)

- Period of study = 2017-2021
- Hospital registry poisonings data for children in the state of Wisconsin
- 0 farm related non-fatal incidents in a sample population of 4,730

Parks et al. 2023 (197)

- Period of study = 199-2016
- Reported occupational poisonings from North Carolina using 'ever' prevalence
- Because there are other studies available for occupational poisonings, these data were not included in the estimate

Estimation of national figures

There were many studies contributing to the data synthesis. For national estimates of non-fatal UAPP, preference was given to studies or reports that had national level data. The national estimates for the general and child populations did not involve averaging the prevalence because a decision was taken to rely on the American Association of Poison Control Centers dataset (AAPCC). This is the largest and most comprehensive dataset on non-fatal poisoning cases and is the best option for national reports on pesticide poisonings in the US.

WHO Mortality data were used as the most recent source.

Table: Contributing studies for general and child population

Population type	Study	Year	Population	Fatal	Non-fatal	Total cases
General	AAPCC	2018	330,000,000	Not used	72,192	
		2019	334,000,000		72,246	
		2020	336,400,000		66,362	
		2021	338,700,000		62,225	
		2022	337,000,000		62,275	
	Combined AAPCC	2018-2022	335,820,000		67,060	67,060
	WHO Mortality	2017-2021	328,766,669	10.6	NA	10.6
Children	AAPCC	2018	61,808,443	Not used	36,63.66	
		2019	61,515,572		35,648.19	
		2020	61,367,255		32,513.54	
		2021	60,572,632		30,085.76	
		2022	59,856,573		30,487.32	
	Combined AAPCC	2018-2022	61,024,095		32,183.70	
	WHO Mortality	2017-2021	61,443,785	0.6	NA	10.6

Some studies allowed for extrapolation to national estimates of the occupational poisonings counted in the US. We estimated the number of poisonings by multiplying the prevalence by the respective population.

We averaged the occupational non-fatal prevalence of the 4 studies in the following table. We excluded Hudson et al. because only specific active ingredients were studied.

Table: Contributing studies for extrapolations for the farming/occupational population

Population type	Study	Year	Population size	Fatal	Non-fatal	Total cases	Morbidity (%)
Occupational, SENSOR states	Calvert et al. 2016	2007-2011	897,306	0.4	166	166.4	0.0185 %
Occupational, poison control, Minnesota	Capper et al. 2019	2006-2015	192,688	NA	71.1	71.1	0.0369%
Occupational, North Carolina	Higgins et al. 2016	2007-2012	74,285*	0	39	39	0.0525005%
Farmer/farmworkers, SENSOR states	Kasner et al. 2012	2006-2007	325,007	NA	228	228	0.0701523%
Average		*2006-2012*					*0.044 %*

*Source: US Department of Agriculture National Agricultural Statistical Service data – average of the NC farmer/farmworker population from 2007 & 2011

Annual national estimates for USA

Population	Study	Year	Population size	Fatal	Non-fatal	Total cases
General	AAPCC 2018-2022	2018-2022	329,520,151		67,060	
		2017-2021		10.6		67,071
Children up to age 14	AAPCC 2018-2022 (0-12 years)	2017-2022	61,179,250		32,183.7	
		2017-2021		0.6		32,184.3
Farming/occupational	Calvert 2011, Capper 2019, Higgins 2016, Kasner 2012	2006-2021	2,556,507		1,125	
	2017-2021			0		1,125

The general and children population for the final summary was taken from the AAPCC data because these reports have the largest and most comprehensive dataset for the USA. The other national level studies focused on certain types of cases, such as individual pesticides only, or ocular poisonings in one study. For the AAPCC data, the population of children at or below the age of 12 were used for this summary in order to make an easier comparison to the WHO child population data.

The estimates for non-fatal poisonings for the farmer/farmworker was the result of averaging the prevalence from studies of the relevant population and extrapolating to the entire US population. World Bank data were used for the farmer/occupational population.

For the farming/occupational estimate, it was possible to separate out the farmer/farmworker population using data from three studies that specifically contained farmer/farmworker cases. Data on these populations from individual US states were from the US Department of Agriculture's (USDA) National Agricultural Statistics Service.

National estimates based on studies with following study characteristics

Criteria	Farming/occupational population
Number of studies used for calculation	4
Review periods covered (first, second, both)	both
Sampling (representative, simple sample or unspecified)	register
Diagnose (study scientists, self-reported)	study scientists
Latency period (24 h, 48 h, other, unspecified)	other
At-risk time (annual, <1 y, other, ever, unspecified)	annual
No. of symptoms (>=1, >=2, unspecified)	>1

Limitations

In Calvert et al. 2016, the full-time equivalents (FTE) for workers = 2000 hours worked, which represented 250 days of 8-hour days. It is possible that the FTE estimate is an underestimation, however, it was used by study authors, and we relied on it for calculations for this study. There is evidence that agricultural workers often exceed 8 hour days during certain times of the growing season. The occupational data were used to extrapolate for the farmer/farmworker population reported by World Bank. One limitation is that the occupational poisonings data includes workers who are not necessarily working primarily in agriculture. In addition, the estimated number of farmworkers in the US varies depending on the source, reporting may not be totally reliable due to a number of the workforce being undocumented fears of being persecuted due to immigration status can affect participation, and thus reporting, to the US census. Current estimates of the farmworker population alone are 2,184,493 for the US in 2022 according to USDA's Census of Agriculture. The USDA number is likely not an exact count for a few reasons, for example, nonfarm employers' worker numbers are not reported-- but this figure is likely the closest estimate. Source: https://www.nass.usda.gov/AgCensus/

The national AAPCC data used for children was available for children up to age 19, however, we used the population of children 12 years and under as reported by the AAPCC. In addition, the World Bank data set we are using was for US children aged 0-14 years. Though data on children past the age of 14 can be found in the US census, we used the World Bank data here because we are using it for every other country in order to estimate the child population.

59. Venezuela

Publications extracted for synthesis 2006-2018: 1

Publications extracted for synthesis 2019-2023:0

Gomez and Cáceres 2010 (198)

- Period of study = 2008
- Area of study = Aragua state
- Sample = 50 workers fumigating against dengue fever pest using organophosphate pesticides
- Sample was a simple sample or not stated, taken from the 78 individuals working in the dengue control programme, 98% male
- 62% of the 50 workers were affected which was calculated as 31 cases
- No fatalities
- The workers were also tested for the biomarker acetylcholinesterase, of which 38% had altered acetylcholinesterase levels

WHO Mortality data: Data were available for Venezuela from 2009-2013: 17.6 per year fatalities for the general population, 4.8 per year for children and 0.6 cases per year for the farming population.

Estimation of national figures

We did not find any data on pesticide applicators working on dengue control. We took the entire agricultural worker population as the group from which poisoned workers are calculated.

62% of the worker population were affected according to Gomez et al. 2010, the 2008 agricultural worker population was 1,007,399.

0.62* 1,007,399 = 624587.483

Table: National estimates for Venezuela

Population type	Year	Population size	Fatal	Non-fatal	Total cases
General	2009-2013	29,457,915	17.6		17.6
Children	2009-2013	8,669,169	4.8		4.8
Farming/occupational	2008	1,007,399		624,587	
	2009-2013	1,040,615	0.6		624,587

National estimates based on studies with following study characteristics

Criteria	Farming/occupational population
Number of studies used for calculation	1
Review periods covered (first, second, both)	first
Sampling (representative, simple sample or unspecified)	simple sample
Diagnose (study scientists, self-reported)	self-reported
Latency period (24 h, 48 h, other, unspecified)	unspecified
At-risk time (annual, <1 y, other, ever, unspecified)	unspecified
No. of symptoms (>=1, >=2, unspecified)	unspecified

Limitations

The estimate for farming/ occupational poisonings surveyed dengue control pesticide applicators working only with organophosphate pesticides. The individuals surveyed in the Gomez et al. 2010 study were workers, but it was not clear whether these applicators also worked in agriculture. The estimate used the data on the agricultural farmer/worker population, which may not reflect the number of workers actually using pesticides in general. It is likely that organophosphate pesticide use incurs greater numbers of poisonings, however, this is not certain and at any rate we do not have better data on the pesticide use situation in Venezuela.

60. Vietnam

Publications extracted for synthesis 2006-2018: 4

Publications extracted for synthesis 2019-2023: 1

Thong and Phong 2011 (199)

- Period of study = date not given
- Area of study = 3 communes Vinh Hanh, Vinh Binh and Vinh An Chau in the Thanh district of An Giang, in the Mekong Delta which covers 30,739 hectares, of which 97.4% is devoted to rice cultivation
- Sample = 45, representative, pesticide applicators
- Morbidity = 60% (27 cases), most prevalent symptom
- Data ok to extrapolate

FAO 2013 (34)

- Period of study = 2008
- Area of study = Hanoi and Thai Binh
- Sample = 251 farmers
- Morbidity = 55.38% (139 cases), any symptom
- Data ok to extrapolate

Schreinemachers et al. 2017 (35)

- Period of study = 2015
- Area of study = not stated
- Sample = 300, representative, vegetable farmers growing yard-long beans and leaf mustard, 50% female
- Morbidity = 30%, most prevalent symptom
- Data ok to extrapolate

Rengam et al. 2018 (9)

- Period of study = 2015-2017
- PANAP, in partnership with CGFED, worked with the women farmers from Hai Hau, a rural district in Nam Dinh Province (rice and vegetables). Farmers from Ao Sen and Dong Cham Villages in Thai Nguyen province were also engaged, through SRD.
- Sample = 534 farmers, purposive, 58.61% women
- Morbidity = 84% (450 cases) any symptom
- Data ok to extrapolate

Publications extracted for synthesis 2019-2023: 1

Diyana et al 2022 (11)

- Period of study = 2021
- Area of study = Hai Hau and Thuan Chau districts
- Sample = 103, farmers & workers, 51% female
- Morbidity = 52% (54 cases)

- Data ok to extrapolate

WHO Mortality data: no mortality data were available.

Estimation of national figures

Study	Study year	Sample	Morbidity (%)
Thong and Phong 2011	?	45	60%
FAO 2013	2008	251	55.38%
Schreinemachers et al 2017	2015	300	30%
Rengam et al 2018	2015-17	534	84%
Diyana et al 2022	2021	103	52%
Average			*56.3%*

The five studies on farmers and workers were averaged to arrive at a percentage occupational morbidity of 56.3 %. We used an average population for the years covered by the studies. From this we calculated the national estimate for UAPP in the farming/occupational population by 0.563*22,476,524 =12,654,283.

Annual national estimates for Vietnam

Population	Year	Population size	Fatal	Non-fatal	Total cases
Farming/occupational	2008,2015-2017,2021	22,476,524		12,654,283	12,654,283

National estimates based on studies with following study characteristics

Criteria	Farming/occupational population
Number of studies used for calculation	5
Review periods covered (first, second, both)	both
Sampling (representative, simple sample or unspecified)	representative sample
Diagnose (study scientists, self-reported)	self-reported
Latency period (24 h, 48 h, other, unspecified)	unspecified
At-risk time (annual, <1 y, other, ever, unspecified)	unspecified
No. of symptoms (>=1, >=2, unspecified)	unspecified

61. Zambia

Publications extracted for synthesis 2006-2018: 1

Publications extracted for synthesis 2019-2023: 0

Z'gambo et al. (200)

- Period of study = 2012
- 2 hospitals in Lusaka: retrospective extraction of data on acute poisoning cases from records at Levy Mwanawasa General Hospital (LMGH) and the University Teaching Hospital
- Morbidity = 61 cases
- Lusaka district has a total population of 1.7 million
- Lusaka district has 3 tertiary hospitals, 1 secondary hospital, 9 primary hospitals, 170 urban health centres and 11 "health post"
- Data not ok to extrapolate due to unclear catchment area

WHO Mortality data: no mortality data were available.

Estimation of national figures

There was only one study of hospital admissions, drawing on an unclear catchment area, so not suitable for extrapolation. No data on mortality. No summary or national estimates can be given.

62. Zimbabwe

Publications extracted for synthesis 2006-2018:1

Publications extracted for synthesis 2019-2023:0

Magauzi et al. 2011 (201)

- Period of study = 2006
- Area of study = Kwekwe district
- Representative sample = 246 workers, 60.6% male
- Morbidity = 45.1% (111 cases), any symptom, ever
- Data ok to extrapolate

WHO Mortality data: no mortality data were available.

Annual National estimates for Zimbabwe

Population type	Year	Population size	Fatal	Non-fatal (estimated)	Total cases
Farming/occupational	2006	4,400,493		1,984,622	1,984,622

National estimates based on studies with following study characteristics

Criteria	Farming/occupational population
Number of studies used for calculation	1
Review periods covered (first, second, both)	first
Sampling (representative, simple sample or unspecified)	representative
Diagnose (study scientists, self-reported)	self-reported
Latency period (24 h, 48 h, other, unspecified)	unspecified
At-risk time (annual, <1 y, other, ever, unspecified)	ever
No. of symptoms (>=1, >=2, unspecified)	unspecified

Limitations

The estimations were based on a study which reported ‘ever’ prevalences only.

References

1. Sulaj Z, Drishti A, Çeko I, Gashi A, Vyshka G. Fatal aluminum phosphide poisonings in Tirana (Albania), 2009 - 2013. Daru J Fac Pharm Tehran Univ Med Sci. 25. Januar 2015;23:8.

2. Butinof M, Fernandez RA, Stimolo MI, Lantieri MJ, Blanco M, Machado AL, u. a. Pesticide exposure and health conditions of terrestrial pesticide applicators in Córdoba Province, Argentina. Cad Saude Publica. März 2015;31(3):633–46.

3. Ling SY, Taylor D, Robinson J. Workplace chemical and toxin exposures reported to a Poisons Information Centre: A diverse range causing variable morbidity. Eur J Emerg Med. 2018;25(2):134–9.

4. Tomenson JA, Matthews GA. Causes and types of health effects during the use of crop protection chemicals: data from a survey of over 6,300 smallholder applicators in 24 different countries. Int Arch Occup Environ Health. August 2009;82(8):935–49.

5. Dey NC. Use of pesticides in vegetable farms and its impact on health of farmers and environment. Environ Sci Technol. 2010;134–40.

6. Miah SJ, Hoque A, Paul A, Rahman A. Unsafe use of pesticide and its impact on health of farmers: A case study in Burichong Upazila, Bangladesh. IOSR J Environ Sci Toxicol Food Technol. 2014;8(1):57–67.

7. Akhter N, Chakraborty TK, Ghosh P, Jahan S, Gosh GC, Hossain SA. Assessment of the using patterns of pesticides and its impact on farmers' health in the Jhenidah District of Bangladesh. Am J Environ Prot. 2016;5(5):139.

8. Akter M, Fan L, Rahman MM, Geissen V, Ritsema CJ. Vegetable farmers' behaviour and knowledge related to pesticide use and related health problems: A case study from Bangladesh. J Clean Prod. November 2018;200:122–33.

9. Rengam S, Serrana MS, Quijano I. Of Rights and Poisons: Accountability of the Agrochemical Industry [Internet]. Penang: PAN Asia Pacific; 2018 [zitiert 24. April 2020]. Verfügbar unter: https://panap.net/2018/10/of-rights-and-poisons-accountability-of-the-agrochemical-industry/

10. Kobashi Y, Haque SE, Nishikawa Y, Morita T, Nagami H, Sakisaka K, u. a. The Increase in Frequency of Protective Behavior against Pesticide Poisoning in Narail, Bangladesh through Use of an Easy Paper Checklist; an Interventional Study. Int J Environ Res Public Health. 4. September 2021;18(17).

11. Diyana, A., Rajendran D., Watts, M., Rengam, S., Alviar, S. Field Survey: Use and Impacts ofpesticides in Four Countries in Asia [Internet]. PAN Asia Pacific; 2022. Verfügbar unter: https://panap.net/resources/

12. Jørs E, Lander F, Huici O, Cervantes Morant R, Gulis G, Konradsen F. Do Bolivian small holder farmers improve and retain knowledge to reduce occupational pesticide poisonings after training on Integrated Pest Management? Environ Health Glob Access Sci Source. 1. Oktober 2014;13:75.

13. Faria NMX, Rosa JAR da, Facchini LA. [Poisoning by pesticides among family fruit farmers, Bento Gonçalves, Southern Brazil]. Rev Saude Publica. April 2009;43(2):335–44.

14. Santana V, Moura M, Nogueira F e. Occupational pesticide poisoning mortality, 2000-2009, Brazil. Rev Saude Publica. 2013;47(3):598–606.

15. de Albuquerque PCC, Gurgel IGD, Gurgel A do M, Augusto LG da S, de Siqueira MT. Health information systems and pesticide poisoning at Pernambuco. Rev Bras Epidemiol Braz J Epidemiol. September 2015;18(3):666–78.

16. dos Santos Cargnin MC, Echer IC, da Silva DR. Tobacco farming: use of personal protective equipment and pesticide poisoning. Rev Pesqui Cuid E Fundam Online. Juni 2017;9(2):466–72.

17. De Souza Campos AM, Bucaretchi F, Fernandes LCR, Fernandes CB, Capitani EM, Beck ARM. Toxic exposures in children involving legally and illegally commercialized household sanitizers. Rev Paul Pediatr. 2017;35(1):11–7.

18. Lermen J, Bernieri T, Rodrigues IS, Suyenaga ES, Ardenghi PG. Pesticide exposure and health conditions among orange growers in Southern Brazil. J Environ Sci Health Part B. 3. April 2018;53(4):215–21.

19. Magalhães AFA, Caldas ED. Underreporting of fatal poisonings in Brazil - A descriptive study using data from four information systems. Forensic Sci Int. Juni 2018;287:136–41.

20. Dalbo J, Filgueiras LA, Mendes AN. Effects of pesticides on rural workers: haematological parameters and symptomalogical reports. Bd. 24, CIENCIA & SAUDE COLETIVA. AVENIDA BRASIL, 4036, SALA 700 MANGUINHOS-1, RIO DE JANEIRO, 21040-36, BRAZIL: ABRASCO-ASSOC BRASILEIRA POS-GRADUACAO & SAUDE COLETIVA; 2019. S. 2569–82.

21. Silva DO da, Ferreira MJM, Silva SA da, Santos MAD, Hoffmann-Santos HD, Silva AMC da. Exposure to pesticides and acute intoxication in a region of intense agricultural production in Mato Grosso, Brazil, 2013. Epidemiol E Serv Saude Rev Sist Unico Saude Bras. 2019;28(3):e2018456.

22. Bortolotto CC, Hirschmann R, Martins-Silva T, Facchini LA. Pesticide exposure: a population-based study in a rural area in southern Brazil. Rev Bras Epidemiol Braz J Epidemiol. 2020;23:e200027.

23. de Araújo Nascimento F, Alves AA, Nunes HF, Miziara F, Parise MR, de Melo E Silva D. Cultivated areas and rural workers’ behavior are responsible for the increase in agricultural intoxications in Brazil? Are these factors associated? Environ Sci Pollut Res Int. Oktober 2020;27(30):38064–71.

24. Silvério ACP, Martins I, Nogueira DA, Mello MAS, Loyola EAC de, Graciano MM de C. Assessment of Primary Health Care for rural workers exposed to pesticides. Rev Saude Publica. 2020;54:09.

25. Xavier DM, Cezar-Vaz MR, Bonow CA, Schimith MD. Work accidents with children and youth in a rural environment in southern Brazil. Rev Lat Am Enfermagem. 2020;28:e3243.

26. dos Santos JCP, Valli JB, Sesse NS, Mackenzie-Ross S, Zandonade E, Ayres LR, u. a. Sociodemographic characteristics and exposure patterns of pesticide-related cases reported to a poison service center in Brazil between 2012 and 2016. Arch Environ Occup Health. 2021;76(8):494–503.

27. Fassa AG, Faria NMX, Szortyka ALSC, Meucci RD, Fiori NS, Carvalho MP de. Child Labor in Family Tobacco Farms in Southern Brazil: Occupational Exposure and Related Health Problems. Int J Environ Res Public Health. 22. November 2021;18(22).

28. Carvalho KP, Corassa RB, Petarli GB, Cattafesta M, Zandonade E, Salaroli LB. Exogenous pesticide poisoning in the state of Espírito Santo, Brazil, 2007-2016: spatial distribution and temporal trend in the incidence rate and case fatality ratio of notified cases. Epidemiol E Serv Saude Rev Sist Unico Saude Bras. 2022;31(2):e2021424.

29. de Melo MTB, de Almeida Santana GB, Rocha MHA, de Souza Lima RK, da Silva TAB, de Souza CDF, u. a. Epidemiological profile and temporal trend of exogenous intoxications in children and adolescents. Rev Paul Pediatr [Internet]. 2022;40((de Melo M.T.B., monica.melo@famed.ufal.br; de Almeida Santana G.B.; Rocha M.H.A.; Souza Lima R.K.; da Silva T.A.B.; de Souza C.D.F.; Rodrigues A.K.B.F.) Universidade Federal de Alagoas, Department of Medicine, Arapiraca, AL, Brazil). Verfügbar unter: https://www.embase.com/search/results?subaction=viewrecord&id=L2018818646&from=export

30. Faria NMX, Meucci RD, Fiori NS, Carret MLV, Mello-da-Silva CA, Fassa AG. Acute Pesticide Poisoning in Tobacco Farming, According to Different Criteria. Int J Environ Res Public Health. 5. Februar 2023;20(4).

31. Toe A, Ilboudo S, Ouedraogo M, Guissou P. Biological alterations and self-reported symptoms among insecticides-exposed workers in Burkina Faso. Interdiscip Toxicol. 2012;5(1):42–6.

32. Toe A, Ouedraogo M, Ouedraogo R, Ilboudo S, Guissou P. Pilot study on agricultural pesticide poisoning in Burkina Faso. Interdiscip Toxicol. 2013;6(4):185–91.

33. Jensen HK, Konradsen F, Jørs E, Petersen JH, Dalsgaard A. Pesticide use and self-reported symptoms of acute pesticide poisoning among aquatic farmers in Phnom Penh, Cambodia. J Toxicol. 2011;2011:1–8.

34. Food and Agriculture Organization (FAO) of the United Nations. Empowering Farmers To Reduce Pesticide Use [Internet]. Bangkok; 2013. (FAO Regional IPM/Pesticide Risk Reduction Programme in Asia. FAO-RAP). Verfügbar unter: https://www.researchgate.net/publication/259080275_Empowering_Farmers_to_Reduce_Pesticide_Risks

35. Schreinemachers P, Chen H-P, Nguyen TT, Buntong B, Bouapao L, Gautam S, u. a. Too much to handle? Pesticide dependence of smallholder vegetable farmers in Southeast Asia. Sci Total Environ. 2017;593–594:470–7.

36. Tandi TE, Wook CJ, Shendeh TT, Eko EA, Afoh CO. Small-scale tomato cultivators' perception on pesticides usage and practices in Buea, Cameroon. Health (N Y). 2014;06(21):2945–58.

37. Assokeng T, Siéliéchi JM, Noumi GB. Evaluation of health and environmental risks of pesticide products used in market-gardening in the city of Ngaoundere (Cameroon). J Agric Chem Environ. 2017;06(04):186–98.

38. Pouokam GB, Album WL, Ndikontar AS, Sidatt MEH. A pilot study in Cameroon to understand safe uses of pesticides in agriculture, risk factors for farmers' exposure and management of accidental cases. Toxics. Dezember 2017;5(4).

39. Achancho AA, Nsobinenyui D. The effects of pesticides on the health of peasant cocoa farmers in Munyenge, south west Cameroon. Agric Res J. 2019;15(1):10.

40. Boyd DR. Northern Exposure: Acute pesticide poisonings in Canada [Internet]. Vancouver, B.C.: David Suzuki Foundation; 2007. Verfügbar unter: https://davidsuzuki.org/science-learning-centre-article/northern-exposure-acute-pesticide-poisonings-in-canada/

41. Ramirez-Santana M, Iglesias-Guerrero J, Castillo-Riquelme M, Scheepers P. Assessment of health care and economic costs due to episodes of acute pesticide intoxication in workers of rural areas of the Coquimbo region, Chile. Value Health Reg Issues. 2014;5:35–9.

42. Munoz-Quezada MT, Lucero B, Iglesias V, Levy K, Muñoz MP, Achú E, u. a. Exposure to organophosphate (OP) pesticides and health conditions in agricultural and non-agricultural workers from Maule, Chile. Int J Environ Health Res. 2. Januar 2017;27(1):82–93.

43. Zhang X, Zhao W, Jing R, Wheeler K, Smith GA, Stallones L, u. a. Work-related pesticide poisoning among farmers in two villages of Southern China: a cross-sectional survey. BMC Public Health. 3. Juni 2011;11:429.

44. Zhang D, Zhang J, Zuo Z, Liao L. A retrospective analysis of data from toxic substance-related cases in Northeast China (Heilongjiang) between 2000 and 2010. Forensic Sci Int. 10. September 2013;231(1–3):172–7.

45. Zhang M, Fang X, Zhou L, Su L, Zheng J, Jin M, u. a. Pesticide poisoning in Zhejiang, China: A retrospective analysis of adult cases registration by occupational disease surveillance and reporting systems from 2006 to 2010. BMJ Open. 2013;3(11):e003510.

46. Zhang X, Wu M, Yao H, Yang Y, Cui M, Tu Z, u. a. Pesticide poisoning and neurobehavioral function among farm workers in Jiangsu, People's Republic of China. Cortex. Januar 2016;74:396–404.

47. Wang BS, Chen L, Li XT, Xu M, Zhu BL, Zhang J. Acute pesticide poisoning in Jiangsu Province, China, from 2006 to 2015. Biomed Env Sci. 2017;6.

48. Wang B, Li X, Han L, Shen H, Zhang J, Sun D, u. a. Childhood pesticide poisoning trend analysis of 13 years in Jiangsu, China. J Public Health Emerg. Dezember 2018;2:32–32.

49. Wang N, Wang B, Wen J, Li X, Pan L, Zhang J, u. a. Types of Exposure Pesticide Poisoning in Jiangsu Province, China; The Epidemiologic Trend between 2006 and 2018. Bd. 16, INTERNATIONAL JOURNAL OF ENVIRONMENTAL RESEARCH AND PUBLIC HEALTH. ST ALBAN-ANLAGE 66, CH-4052 BASEL, SWITZERLAND: MDPI; 2019.

50. Uribe MV, Diaz SM, Monroy A, Barbosa E, Paez MI, Castro RA. Exposure to pesticides in tomato crop farmers in Merced, Colombia: Effects on health and the environment. In: Soundararajan RP, Herausgeber. Pesticides - Recent Trends in Pesticide Residue Assay [Internet]. InTech; 2012 [zitiert 28. Februar 2019]. Verfügbar unter: http://www.intechopen.com/books/pesticides-recent-trends-in-pesticide-residue-assay/exposure-to-pesticides-in-tomato-crop-farmers-in-merced-colombia-effects-on-health-and-the-environme

51. Chaparro-Narváez P, Castañeda-Orjuela C. [Mortality due to pesticide poisoning in Colombia, 1998-2011]. Biomed Rev Inst Nac Salud. August 2015;35 Spec:90–102.

52. Varona ME, Díaz SM, Briceño L, Sánchez-Infante CI, Torres CH, Palma RM, u. a. [Determining social factors related to pesticide poisoning among rice farmers in Colombia]. Rev Salud Publica Bogota Colomb. August 2016;18(4):617–29.

53. Farnham A, Fuhrimann S, Staudacher P, Quirós-lépiz M, Hyland C, Winkler MS, u. a. Long-term neurological and psychological distress symptoms among smallholder farmers in costa rica with a history of acute pesticide poisoning. Int J Environ Res Public Health [Internet]. 2021;18(17). Verfügbar unter: https://www.embase.com/search/results?subaction=viewrecord&id=L2013537414&from=export

54. Ajayi OC, Akinnifesi FK, Sileshi G. Human health and occupational exposure to pesticides among smallholder farmers in cotton zones of Cote d'Ivoire. SciRes. 2011;3(10):631–7.

55. Gonzalez-Andrade F, Lopez-Pulles R, Estevez E. Acute pesticide poisoning in Ecuador: A short epidemiological report. J Public Health. 2010;18(5):437–42.

56. Lein PJ, Bonner MR, Farahat FM, Olson JR, Rohlman DS, Fenske RA, u. a. Experimental strategy for translational studies of organophosphorus pesticide neurotoxicity based on real-world occupational exposures to chlorpyrifos. Neurotoxicology. August 2012;33(4):660–8.

57. Nigatu AW, Bråtveit M, Moen BE. Self-reported acute pesticide intoxications in Ethiopia. BMC Public Health. 2016;16(575).

58. Adinew GM, Woredekal AT, DeVos EL, Birru EM, Abdulwahib MB. Poisoning cases and their management in emergency centres of government hospitals in northwest Ethiopia. Afr J Emerg Med. 2017;7(2):74–8.

59. Negatu B, Vermeulen R, Mekonnen Y, Kromhout H. Neurobehavioural symptoms and acute pesticide poisoning: A cross-sectional study among male pesticide applicators selected from three commercial farming systems in Ethiopia. Occup Environ Med. 2018;75(4):283–9.

60. Loha KM, Klous G, Lamoree M, de Boer J. Pesticide use and practice of local farmers in the Central Rift Valley (CRV) of Ethiopia: implications for the environment and health hazards. INTERNATIONAL JOURNAL OF PEST MANAGEMENT. 2-4 PARK SQUARE, MILTON PARK, ABINGDON OR14 4RN, OXON, ENGLAND: TAYLOR & FRANCIS LTD; 2022.

61. Tessema RA, Nagy K, Ádám B. Pesticide Use, Perceived Health Risks and Management in Ethiopia and in Hungary: A Comparative Analysis. Int J Environ Res Public Health. 3. Oktober 2021;18(19).

62. Tessema RA, Nagy K, Ádám B. Occupational and environmental pesticide exposure and associated health risks among pesticide applicators and non-applicator residents in rural Ethiopia. Front Public Health. 2022;10:1017189.

63. Baldi I, Robert C, Piantoni F, Tual S, Bouvier G, Lebailly P, u. a. Agricultural exposure and asthma risk in the AGRICAN French cohort. Int J Hyg Environ Health. Mai 2014;217(4–5):435–42.

64. Idowu AA, Sowe A, Bah AK, Kuyateh M, Anthony A, Oyelakin O. Knowledge, attitudes and practices associated with pesticide use among horticultural farmers of Banjulinding and Lamin of the Gambia. Afr J Chem Educ. 2017;16.

65. PAN UK. Supporting evidence-based pesticide regulation and risk reduction in Georgia, with a focus on vulnerable groups [Internet]. Brighton: Pesticide Action Network UK; (Final narrative report. PAN-UK in partnership with Eco-Life and the Secretariat of the Rotterdam Convention. FAO Project reference LoA/GF/UK/2015/AGPMR-P0318). Verfügbar unter: Available from:

http://www.pic.int/Implementation/TechnicalAssistance/Workshops/WorkshopGeorgiaOct2016/tabid/5824/language/en-US/Default.aspx

66. Moebus S, Bödeker W. Mortality of intentional and unintentional pesticide poisonings in Germany from 1980 to 2010. J Public Health Policy. 2015;36(2):170–80.

67. Ae-Ngibise K, Kinney P, Asante K, Jack D, Boamah AB, Whyatt R, u. a. Pesticide exposures in a malarious and predominantly farming area in Central Ghana. Afr J Environ Sci Technol. 30. August 2015;9(8):655–61.

68. Shetty A.R.S., Girish Chandra Y.P., Shivaji A., Praveen S. Pattern of poisoning in autopsy cases at a tertiary care center in bengaluru - A five year retrospective study. Indian J Forensic Med Toxicol. 2018;12(1):285–90.

69. Patil DA, Katti RJ. Modern agriculture, pesticides and human health: A case of agricultural labourers in western Maharashtra. J Rural Dev. 2012;31(3):14.

70. Banerjee I, Tripathi S, Roy As, Sengupta P. Pesticide use pattern among farmers in a rural district of West Bengal, India. J Nat Sci Biol Med. 2014;5(2):313.

71. Choudhary A. Adverse health effects of organophosphate pesticides among occupationally exposed farm sprayers: A case study of Bhopal Madhya Pradesh, India. Asian J Biomed Pharm Sci. 15. August 2014;4(35):30–5.

72. Peshin SS, Srivastava A, Halder N, Gupta YK. Pesticide poisoning trend analysis of 13 years: a retrospective study based on telephone calls at the National Poisons Information Centre, All India Institute of Medical Sciences, New Delhi. J Forensic Leg Med. Februar 2014;22:57–61.

73. Kumar D. Conditons of Paraquat Use [Internet]. Kerala: PAN India; 2015. Verfügbar unter: http://www.pan-india.org/paraquat-in-india-too-big-a-risk-for-farmers-and-workers/

74. Kaur M. Practices and health related toxic symptoms of pesticide use among farm workers. Glob J Res Anal. 2016;5(9):290–1.

75. Kumari D, John S. Safety and occupational health hazards of agricultural workers handling pesticides: A case study. In: Siddiqui NA, Tauseef SM, Bansal K, Herausgeber. Advances in Health and Environment Safety [Internet]. Singapore: Springer Singapore; 2018 [zitiert 27. Februar 2019]. S. 75–82. Verfügbar unter: http://link.springer.com/10.1007/978-981-10-7122-5_9

76. National Crime Records Bureau Ministry of Home Affairs. Accidental Deaths & Suicides in India 2021. 2023.

77. PAN India, PAN AP, SRED. Toxic Blooms. 2020.

78. Reshma H, Jayalakshmi R. Prevalence of acute pesticide poisoning among pesticide applicators in cardamom plantations: A cross-sectional study from Idukki District, Kerala. Bd. 24, INDIAN JOURNAL OF OCCUPATIONAL AND ENVIRONMENTAL MEDICINE. WOLTERS KLUWER INDIA PVT LTD , A-202, 2ND FLR, QUBE, C T S NO 1498A-2 VILLAGE MAROL, ANDHERI EAST, MUMBAI, 400059, INDIA: WOLTERS KLUWER MEDKNOW PUBLICATIONS; 2020. S. 188–93.

79. Thakur R, Jumade P, Waghmare R, Joshi S, Joshi A. Perceptions, Practices and Health Hazards, of Agricultural Workers from Rural Central India with Regard to Pesticide Use - A Cross Sectional

Study. Bd. 9, JOURNAL OF EVOLUTION OF MEDICAL AND DENTAL SCIENCES-JEMDS. C/O AKSHANTALA ENTERPRISES, NO 65, 1ST FL, SAHUKAR CHENNIAH RD, MYSORE, KARNATAKA, 570 009, INDIA: JOURNAL EVOLUTION MEDICAL & DENTAL SCIENCES; 2020. S. 3528–32.

80. Kumari D, Sebastian AJ, John S. Pesticide handling practices and health risks among the apple orchard workers in Western Indian Himalayan region. Bd. 27, HUMAN AND ECOLOGICAL RISK ASSESSMENT. 530 WALNUT STREET, STE 850, PHILADELPHIA, PA 19106 USA: TAYLOR & FRANCIS INC; 2021. S. 15–29.

81. Lohan SK, Singh P, Kumar S. Agricultural work-related fatalities and injuries in Punjab (India). Bd. 28, INJURY PREVENTION. BRITISH MED ASSOC HOUSE, TAVISTOCK SQUARE, LONDON WC1H 9JR, ENGLAND: BMJ PUBLISHING GROUP; 2022. S. 459–64.

82. Lari S, Yamagani P, Pandiyan A, Vanka J, Naidu M, Senthil Kumar B, u. a. The impact of the use of personal-protective-equipment on the minimization of effects of exposure to pesticides among farm-workers in India. Front Public Health. 2023;11:1075448.

83. Sekiyama M, Tanaka M, Gunawan B, Abdoellah O, Watanabe C. Pesticide usage and its association with health symptoms among farmers in rural villages in West Java, Indonesia. Environ Sci Int J Environ Physiol Toxicol. 2007;14 Suppl:23–33.

84. Perwitasari DA, Prasasti D, Supadmi W, Jaikishin SAD, Wiraagni IA. Impact of organophosphate exposure on farmers' health in Kulon Progo, Yogyakarta: Perspectives of physical, emotional and social health. SAGE Open Med. Dezember 2017;5:205031211771909.

85. Setyopranoto I, Argo IW, Ramadhani AF, Dwianingsih EK, Tama WN, Gofir A, u. a. The association between pesticide exposure and neurological signs and symptoms in farmers in Magelang district, Central Java, Indonesia. Open Access Maced J Med Sci. 2020;8(E):538–43.

86. Sodikin A, Muhyi R, Suhartono E, Husaini, Marlinae L. Analysis of factors related to pesticide poisoning in rice farmers in teweh selatan subdistrict, barito utara district. Indian J Forensic Med Toxicol. 2020;14(3):1715–20.

87. Ahmadi A, Pakravan N, Ghazizadeh Z. Pattern of acute food, drug, and chemical poisoning in Sari City, Northern Iran. Hum Exp Toxicol. September 2010;29(9):731–8.

88. Hashemi S, Rostami R, Hashemi M, Damalas C. Pesticide use and risk perceptions among farmers in southwest Iran. Hum Ecol Risk Assess Int J. 2012;18(2):456–70.

89. Soltaninejad K, Nelson LS, Bahreini SA, Shadnia S. Fatal aluminum phosphide poisoning in Tehran-Iran from 2007 to 2010. Indian J Med Sci. April 2012;66(3–4):66–70.

90. Afshari M, Poorolajal J, Assari M, Rezapur-Shahkolai F, Karimi-Shahanjarini A. Acute pesticide poisoning and related factors among farmers in rural Western Iran. Toxicol Ind Health. 2018;34(11):764–77.

91. Sharafi K, Pirsaheb M, Maleki S, Arfaeinia H, Karimyan K, Moradi M, u. a. Knowledge, attitude and practices of farmers about pesticide use, risks, and wastes; a cross-sectional study (Kermanshah, Iran). Sci Total Environ. 2018;645:509–17.

92. Sharifzadeh MS, Abdollahzadeh G, Damalas CA, Rezaei R, Ahmadyousefi M. Determinants of pesticide safety behavior among Iranian rice farmers. Sci Total Environ. 15. Februar 2019;651(Pt 2):2953–60.

93. Settimi L, Davanzo F, Carbone P, Sesana F, Locatelli C, Farina ML, u. a. Surveillance of toxic exposures: the pilot experience of the Poison Control Centers of Milan, Pavia and Bergamo in 2006. Ann Ist Super Sanita. 2007;43(3):287–94.

94. Ncube NM, Fogo C, Bessler P, Jolly CM, Jolly PE. Factors associated with self-reported symptoms of acute pesticide poisoning among farmers in northwestern Jamaica. Arch Environ Occup Health. 2011;66(2):65–74.

95. Eddleston M, Nagami H, Lin CY, Davis ML, Chang SS. Pesticide use, agricultural outputs, and pesticide poisoning deaths in Japan. Bd. 60, CLINICAL TOXICOLOGY. 2-4 PARK SQUARE, MILTON PARK, ABINGDON OR14 4RN, OXON, ENGLAND: TAYLOR & FRANCIS LTD; 2022. S. 933–41.

96. Mureithi P, Waswa F, Kituyi E. Assessment of occupational safety concerns in pesticide use among small-scale farmers in Sagana, Central Highlands, Kenya. In: Bationo A, Waswa B, Okeyo JM, Maina F, Kihara JM, Herausgeber. Innovations as Key to the Green Revolution in Africa. Dordrecht: Springer Netherlands; 2011. S. 993–8.

97. Macharia I. Pesticides and health in vegetable production in Kenya. BioMed Res Int. 2015;2015:241516.

98. Ngolo P, Nawwiri M, Machocho A, Oyieke H. Pesticides use in pest management: A case study of Ewaso Narok wetland small-scale vegetable farmers, Laikipia County, Kenya. J Agric Ecol Res Int. 2018;2(14):1–8.

99. Marete GM, Lalah JO, Mputhia J, Wekesa VW. Pesticide usage practices as sources of occupational exposure and health impacts on horticultural farmers in Meru County, Kenya. Bd. 7, HELIYON. THE BOULEVARD, LANGFORD LANE, KIDLINGTON, OXFORD OX5 1GB, OXON, ENGLAND: ELSEVIER SCI LTD; 2021.

100. Jallow MFA, Awadh DG, Albaho MS, Devi VY, Thomas BM. Pesticide knowledge and safety practices among farm workers in Kuwait: Results of a survey. Int J Environ Res Public Health. 24 2017;14(4).

101. Shattuck A. Risky subjects: Embodiment and partial knowledges in the safe use of pesticide. Geoforum. Juli 2021;123:153–61.

102. Abou Ibrahim S, Naji R, Zeineldeen H, Ghach W. Effectiveness of pesticide labels (pictograms and color codes): a cross-sectional study of farmers' understanding and practices in Lebanon. HUMAN AND ECOLOGICAL RISK ASSESSMENT. 530 WALNUT STREET, STE 850, PHILADELPHIA, PA 19106 USA: TAYLOR & FRANCIS INC; 2023.

103. Kasambala Donga T, Eklo OM. Environmental load of pesticides used in conventional sugarcane production in Malawi. Crop Prot. Juni 2018;108:71–7.

104. Nur AA, Rahmat A, Sa'ed Z, Sulastri S, Haslina H, Sazaroni M. Poisoning in children: a 4-year review of cases reported to the National Poisons Centre of Malaysia. J Med Toxicol Off J Am Coll Med Toxicol. 2012;8:208.

105. Leong YH, Ariff AM, Khan HRM, Rani NAA, Majid MIA. Paraquat poisoning calls to the Malaysia National Poison Centre following its ban and subsequent restriction of the herbicide from 2004 to 2015. J Forensic Leg Med. Mai 2018;56:16–20.

106. Tangiisuran B, Jiva M, Ariff AM, Abdul Rani NA, Misnan A, Rashid SM, u. a. Evaluation of types of poisoning exposure calls managed by the Malaysia National Poison Centre (2006–2015): A retrospective review. BMJ Open. Dezember 2018;8(12):e024162.

107. Ahmad ZNBS, Brison D, Van Tongeren M, Povey A. ACUTE HEALTH SYMPTOMS FOLLOWING PESTICIDES EXPOSURE AMONG FARMERS IN KELANTAN STATE, MALAYSIA. Occup Environ Med. 2023;80((Ahmad Z.N.B.S.) Department Medical Education, Department Public Health Medicine, Faculty of Medicine and Health Sciences, Universiti Malaysia, Sabah, Malaysia):A24–5.

108. Kamaruzaman NA, Leong YH, Jaafar MH, Mohamed Khan HR, Abdul Rani NA, Razali MF, u. a. Epidemiology and risk factors of pesticide poisoning in Malaysia: a retrospective analysis by the National Poison Centre (NPC) from 2006 to 2015. BMJ Open. 1. Juni 2020;10(6):e036048.

109. Sulaiman SKB, Ibrahim Y, Jeffree MS. Evaluating the perception of farmers towards pesticides and the health effect of pesticides: A cross-sectional study in the oil palm plantations of Papar, Malaysia. Interdiscip Toxicol. 2019;12(1):15–25.

110. Gonzalez-Santiago O, Morales-San Claudio P, Cantu-Cardenas L, Favela-Hernandez J. Unintentional and self-poisoning mortalities in Mexico, 2000-2012. PLoS ONE. 2017;12(7):e0181708.

111. Bernardino-Hernandez H, Mariaca-Mendez R, Nazar-Beutelspacher A, David Alvarez-Solis J, Torres-Dosal A, Herrera-Portugal C. KNOWLEDGE CONDUCT AND SYMPTOMS OF ACUTE INTOXICATION BY PESTICIDES BETWEEN PRODUCERS OF THREE AGRICULTURAL PRODUCTION SYSTEMS IN LOS ALTOS DE CHIAPAS,MEXICO. Bd. 35, REVISTA INTERNACIONAL DE CONTAMINACION AMBIENTAL. CIRCUITO EXTERIOR, MEXICO CITY CU 04510, MEXICO: CENTRO CIENCIAS ATMOSFERA UNAM; 2019. S. 7–23.

112. Esquivel-Valenzuela B, Antonio Cueto-Wong J, David Valdez-Cepeda R, Pedroza-Sandoval A, Trejo-Calzada R, Perez-Veyna O. PRACTICES OF HANDLING AND RISK ANALYSIS FOR THE USE OF PESTICIDES IN REGION LAGUNERA, MEXICO. Bd. 35, REVISTA INTERNACIONAL DE CONTAMINACION AMBIENTAL. CIRCUITO EXTERIOR, MEXICO CITY CU 04510, MEXICO: CENTRO CIENCIAS ATMOSFERA UNAM; 2019. S. 25–33.

113. Pinzaru I, Manceva T, Sircu R, Bahnarel I, Sanduleac E. Acute chemical poisonings in the Republic of Moldova: 5 years review. Chem J Mold. 2017;12(1):29–36.

114. Imane B, Mariam A, Chakib N, Ahmed Z, Samir EJ, Karima ER. Pesticide use pattern among farmers in a rural district of Meknes: Morocco. OALib. 2016;03(12):1–19.

115. Neupane D, Jørs E, Brandt L. Pesticide use, erythrocyte acetylcholinesterase level and self-reported acute intoxication symptoms among vegetable farmers in Nepal: a cross-sectional study. Environ Health [Internet]. 2014 [zitiert 28. Februar 2019];13(98). Verfügbar unter: https://ehjournal.biomedcentral.com/articles/10.1186/1476-069X-13-98

116. Singh SB, Pokharel PK, Raut P, Mehta K. Study of the effects of pesticide exposure among the workers of tea estates. Ann Glob Health. 12. März 2015;81(1):229.

117. Gyenwali D, Vaidya A, Tiwari S, Khatiwada P, Lamsal DR, Giri S. Pesticide poisoning in Chitwan, Nepal: a descriptive epidemiological study. BMC Public Health. 03 2017;17(1):619.

118. Bhandari G, Atreya K, Yang X, Fan L, Geissen V. Factors affecting pesticide safety behaviour: The perceptions of Nepalese farmers and retailers. Sci Total Environ. August 2018;631–632:1560–71.

119. Bhattarai S, Dhakal N, Poudyal A, Dhimal M. Health Impact Associated with Pesticides Use among Vegetables Farmers in Nepal: A Secondary Analysis. J Nepal Health Res Counc. 14. Dezember 2021;19(3):498–503.

120. Oluwole O, Cheke RA. Health and environmental impacts of pesticide use practices: a case study of farmers in Ekiti State, Nigeria. Int J Agric Sustain. August 2009;7(3):153–63.

121. Ugwu JA, Omoloye AA, Asogwa EU, Aduloju AR. Pesticide-handling practices among smallholder Vegetable farmers in Oyo state, Nigeria. Sci Res J. 2015;8.

122. Bassi A, Ramyil M, Ogundeko T, Abisoye-Ogunniyan A, Builders M, Thliza S, u. a. Farmer: Agrochemical use and associated risk factors in Fadan Daji District of Kaura LGA, Kaduna State, Nigeria. Am J Med Biol Res. 2016;4(3):33–41.

123. Adedire CO, Akinduro OO, Adeyemi JA. Health implications of pesticides application among cocoa farmers in Idanre local government area, Southwest Nigeria. Toxicol Ind Health. Mai 2023;39(5):258–66.

124. Kouser S, Qaim M. Impact of Bt cotton on pesticide poisoning in smallholder agriculture: A panel data analysis. Ecol Econ. 15. September 2011;70(11):2105–13.

125. Tahir S, Anwar T. Assessment of pesticide exposure in female population living in cotton growing areas of Punjab, Pakistan. Bull Environ Contam Toxicol. Dezember 2012;89(6):1138–41.

126. Bakhsh K, Ahmad N, Kamran MA, Hassan S, Abbas Q, Saeed R, u. a. Occupational hazards and health cost of women cotton pickers in Pakistani Punjab. BMC Public Health. 2016;16(961).

127. Elahi E, Weijun C, Zhang H, Nazeer M. Agricultural intensification and damages to human health in relation to agrochemicals: Application of artificial intelligence. Bd. 83, LAND USE POLICY. THE BOULEVARD, LANGFORD LANE, KIDLINGTON, OXFORD OX5 1GB, OXON, ENGLAND: ELSEVIER SCI LTD; 2019. S. 461–74.

128. Memon QUA, Wagan SA, Chunyu D, Shuangxi X, Jingdong L, Damalas CA. Health problems from pesticide exposure and personal protective measures among women cotton workers in southern Pakistan. Bd. 685, SCIENCE OF THE TOTAL ENVIRONMENT. RADARWEG 29, 1043 NX AMSTERDAM, NETHERLANDS: ELSEVIER; 2019. S. 659–66.

129. Khan A, Jaffar N, Murad F. Practice and effect of pesticide use on farmers' health in Sindh, Pakistan. J Pak Med Assoc. 2022;72(3):587–92.

130. Zyoud SH, Sawalha AF, Sweileh WM, Awang R, Al-Khalil SI, Al-Jabi SW, u. a. Knowledge and practices of pesticide use among farm workers in the West Bank, Palestine: safety implications. Environ Health Prev Med. Juli 2010;15(4):252–61.

131. Yasser EN. Acute toxicity among greenhouse farmers In Gaza Strip. IOSR J Dent Med Sci. 2016;15(11):109–17.

132. El-Nahhal Y. Risk factors among greenhouse farmers in Gaza Strip. Occup Dis Environ Med. 2017;05(01):1–10.

133. Lu JLDP. Occupational safety of farmers in the vegetable industry. Int J Occup Saf Ergon. 2011;17(4):445–53.

134. Lu JL, Cosca K. Pesticide application and health hazards: implications for farmers and the environment. Int J Environ Stud. April 2011;68(2):197–208.

135. Perez ICJ, Gooc CM, Cabili JR, Rico MJP, Ebasan MS, Zaragoza MJG, u. a. Pesticide use among farmers in Mindanao, Southern Philippines. Int J Bioflux Soc. 2015;7(1):19.

136. Del Prado-Lu JL. Insecticide residues in soil, water, and eggplant fruits and farmers' health effects due to exposure to pesticides. Environ Health Prev Med. Januar 2015;20(1):53–62.

137. Alzahrani S, Ibrahim N, Elnour M, Alqahtani A. Five-year epidemiological trends for chemical poisoning in Jeddah, Saudi Arabia. Ann Saudi Med. 2017;37(4):282–9.

138. Alnasser S, Hussain S, Kirdi T, Ahmed A. Aluminum phosphide poisoning in Saudi Arabia over a nine-year period. Ann Saudi Med. 2018;38(4):277–83.

139. Vucinic S, Jovanovic D, Antonijevic B, Vucinic Z, Djordjevic D, Potrebic O, u. a. Acute carbamate poisoning treated in the National Poison Control Centre during ten years period. Clin Toxicol. 2009;47(5):506–7.

140. Balme KH, Roberts JC, Glasstone M, Curling L, Rother HA, London L, u. a. Pesticide poisonings at a tertiary children's hospital in South Africa: an increasing problem. Clin Toxicol Phila Pa. November 2010;48(9):928–34.

141. Balme K, Stephen C. Streamlining referral decisions for childhood poisoning: a cross-sectional study from a tertiary children?s hospital in Cape Town, South Africa. Bd. 12, AFRICAN JOURNAL OF EMERGENCY MEDICINE. RADARWEG 29, 1043 NX AMSTERDAM, NETHERLANDS: ELSEVIER; 2022. S. 432–7.

142. Kim HJ, Cha ES, Ko Y, Kim J, Kim SD, Lee WJ. Pesticide poisonings in South Korea: findings from the National Hospital Discharge Survey 2004-2006. Hum Exp Toxicol. August 2012;31(8):751–8.

143. Lee WJ, Cha ES, Park J, Ko Y, Kim HJ, Kim J. Incidence of acute occupational pesticide poisoning among male farmers in South Korea. Am J Ind Med. September 2012;55(9):799–807.

144. Kim JH, Kim J, Cha ES, Ko Y, Kim DH, Lee WJ. Work-related risk factors by severity for acute pesticide poisoning among male farmers in South Korea. Int J Environ Res Public Health. 14. März 2013;10(3):1100–12.

145. Kim J, Ko Y, Lee WJ. Depressive symptoms and severity of acute occupational pesticide poisoning among male farmers. Occup Environ Med. Mai 2013;70(5):303–9.

146. Kim J, Shin DH, Lee WJ. Suicidal ideation and occupational pesticide exposure among male farmers. Environ Res. 2013;128:52–6.

147. Cha ES, Khang YH, Lee WJ. Mortality from and incidence of pesticide poisoning in South Korea: findings from National Death and Health Utilization Data between 2006 and 2010. PloS One. 2014;9(4):e95299.

148. Lee WJ, Ko Y, Cha ES. Acute pesticide poisoning among children in South Korea: findings from National Health Insurance claims data, 2006-2009. J Trop Pediatr. Februar 2014;60(1):4–9.

149. Moon JM, Chun BJ, Cho YS. The characteristics of emergency department presentations related to acute herbicide or insecticide poisoning in South Korea between 2011 and 2014. J Toxicol Environ Health A. 2016;79(11):466–76.

150. Kim J, Shin SD, Jeong S, Suh GJ, Kwak YH. Effect of prohibiting the use of Paraquat on pesticide-associated mortality. BMC Public Health. 2. November 2017;17(1):858.

151. Cha ES, Chang SS, Lee WJ. Potential underestimation of pesticide suicide and its impact on secular trends in South Korea, 1991-2012. Inj Prev J Int Soc Child Adolesc Inj Prev. 2016;22(3):189–94.

152. Moon J, Chun B, Cho Y, Lee S, Jung E. Characteristics of Emergency Department Presentations of Pediatric Poisoning between 2011 and 2016: A Retrospective Observational Study in South Korea. Pediatr Emerg Care. 2021;37(5):E261–8.

153. Shin J, Roh S. A study of risk factors for the possible cases of acute occupational pesticide poisoning of orchard farmers in some parts of south Chungcheong province. Bd. 31, ANNALS OF OCCUPATIONAL AND ENVIRONMENTAL MEDICINE. 101-1601, LOTTE CASTLE PRESIDENT, 109 MAPO-DAERO, SEOUL, MAPO-GU, SOUTH KOREA: KAMJE PRESS, XMLINK, CO; 2019.

154. Chien WC, Lin JD, Lai CH, Chung CH, Hung YC. Trends in poisoning hospitalization and mortality in Taiwan, 1999-2008: a retrospective analysis. BMC Public Health. 16. September 2011;11:703.

155. Chien WC, Chung CH, Lin CH, Lai CH. A nationwide evidence-based study of factors associated with hospitalisations due to unintentional poisoning and poisoning mortality in Taiwan. Int J Inj Contr Saf Promot. 2013;20(3):295–301.

156. Wu WT, Su TY, Chen JM, Wang CC, Chen WL. ASSESSING LONG-TERM TRENDS OF PESTICIDE POISONING IN AGRICULTURAL WORKERS AND PROHIBITED PESTICIDE POLICY IN TAIWAN. Occup Environ Med. 2023;80((Wu W.-T.; Su T.-Y.; Chen J.-M.) National Institute of Environmental Health Sciences, National Health Research Institutes, Miaoli, Taiwan):A40–1.

157. Lekei EE, Ngowi AV, London L. Farmers' knowledge, practices and injuries associated with pesticide exposure in rural farming villages in Tanzania. BMC Public Health. 2014;14(389).

158. Lekei E, Ngowi AV, London L. Hospital-based surveillance for acute pesticide poisoning caused by neurotoxic and other pesticides in Tanzania. Neurotoxicology. Dezember 2014;45:318–26.

159. da Silva M, Stadlinger N, Mmochi AJ, Stålsby Lundborg C, Marrone G. Pesticide use and self-reported health symptoms among rice farmers in Zanzibar. J Agromedicine. Oktober 2016;21(4):335–44.

160. Lekei EE, Ngowi AV, London L. Undereporting of acute pesticide poisoning in Tanzania: modelling results from two cross-sectional studies. Environ Health Glob Access Sci Source. 2016;15(118):118.

161. Lekei E, Ngowi A, London L. Acute pesticide poisoning in children: hospital review in selected hospitals of Tanzania. J Toxicol. 2017;2017:4208405.

162. Manyilizu WB, Mdegela RH, Helleve A, Skjerve E, Kazwala R, Nonga H, u. a. Self-reported symptoms and pesticide use among farm workers in Arusha, Northern Tanzania: A cross sectional study. Toxics. Dezember 2017;5(4).

163. Kachaiyaphum P, Howteerakul N, Sujirarat D, Siri S, Suwannapong N. Serum cholinesterase levels of Thai chilli-farm workers exposed to chemical pesticides: Prevalence estimates and associated factors. J Occup Health. 2010;52(1):89–98.

164. Sapbamrer R, Nata S. Health symptoms related to pesticide exposure and agricultural tasks among rice farmers from northern Thailand. Environ Health Prev Med. Januar 2014;19(1):12–20.

165. Sapbamrer R, Hongsibsong S, Kerdnoi T. Urinary dialkylphosphate metabolites and health symptoms among farmers in Thailand. Arch Environ Occup Health. 4. Mai 2017;72(3):145–52.

166. Thetkathuek A, Jaidee W. Factors that contribute to insecticide poisoning among immigrant agricultural workers in Thailand. Int J Occup Environ Health. 2. Januar 2017;23(1):60–70.

167. Kongtip P, Nankongnab N, Mahaboonpeeti R, Bootsikeaw S, Batsungnoen K, Hanchenlaksh C, u. a. Differences among Thai agricultural workers' health, working conditions, and pesticide use by farm type. Ann Work Expo Health. März 2018;62(2):167–81.

168. Ong-Artborirak P, Boonchieng W, Juntarawijit Y, Juntarawijit C. Potential Effects on Mental Health Status Associated with Occupational Exposure to Pesticides among Thai Farmers. Int J Environ Res Public Health [Internet]. 2022;19(15). Verfügbar unter: https://www.embase.com/search/results?subaction=viewrecord&id=L2018506345&from=export

169. Cevik C, Ozdemir R, Ari S. Occupational acute pesticide poisoning: a cross-sectional study of Turkish vegetable and fruit farmers based on self-reported symptoms and job characteristics. Med Lav. 31. August 2020;111(4):296–305.

170. Okonya JS, Kroschel J. A cross-sectional study of pesticide use and knowledge of smallholder potato farmers in Uganda. BioMed Res Int. 2015;2015:759049.

171. Clausen AS, Jørs E, Atuhaire A, Thomsen JF. Effect of integrated pest management training on Ugandan small-scale farmers. Environ Health Insights. Januar 2017;11:117863021770339.

172. Pedersen B, Ssemugabo C, Nabankema V, Jors E. Characteristics of pesticide poisoning in rural and urban settings in Uganda. Environ Health Insights. 5. Juni 2017;11.

173. Ssemugabo C, Halage AA, Neebye RM, Nabankema V, Kasule MM, Ssekimpi D, u. a. Prevalence and management of acute pesticide poisoning in public and private hospitals in Kampala, Uganda. Trop Med Int Health. Oktober 2017;22(1, SI):303–4.

174. Solomon C, Poole J, Palmer KT, Peveler R, Coggon D. Acute symptoms following work with pesticides. Occup Med Oxf Engl. Oktober 2007;57(7):505–11.

175. Perry L, Adams RD, Bennett AR, Lupton DJ, Jackson G, Good AM, u. a. National toxicovigilance for pesticide exposures resulting in health care contact - An example from the UK's National Poisons Information Service. Clin Toxicol Phila Pa. Juni 2014;52(5):549–55.

176. Yin S. Malicious use of nonpharmaceuticals in children. Child Abuse Negl. 2011;35(11):924–9.

177. Langley R, Mort S. Human exposures to pesticides in the United States. J Agromedicine. 2012;17(3):300–15.

178. Kamboj A, Spiller H, Casavant M, Chounthirath T, Smith G. Ocular exposures reported to United States Poison Control Centers. Ophthalmic Epidemiol. 2018;

179. Gummin DD, Mowry JB, Spyker DA, Brooks DE, Beuhler MC, Rivers LJ, u. a. 2018 Annual Report of the American Association of Poison Control Centers' National Poison Data System (NPDS): 36th Annual Report. Clin Toxicol. 2. Dezember 2019;57(12):1220–413.

180. Gummin DD, Mowry JB, Beuhler MC, Spyker DA, Brooks DE, Dibert KW, u. a. 2019 Annual Report of the American Association of Poison Control Centers' National Poison Data System (NPDS): 37th Annual Report. Clin Toxicol. 1. Dezember 2020;58(12):1360–541.

181. Gummin DD, Mowry JB, Beuhler MC, Spyker DA, Bronstein AC, Rivers LJ, u. a. 2020 Annual Report of the American Association of Poison Control Centers' National Poison Data System (NPDS): 38th Annual Report. Clin Toxicol. 2. Dezember 2021;59(12):1282–501.

182. Gummin DD, Mowry JB, Beuhler MC, Spyker DA, Rivers LJ, Feldman R, u. a. 2021 Annual Report of the National Poison Data System © (NPDS) from America's Poison Centers: 39th Annual Report. Clin Toxicol. 2. Dezember 2022;60(12):1381–643.

183. Gummin DD, Mowry JB, Beuhler MC, Spyker DA, Rivers LJ, Feldman R, u. a. 2022 Annual Report of the National Poison Data System ® (NPDS) from America's Poison Centers ® : 40th Annual Report. Clin Toxicol. 3. Oktober 2023;61(10):717–939.

184. Lee SJ, Mulay P, Diebolt-Brown B, Lackovic MJ, Mehler LN, Beckman J, u. a. Acute illnesses associated with exposure to fipronil—surveillance data from 11 states in the United States, 2001–2007. Clin Toxicol. August 2010;48(7):737–44.

185. Lee SJ, Mehler L, Beckman J, Diebolt-Brown B, Prado J, Lackovic M, u. a. Acute pesticide illnesses associated with off-target pesticide drift from agricultural applications: 11 States, 1998-2006. Environ Health Perspect. August 2011;119(8):1162–9.

186. Kasner E, Keralis J, Mehler L, Beckman J, Bonnar-Prado, Lee, u. a. Gender differences in acute pesticide-related illnesses and injuries among farmworkers in the United States, 1998-2007. Am J Ind Med. 2012;55(7):571–83.

187. Spiller HA, Beuhler MC, Ryan ML, Borys DJ, Aleguas A, Bosse GM. Evaluation of changes in poisoning in young children: 2000 to 2010. Pediatr Emerg Care. Mai 2013;29(5):635–40.

188. Hudson NL, Kasner EJ, Beckman J, Mehler L, Schwartz A, Higgins S, u. a. Characteristics and magnitude of acute pesticide-related illnesses and injuries associated with pyrethrin and pyrethroid exposures--11 states, 2000-2008. Am J Ind Med. Januar 2014;57(1):15–30.

189. Calvert GM, Beckman J, Prado JB, Bojes H, Schwartz A, Mulay P, u. a. Acute occupational pesticide-related illness and injury - United States, 2007–2011. MMWR Morb Mortal Wkly Rep. 14. Oktober 2016;63(55):11–6.

190. Higgins S, Langley R, Buhler WG. Establishment of the North Carolina Pesticide Incident Surveillance Program and the integration of its findings into pesticide safety education programs. J Pestic Saf Educ. 2016;18:12–28.

191. Namulanda G, Monti MM, Mulay P, Higgins S, Lackovic M, Schwartz A, u. a. Acute nonoccupational pesticide-related illness and injury — United States, 2007–2011. MMWR Morb Mortal Wkly Rep. 14. Oktober 2016;63(55):5–10.

192. Trueblood AB, Shipp E, Han D, Ross J, Cizmas LH. Pesticide-Related Hospitalizations Among Children and Teenagers in Texas, 2004-2013. Public Health Rep Wash DC 1974. August 2016;131(4):588–96.

193. Liu R, Alarcon WA, Calvert GM, Aubin KG, Beckman J, Cummings KR, u. a. Acute illnesses and injuries related to total release foggers - 10 States, 2007-2015. MMWR Morb Mortal Wkly Rep. 2. Februar 2018;67(4):125–30.

194. Capper GH, Landsteiner AMK. Pesticide Poisonings in Minnesota, 2000-2015. Bd. 82, JOURNAL OF ENVIRONMENTAL HEALTH. 720 S COLORADO BLVD SUITE 970, SOUTH TOWER, DENVER, CO 80246 USA: NATL ENVIRON HEALTH ASSOC; 2019. S. 16–23.

195. Kyeremateng-Amoah E, Friedman L, Wahl M, Forst L. Public Health Surveillance for the Prevention of Pesticide-Related Illness in Illinois. J Occup Environ Med. 2020;62(5):359–69.

196. VanWormer JJ, Berg RL, Burke RR, Barnes KL, Weichelt BP. Regional surveillance of medically-attended farm-related injuries in children and adolescents. Front Public Health. 2022;10:1031618.

197. Parks CG, Leyzarovich D, Love SA, Long S, Hofmann JN, Beane Freeman LE, u. a. High pesticide exposures events, pesticide poisoning, and shingles: A medicare-linked study of pesticide applicators in the agricultural health study. Environ Int. November 2023;181:108251.

198. Gomez FMJ, Caceres GJL. Toxicity by organophosphate insecticides of fumigators working on the dengue control campaign in Aragua state, Venezuela, 2008. Boletin Malariol Salud Ambient. Juli 2010;50(1):119–25.

199. Thong TA, Phong LT. Impacts of pesticide application on the health of hired applicators in Angiang province, Vietnam. Research Center for Rural Development, An Giang University, Vietnam; 2011.

200. Z'gambo J, Siulapwa Y, Michelo C. Pattern of acute poisoning at two urban referral hospitals in Lusaka, Zambia. BMC Emerg Med. 2016;16(2).

201. Magauzi R, Mabaera B, Rusakaniko S, Chimusoro A, Ndlovu N, Tshimanga M, u. a. Health effects of agrochemicals among farm workers in commercial farms of Kwekwe district, Zimbabwe. Pan Afr Med J. 2011;9:26.
