# Supplementary material for: Worldwide unintentional acute pesticide poisonings: reassessing the occupational and non-occupational burden
Source: Front Public Health. 2026 Sep 2;14:1825899. doi: 10.3389/fpubh.2026.1825899 (PMC13578083; doi:10.3389/fpubh.2026.1825899)
Supplement: Supplementary file 5 [file Data_sheet_4.DOCX]

S4 UAPP: MS-Excel sheet for data extraction

| **Item** | **input_column and drop-down items** |
| --- | --- |
| **Publication** |  |
| authors |  |
| title of article |  |
|  |  |
| **Study characteristics** |  |
| country |  |
| years covered |  |
| region covered | national / district |
| pesticides covered | extracted data apply to all pesticides, specific data not given / extracted data apply to all pesticides, additional specific data given / extracted data apply to group of pesticides or active ingredients |
| population covered | all / farmers only / workers only / farmers & workers / children |
| study type | survey / PCC / hospital / register |
|  |  |
| **Case definition** |  |
| number of symptoms used for case definition | at least one / 2 or more / unspecified |
| latency period: symptoms within … | 24 h / 48 h / other / not specified |
| at-risk time | annual / ever / other/ not specified |
|  |  |
| **Study outcome** |  |
| outcome type | mortality / morbidity / both |
| type poisoning | accidental / occupational / homicidal - malicious / mixed |
|  |  |
| **Study results** |  |
| no. of fatalities per year |  |
| no. non-fatal cases per year |  |
| size of population or sample |  |
| fatalities per year per population (%) |  |
| non-fatalities per year per population (%) |  |
|  |  |
| **Risk-of bias assessment** |  |
| selection of study population | no selection / representative sample / simple sample or not stated |
| diagnoses of poisoning | ICD codes / by study scientist / self-reported / not stated |
| laboratory evaluation | identification of active ingredients / biomarkers like cholinesterase / no or not stated |
|  |  |
| **Decision** |  |
| 1. reviewer | EM / MW / PC / WB |
| final decision 1 review | data ok / exclude, why … |
| 2. reviewer | EM / MW / PC / WB |
| final decision 2 review | data ok / exclude, why … |
