# Supplementary material for: Worldwide unintentional acute pesticide poisonings: reassessing the occupational and non-occupational burden
Source: Front Public Health. 2026 Sep 2;14:1825899. doi: 10.3389/fpubh.2026.1825899 (PMC13578083; doi:10.3389/fpubh.2026.1825899)
Supplement: Supplementary file 6 [file Data_sheet_5.DOCX]

**Table S5: Countries covered in review by regions and data sources**

| No | Region | Country Name | Data source^1^ |
| --- | --- | --- | --- |
| 1 | Africa, Eastern | Ethiopia | P |
| 2 |  | Kenya | P |
| 3 |  | Malawi | P |
| 4 |  | Mauritius | WX |
| 5 |  | Mayotte | WX |
| 6 |  | Reunion | WX |
| 7 |  | Tanzania | PW |
| 8 |  | Uganda | P |
| 9 |  | Zimbabwe | P |
| 10 | Africa, Middle-Southern | Cameroon | P |
| 11 |  | South Africa | PW |
| 12 | Africa, Northern | Egypt, Arab Rep. | PW |
| 13 |  | Libya | W |
| 14 |  | Morocco | PW |
| 15 |  | Tunisia | WX |
| 16 | Africa, Western | Burkina Faso | P |
| 17 |  | Cote d'Ivoire | P |
| 18 |  | Gambia, The | P |
| 19 |  | Ghana | P |
| 20 |  | Nigeria | P |
| 21 |  | Senegal | P |
| 22 | America, Caribbian | Anguilla | WX |
| 23 |  | Antigua and Barbuda | WX |
| 24 |  | Aruba | WX |
| 25 |  | British Virgin Islands | WX |
| 26 |  | Cayman Islands | WX |
| 27 |  | Cuba | WX |
| 28 |  | Dominica | WX |
| 29 |  | Dominican Republic | WX |
| 30 |  | Grenada | WX |
| 31 |  | Guadeloupe | WX |
| 32 |  | Jamaica | PW |
| 33 |  | Martinique | WX |
| 34 |  | Montserrat | WX |
| 35 |  | Puerto Rico | WX |
| 36 |  | St. Lucia | WX |
| 37 |  | St. Vincent and the Grenadines | WX |
| 38 |  | Turks and Caicos Islands | WX |
| 39 | America, Central | Costa Rica | PWX |
| 40 |  | El Salvador | WX |
| 41 |  | Guatemala | WX |
| 42 |  | Mexico | PWX |
| 43 |  | Nicaragua | WX |
| 44 |  | Panama | WX |
| 45 | America, Northern | Bermuda | WX |
| 46 |  | Canada | PWX |
| 47 |  | United States | PWX |
| 48 | America, South | Argentina | PWX |
| 49 |  | Bolivia | P |
| 50 |  | Brazil | PWX |
| 51 |  | Chile | PWX |
| 52 |  | Colombia | PWX |
| 53 |  | Ecuador | PWX |
| 54 |  | French Guiana | WX |
| 55 |  | Guyana | WX |
| 56 |  | Paraguay | WX |
| 57 |  | Peru | WX |
| 58 |  | Uruguay | WX |
| 59 |  | Venezuela, RB | PW |
| 60 | Asia, Central | Kazakhstan | WX |
| 61 |  | Kyrgyz Republic | WX |
| 62 |  | Tajikistan | W |
| 63 |  | Uzbekistan | W |
| 64 | Asia, Eastern | China | PW |
| 65 |  | Japan | PWX |
| 66 |  | Korea, Rep. | PWX |
| 67 |  | Mongolia | WX |
| 68 |  | Taiwan | P |
| 69 | Asia, South-eastern | Brunei Darussalam | WX |
| 70 |  | Cambodia | P |
| 71 |  | Indonesia | P |
| 72 |  | Lao PDR | P |
| 73 |  | Malaysia | PW |
| 74 |  | Philippines | PW |
| 75 |  | Singapore | WX |
| 76 |  | Thailand | PWX |
| 77 |  | Viet Nam | P |
| 78 | Asia, Southern | Bangladesh | P |
| 79 |  | India | PW |
| 80 |  | Iran, Islamic Rep. | PWX |
| 81 |  | Maldives | WX |
| 82 |  | Nepal | P |
| 83 |  | Pakistan | P |
| 84 |  | Sri Lanka | WX |
| 85 | Asia, Western | Armenia | WX |
| 86 |  | Cyprus | WX |
| 87 |  | Georgia | PWX |
| 88 |  | Israel | WX |
| 89 |  | Jordan | WX |
| 90 |  | Kuwait | PWX |
| 91 |  | Lebanon | PWX |
| 92 |  | Oman | WX |
| 93 |  | Qatar | W |
| 94 |  | Saudi Arabia | PWX |
| 95 |  | Turkiye | PWX |
| 96 |  | United Arab Emirates | WX |
| 97 |  | West Bank and Gaza | PW |
| 98 | Europe, Eastern | Belarus | WX |
| 99 |  | Bulgaria | WX |
| 100 |  | Czechia | WX |
| 101 |  | Hungary | PWX |
| 102 |  | Moldova | PWX |
| 103 |  | Poland | WX |
| 104 |  | Romania | WX |
| 105 |  | Slovak Republic | WX |
| 106 | Europe, Northern | Denmark | WX |
| 107 |  | Estonia | WX |
| 108 |  | Finland | W |
| 109 |  | Iceland | WX |
| 110 |  | Ireland | WX |
| 111 |  | Latvia | WX |
| 112 |  | Lithuania | WX |
| 113 |  | Norway | WX |
| 114 |  | Sweden | WX |
| 115 |  | United Kingdom | PWX |
| 116 | Europe, Southern | Bosnia and Herzegovina | W |
| 117 |  | Croatia | WX |
| 118 |  | Greece | WX |
| 119 |  | Italy | PWX |
| 120 |  | Malta | WX |
| 121 |  | Montenegro | WX |
| 122 |  | North Macedonia | W |
| 123 |  | Portugal | PWX |
| 124 |  | Serbia | PW |
| 125 |  | Slovenia | W |
| 126 |  | Spain | PWX |
| 127 | Europe, Western | Austria | WX |
| 128 |  | Belgium | WX |
| 129 |  | France | PWX |
| 130 |  | Germany | PWX |
| 131 |  | Luxembourg | WX |
| 132 |  | Netherlands | WX |
| 133 |  | Switzerland | WX |
| 134 | Oceania, AUS NZ Mic-Mel-Polynesia | Australia | PWX |
| 135 |  | Micronesia, Fed. Sts. | WX |
| 136 |  | Papua New Guinea | W |
| 137 |  | Solomon Islands | WX |

1) Data source: P= Publication, W = WHO fatal general population, X= WHO fatal farming population
